# Causal Associations of Inflammatory Cytokines With Osteosarcopenia: Insights From Mendelian Randomization and Single Cell Analysis

**DOI:** 10.1155/mi/6005225

**Published:** 2025-04-03

**Authors:** Zugui Wu, Jiyong Yang, Yue Zhu, Jiao Li, Kang Xu, Yuanlong Li, Guoqing Zhong, Yanfei Xu, Ying Guo, Yu Zhang

**Affiliations:** ^1^Department of Bone Tumor, Guangdong Cardiovascular Institute, Guangdong Provincial People's Hospital (Guangdong Academy of Medical Sciences), Southern Medical University, Guangzhou 510000, Guangdong, China; ^2^Department of Orthopaedic, The Third Affiliated Hospital of Yunnan University of Chinese Medicine, Kunming Municipal Hospital of Traditional Chinese Medicine, Kunming 650000, Yunnan, China; ^3^Department of Orthopaedic, The Fifth Clinical College of Guangzhou University of Chinese Medicine, Guangzhou 510000, Guangdong, China; ^4^Department of Orthopaedic, Shenzhen Hospital of Guangzhou University of Chinese Medicine (Futian), Shenzhen Research Institute of Guangzhou University of Traditional Medicine (Futian), Shenzhen 518000, Guangdong, China; ^5^Department of Bone Tumor, Guangdong Provincial People's Hospital, Guangdong Academy of Medical Sciences, Southern Medical University, Guangzhou 510000, Guangdong, China

**Keywords:** cytokines, inflammation, mendelian randomized, osteosarcopenia, single-cell sequencing

## Abstract

**Background:** Osteosarcopenia, the coexistence of osteoporosis and sarcopenia, poses significant challenges in aging populations due to its dual impact on bone and muscle health. Inflammation, mediated by specific cytokines, is thought to play a crucial role in the development of osteosarcopenia, though the underlying mechanisms are not fully understood.

**Objective:** This study aimed to clarify the causal role of circulating cytokines in the pathogenesis of osteosarcopenia by employing mendelian randomization (MR) and single-cell RNA sequencing (scRNA-seq) to identify cell-specific cytokine expression patterns. The ultimate objective was to uncover potential pathological mechanisms and therapeutic targets for treating osteosarcopenia.

**Methods:** A two-sample MR approach was employed, leveraging publicly available genome-wide association study (GWAS) data from multiple cohorts. A total of 91 circulating cytokines were examined using genetic instruments, and their causal effects on traits related to osteoporosis and sarcopenia were evaluated. Various complementary and sensitivity analyses were performed to ensure robust findings. Additionally, scRNA-seq datasets from human muscle and bone marrow were analyzed to validate the single-cell expression profiles of candidate cytokines.

**Results:** MR analysis identified several cytokines with causal effects on osteosarcopenia traits, including LTA, CD40, CXCL6, CXCL10, DNER (delta and notch-like epidermal growth factor-related receptor), and VEGFA (vascular endothelial growth factor A). LTA and CD40 were protective for both bone and muscle, while VEGFA posed a risk. Other cytokines demonstrated opposite effects on bone and muscle. Single cell analysis revealed distinct expression patterns, with LTA highly expressed in lymphocytes, CD40 in immune cells, and VEGFA in various musculoskeletal cell types. Age-related differences in cytokine expression were also noted, with LTA more highly expressed in younger individuals, and VEGFA in older individuals.

**Conclusion:** This study offers preliminary insights into the inflammatory mechanisms potentially driving osteosarcopenia, identifying key cytokines that may be involved in its pathogenesis. By integrating MR and scRNA-seq data, we highlight potential therapeutic targets, though further research is needed to confirm these findings and their implications for musculoskeletal health.

## 1. Introduction

Osteoporosis and sarcopenia are two prevalent conditions associated with aging, each independently contributing significantly to morbidity and mortality among the elderly [1, 2]. Osteoporosis, characterized by a decrease in bone mass and density, increases susceptibility to fractures (FXs) [1]. On the other hand, sarcopenia, which involves the loss of skeletal muscle mass and strength, leads to functional impairments and an increased risk of physical disability [2]. Both conditions not only compromise the quality of life but also impose substantial healthcare burdens due to the increased need for medical care and rehabilitation [3, 4]. As the global population continues to age, the concomitant occurrence of osteoporosis and sarcopenia in the same individuals has given rise to a new clinical concept known as osteosarcopenia [5–8]. The dual impact of weakened bones and muscle loss makes osteosarcopenia a particularly challenging condition to manage, as it enhances the frailty and vulnerability of affected individuals significantly more than either condition alone.

While recent years have seen an increase in research on osteosarcopenia, our grasp of its underlying pathophysiology continues to be limited [4, 7, 9, 10]. The development of osteosarcopenia is governed by complex interactions among mechanical, biochemical, genetic, and lifestyle factors that affect the musculoskeletal system, yet a comprehensive understanding of these factors' precise roles and interactions has yet to be achieved [11–14]. As the understanding of osteosarcopenia's pathophysiology continues to evolve, a detailed comprehension of the overlapping pathophysiological mechanisms of osteoporosis and sarcopenia is crucial. This insight is essential for clarifying their intertwined pathology and propelling the development of effective treatments for osteosarcopenia.

Inflammation stands out as a critical pathological mechanism shared by both osteoporosis and sarcopenia, and it likely plays a significant role in the onset of osteosarcopenia [1, 2]. Chronic low-grade inflammation, often referred to as inflammaging, is marked by elevated levels of systemic inflammatory cytokines and is known to contribute to the progressive decline in both muscle mass and bone density [15]. Recent studies have explored the relationship between specific inflammatory cytokines and both sarcopenia and osteoporosis, respectively. Notably, cytokines like TNF-*α* and IL-6 have been shown to promote muscle catabolism and increase bone resorption in animal models. Furthermore, observational cohort, cross-sectional, and case-control studies in humans have also highlighted a significant association between these cytokines and the pathogenesis of osteoporosis and sarcopenia [16–18]. However, despite this insight, the compounded effects of inflammation in osteosarcopenia, particularly in elderly individuals, have been less frequently examined. Previous research has primarily focused on either osteoporosis or sarcopenia in isolation, leaving a significant gap in our understanding of how these conditions interact when they occur concurrently.

Mendelian randomization (MR) studies are notable for their ability to reduce biases from confounding factors and reverse causation by using genetic variants as IVs, thus emulating the environment of randomized controlled trials [19]. This methodology enhances the reliability of causal inferences in epidemiological research, making it a powerful tool for exploring complex interactions between genetics and various health conditions. Currently, a substantial body of MR research has independently examined osteoporosis and sarcopenia [20–24]. These investigations have significantly advanced our understanding of the genetic determinants influencing these conditions and their interactions with multiple factors. However, these studies often overlook the compounded effects when both conditions coexist, as frequently observed in clinical settings among the elderly population.

To address this research gap, the present study employed the MR approach to explore the potential causal roles of circulating inflammatory cytokines in the pathogenesis of osteosarcopenia, with the aim of providing valuable insights for future therapeutic interventions. As an exploratory investigation, our study sought to identify a broad spectrum of cytokines potentially involved in the development of osteosarcopenia. Furthermore, we leverage single-cell sequencing datasets to examine the cell-specific expression of the candidate cytokines identified through MR analysis, thereby deepening our understanding of the mechanistic regulation of musculoskeletal metabolism by these cytokines.

## 2. Methods

### 2.1. Study Design

**Figure 1:**
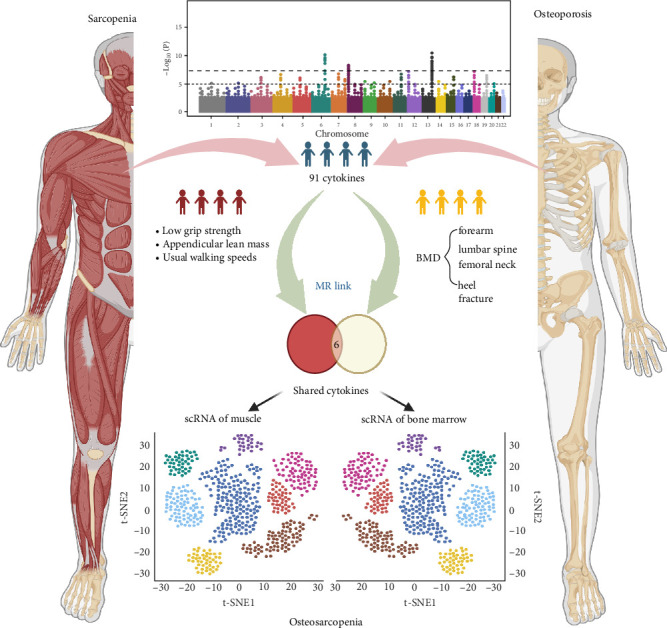
The work flowchart of the current study.

This study followed STROBE-MR guidelines and employed a two-sample MR approach, using publicly available genome-wide association study (GWAS) summary data from May 20, 2024 [25]. Anonymized data negated the need for further ethics approval or participant consent, in line with the original GWAS protocols. As no GWAS specifically exists for osteosarcopenia, the study utilized GWAS data related to osteoporosis and sarcopenia, following established MR practices in sarcopenia research. Furthermore, single-cell RNA-sequencing (scRNA-seq) was conducted using publicly available datasets from human muscle and bone marrow (BM) to identify the cell-specific expression patterns of cytokines highlighted by the MR analysis. This integration of scRNA-seq allowed for validation of the cytokines' cellular expression, enhancing the study's mechanistic insights. The study design flowchart is shown in Figure 1.

### 2.2. Data Source for MR Analysis

Circulating cytokine levels were examined in a recent study, which included 11 cohorts and a total of 14,824 participants of European descent, representing the most comprehensive dataset available [26]. This study utilized well-established methodologies and data control techniques, measuring 91 cytokines using the Olink Target Inflammation panel and collecting comprehensive genome-wide genetic data.

We utilized diverse GWAS datasets from the Genetic Factors for Osteoporosis Consortium (GEFOS) pertaining to osteoporosis-related traits (http://www.gefos.org/). These datasets included measurements of bone mineral density (BMD) at the forearm (FA), femoral neck (FN), and lumbar spine (LS), as well as estimates from quantitative heel ultrasounds and associated FX risk assessments. The 2015 Data Release encompassed meta-analyses of BMD for the FA, FN, and LS, involving sample sizes of 8143, 32,735, and 28,498, respectively [27]. Additionally, the 2017 UK UKBB eBMD GWAS data release analyzed BMD derived from quantitative heel ultrasounds based on UK Biobank (heel bone mineral density, HBMD), with a substantial cohort of 426,824 participants [28]. The 2018 FX GWAS release provided summary data across 25 cohorts, incorporating genome-wide genotyping and FX data, cumulatively accounting for 37,857 cases and 227,116 controls [29].

The summary GWAS data for sarcopenia-related traits included low hand grip strength (LGS), adjusted appendicular lean mass (ALM), and usual walking speeds (WSs). The data on LGS, based on the EWGSOP definition (male: <30 kg; female: <20 kg), included 254,894 individuals aged 60 years or older from 22 cohorts, primarily from the UK Biobank [30]. For the ALM cohort, body composition was measured by bioelectric impedance analysis (BIA), with 450,243 individuals aged 48–73 included [31]. The summary GWAS data on WS involved 459,915 European participants from the UK Biobank public database (Table 1).

### 2.3. Genetic Instruments

The fundamental premises of MR analysis include: (1) SNPs must exhibit a strong correlation with the exposure; (2) SNPs should have no direct correlation with the outcome; and (3) SNPs should not be associated with any confounders. In this study, populations within the dataset used for the exposure and outcome variables were independent of each other, meeting the basic assumption of a two-sample MR study. We employed stringent criteria for our instrumental variables (IVs): (1) in the main analysis A, the significance threshold of IVs associated with exposures was set at *p*  < 5 × 10^−8^. To ensure a more comprehensive evaluation of the cytokines, we implemented a less stringent significance threshold of *p*  < 5 × 10^−6^. This adjusted threshold, referred to as analysis B, allowed for the inclusion of a greater number of IVs, thereby enhancing the robustness and reliability of the MR analyses. This approach is consistent with methodologies used in previous studies, which have also adopted a more relaxed threshold to ensure the inclusion of a sufficient number of genetic variants for meaningful analysis [20]; (2) linkage disequilibrium (LD) *r*^2^ not exceeding 0.001; and (3) a clumping distance of 10,000 kb. All SNPs linked to outcomes with a *p*-value below 0.05 were excluded. Each SNP was scrutinized using the PhenoScanner database to exclude confounding factors such as age and gender [32]. The “MR-PRESSO” method, implemented through the R package “MRPRESSO,” was applied to detect horizontal pleiotropy and identify outliers [33]. SNPs with palindromic sequences were removed to ensure the validity of the IVs. Lastly, the *F*-statistic was computed to assess the statistical power of the selected IVs.

### 2.4. MR Analysis

In this analysis, we employed the inverse-variance weighted (IVW) method as the cornerstone of our statistical approach due to its robustness. The employment of a random effects model, rooted in the IVW method, was particularly valuable in contexts marked by high SNP heterogeneity, ensuring more reliable results. We analyzed the causal links between circulating cytokine levels and the risk of osteosarcopenia, utilizing beta values or odds ratios (ORs) based on the outcome metrics. To bolster the integrity of our findings, we integrated several auxiliary methodologies. The MR-Egger method, critical for identifying pleiotropic effects, was validated with a significance threshold of *p*  < 0.05. The weighted median approach was instrumental when a significant proportion of the analysis relied on potentially invalid IVs. The simple statistical models could avoid the complexities of multivariable adjustments. In addition, weighted models were used to enhance the precision of our conclusions by prioritizing data based on reliability.

### 2.5. Sensitivity Analysis

To ensure the robustness and validity of our findings, we conducted various sensitivity analyses and statistical tests. Cochran's *Q* test was utilized to assess the heterogeneity among the SNPs, with a *p*  > 0.05 indicating an absence of significant heterogeneity. In cases where significant heterogeneity was detected, a random effects model was applied; otherwise, a fixed effects model was used. To address potential violations of MR assumptions, specifically horizontal pleiotropy, we employed both the MR-PRESSO global test and MR-Egger intercept. The MR-Egger intercept, which estimates the average pleiotropic effect, was considered significant if the *p*-value was less than 0.05. This approach allowed us to generate a robust pleiotropy-adjusted MR estimate. In addition, the MR-PRESSO outlier test was employed to identify and correct for horizontal pleiotropy by removing or down-weighting SNPs exhibiting significant pleiotropic effects (*p*-value of the MR-PRESSO global test <0.05). Furthermore, the MR-PRESSO distortion test was implemented to evaluate the presence of significant distortions in causal estimates both before and after the removal of outliers. We also performed leave-one-out analysis to assess whether any individual SNP had an extreme influence on the causal estimates. This step further enhanced the reliability and robustness of our results, ensuring that our findings were not unduly influenced by any single SNP, thereby validating the overall stability of our conclusions. Lastly, causal directionality was assessed using the Steiger filtering method to check for potential reverse causation. The results were categorized as follows: “true” if the causal direction from exposure to outcome was significant (*p*  < 0.05), “false” if reverse causation was significant (*p*  < 0.05), and “uncertain” if *p* ≥ 0.05, to provide clarity in the interpretation of causal relationships.

### 2.6. scRNA-Seq Analysis

The candidate cytokines were selected based on their identification in both analyses A and B as factors showing causal relationships with both osteoporosis and sarcopenia traits. To establish the mechanistic role of each candidate cytokine in musculoskeletal metabolism, which is crucial for leveraging these genetic determinants as therapeutic targets in translational studies, we employed publicly available scRNA-seq datasets. These datasets were used to investigate the expression of candidate cytokines at the single-cell level, enabling a more granular understanding of how these cytokines contribute to musculoskeletal health and disease. The scRNA-seq data utilized in this study were sourced from the publicly available Human Muscle Ageing Cell Atlas database (https://db.cngb.org/cdcp/hlma/), which includes single-cell/single-nucleus transcriptomic and chromatin accessibility data of human limb skeletal muscles [34]. The dataset contains over 387,000 cells/nuclei derived from 31 individuals aged 15–99 years, representing various levels of physical fitness and frailty. The processing pipeline used by Lai et al. [34] involved standard preprocessing steps including quality control (QC) filters based on unique molecular identifiers (UMIs) (>1000), gene (>500), and mitochondrial gene percentages (<5%). Following QC, the data were normalized using total count normalization and log-transformed to adjust for sequencing depth differences across cells. Batch-effect corrections were applied using the Harmony algorithm. The cell annotation was provided by the authors.

For osteoporosis, we utilized the BM scRNA sequencing dataset of healthy adults collected by Lee et al. [35]. We selected those data with age information and older than 18 years for subsequent analysis. Forty-eight individuals were eventually screened for compliance. We adopted age 50 as a cutoff to categorize the samples into young and aged groups. The scRNA sequencing data were processed and analyzed in accordance with Lee et al. [35]. Specifically, the raw reads were subjected to QC based on UMI counts (>1000), the number of detected genes (>500), and the proportion of mitochondrial and ribosomal genes (<5%) to filter out low-quality cells. FastIntegration tool was adopted to remove the significant batch effects. After that, unsupervised clustering and differential gene expression analysis were conducted. The cell annotation was provided by the authors.

### 2.7. Statistics and Graphs

MR analyses were conducted using the “TwoSampleMR” and “MR-PRESSO” and the scRNA analysis was conducted via “Seurat” and “plot1cell” packages in R 4.4.0. All *p*-values < 0.05 were considered statistically significant, as the primary goal of this study was to explore potential associations between genes and phenotypes rather than to test specific hypotheses. The graphs were generated using GraphPad Prism 9.2.0 (GraphPad Software, Inc., San Diego, CA, USA), R version 4.4.0, and BioRender.com.

## 3. Results

### 3.1. Main Analysis A

**Figure 2:**
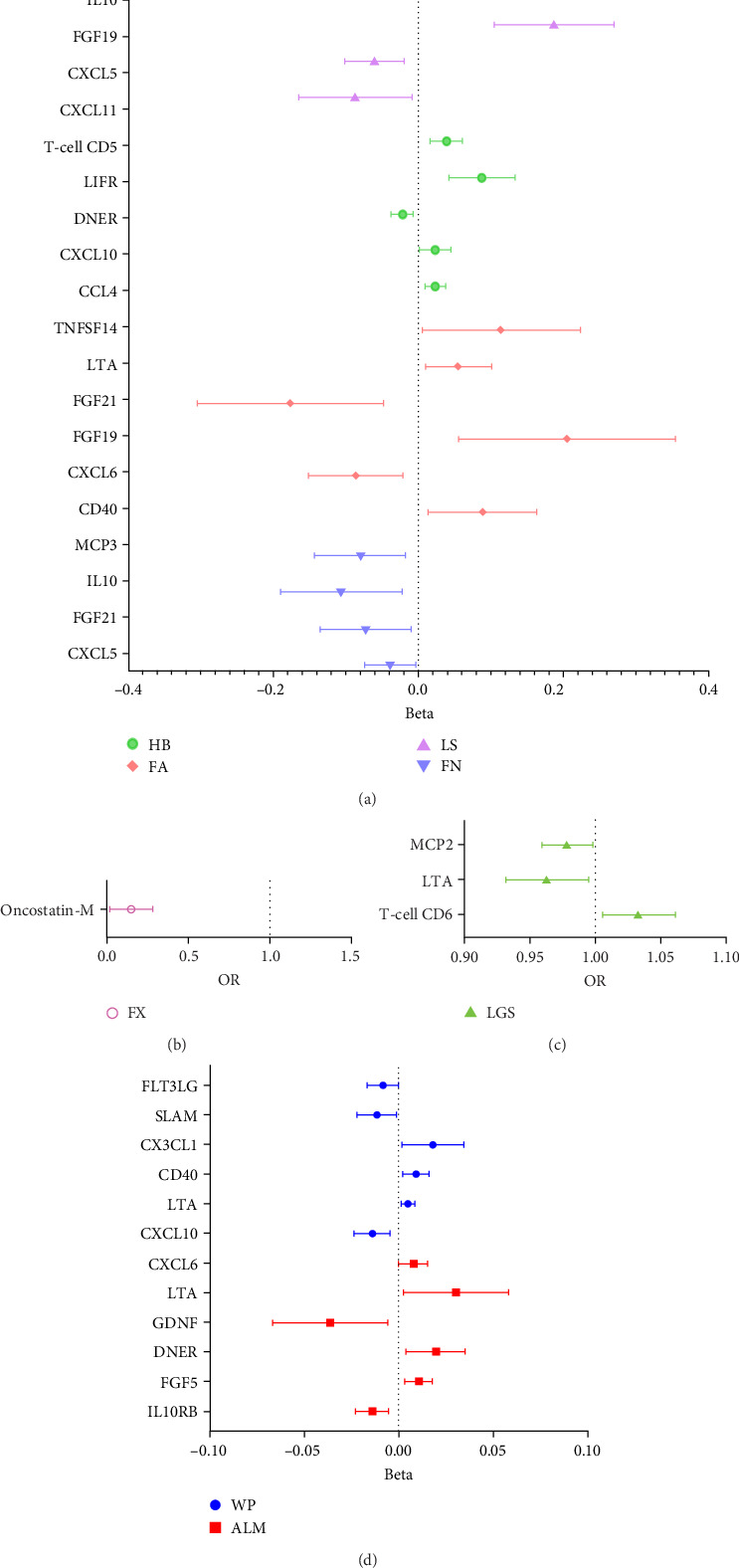
Forest plot results for main analysis A. (A, B) The results of osteoporosis traits; (C, D) the results of sarcopenia traits. ALM, appendicular lean mass; FA, forearm; FN, femoral neck; FX, fracture; HB, heel bone mineral density; LGS, low hand grip strength; LS, lumbar spine; WSs, walking speeds.

In analysis A, we adhered to a stringent significance threshold of *p*  < 5 × 10^−8^, which resulted in the inclusion of 288 SNPs across 51 cytokines (Supporting Information 1: Table S1). Within this framework, 16 cytokines were identified as exerting causal effects on osteoporosis traits (Figure 2A,B, Supporting Information 1: Table S2). Notably, five cytokines were associated with more than one trait: FGF19 (fibroblast growth factor 19, FA: beta = 0.205, 95% CI: 0.056–0.354, *p*=0.007; LS: Beta = 0.187, 95% CI: 0.105–0.269, *p*  < 0.001), FGF21 (FA: Beta = −0.176, 95% CI: −0.305 to −0.047, *p*=0.007; FN: Beta = −0.073, 95% CI: −0.136 to −0.010, *p*=0.023), IL10 (FN: Beta = −0.189, 95% CI: −0.067 to −0.022, *p*=0.013; LS: Beta = −0.112, 95% CI: −0.210 to −0.014, *p*=0.026), CXCL5 (C─X─C motif chemokine 5, FN: Beta = −0.038, 95% CI: −0.074 to −0.003, *p*=0.032; LS: Beta = −0.060, 95% CI: −0.101 to −0.019, *p*=0.004), and Oncostatin-M (LS: Beta = −0.113, 95% CI: −0.219 to −0.008, *p*=0.035; FX: OR = 1.163, 95% CI: 1.018–1.330, *p*=0.026). Furthermore, four cytokines with causal effects were identified in FA: TNFSF14 (tumor necrosis factor ligand superfamily member 14, Beta = 0.115, 95% CI: 0.006–0.223, *p*=0.038), CD40 (CD40L receptor, Beta = 0.089, 95% CI: 0.014–0.163, *p*=0.019), LTA (Beta = 0.056, 95% CI: 0.010–0.101, *p*=0.016), and CXCL6 (Beta = −0.086, 95% CI: −0.151 to −0.021, *p*=0.009). MCP3 (monocyte chemotactic protein-3, Beta = −0.080, 95% CI: −0.143 to −0.012, *p*=0.013) was causally associated with FN. CXCL11 (Beta = −0.086, 95% CI: −0.164 to −0.009, *p*=0.029) was causally associated with LS. CCL4 (C─C motif ligands 4, Beta = 0.024, 95% CI: 0.009–0.038, *p*=0.001), CXCL10 (Beta = 0.024, 95% CI: 0.002–0.045, *p*=0.032), DNER (delta and notch-like epidermal growth factor-related receptor, Beta = −0.022, 95% CI: −0.038 to −0.008, *p*=0.005), LIFR (leukemia inhibitory factor receptor, Beta = 0.088, 95% CI: 0.042–0.134, *p*  < 0.001), and T-cell surface glycoprotein CD5 (Beta = 0.039, 95% CI: 0.017–0.060, *p*  < 0.001) were causally associated with HBMD.

For the sarcopenia-related traits, a total of 13 cytokines with causal effects were identified (Figure 2C,D). Among them, LTA was causally associated with all three sarcopenia traits (ALM: Beta = 0.030, 95% CI: 0.003–0.058, *p*=0.032; LGS: OR = 0.963, 95% CI: 0.93–0.995, *p*=0.025; WP: Beta = 0.005, 95% CI: 0.001–0.009, *p*=0.006). While TNFSF12 (Beta = −0.049, 95% CI: −0.082 to −0.016, *p*=0.004), FGF5 (Beta = 0.012, 95% CI: 0.003–0.018, *p*=0.004), GDNF (Beta = −0.036, 95% CI: −0.067 to −0.006, *p*=0.019), DNER (Beta = 0.020, 95% CI: 0.004–0.035, *p*=0.013), CXCL6 (Beta = 0.007, 95% CI: 0.001–0.015, *p*=0.048), and IL10RB (Interleukin-10 receptor subunit beta, Beta = −0.014, 95% CI: −0.023 to −0.005, *p*=0.002) were identified to relate to ALM causally. However, outliers were identified in the analysis of TNFSF12 and LTA through MR-PRESSO. After correction, only the result of LTA was still significant. MCP2 (OR = 0.978, 95% CI: 0.959–0.998, *p*=0.033) and T-cell surface glycoprotein CD6 isoform (OR = 1.034, 95% CI: 1.006–1.062, *p*=0.017) were causally associated with LGS. CD40 (Beta = 0.009, 95% CI: 0.002–0.016, *p*=0.009), CXCL10 (Beta = −0.014, 95% CI: −0.024 to −0.004, *p*=0.004), FLT3L (FMS-related tyrosine kinase 3 ligand, Beta = −0.008, 95% CI: −0.016 to −0.001, *p*=0.047), CX3CL1 (Fractalkine, Beta = 0.018, 95% CI: 0.002–0.035, *p*=0.029), and SLAM (signaling lymphocytic activation molecule, Beta = −0.012, 95% CI: −0.022 to −0.001, *p*=0.030) were causally associated with WP.

In the conducted analysis A, the *F*-statistics for all utilized SNPs were deemed adequate for MR analysis, with values exceeding the threshold of 10 (Supporting Information 1: Table S1). Sensitivity analyses indicated an absence of directional pleiotropy. However, heterogeneity was observed in the analyses concerning HB involving CCL4 and LIFR, as well as ALM associated with GDNF and LTA. Furthermore, the majority of the results from four alternative methods demonstrated directional consistency with the IVW method. Notably, only one method exhibited directional inconsistency in comparison to the IVW method across seven analyses (Supporting Information 2: Figure S1).

**Figure 3:**
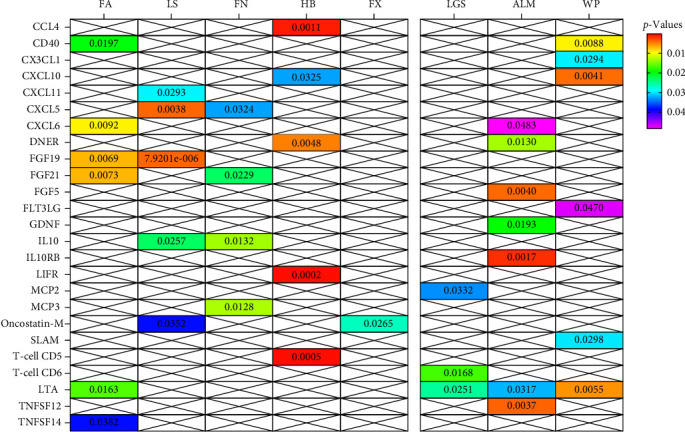
Heatmap of the causal associations of cytokines with osteosarcopenia traits in main analysis A. ALM, appendicular lean mass; FA, forearm; FN, femoral neck; FX, fracture; HB, heel bone mineral density; LGS, low hand grip strength; LS, lumbar spine; WSs, walking speeds.

In summary, results of analysis A revealed that five cytokines exert causal effects on osteosarcopenia traits (Figure 3). Among these, LTA stands out as it served as a protective factor not only for three distinct sarcopenia traits but also for FA BMD. Additionally, the CD40 demonstrated positive effects on both FA BMD and WS. Conversely, the results also showcased some inconsistencies in cytokine effects across different traits. For instance, CXCL6, while associated with lower FA BMD, had a beneficial impact on ALM. In a similar vein, CXCL10 was protective for HB, yet it increased the risk associated with usual WSs. Moreover, DNER exhibited a dual role by being a risk factor for HB, but protective for WS.

### 3.2. Main Analysis B

**Figure 4:**
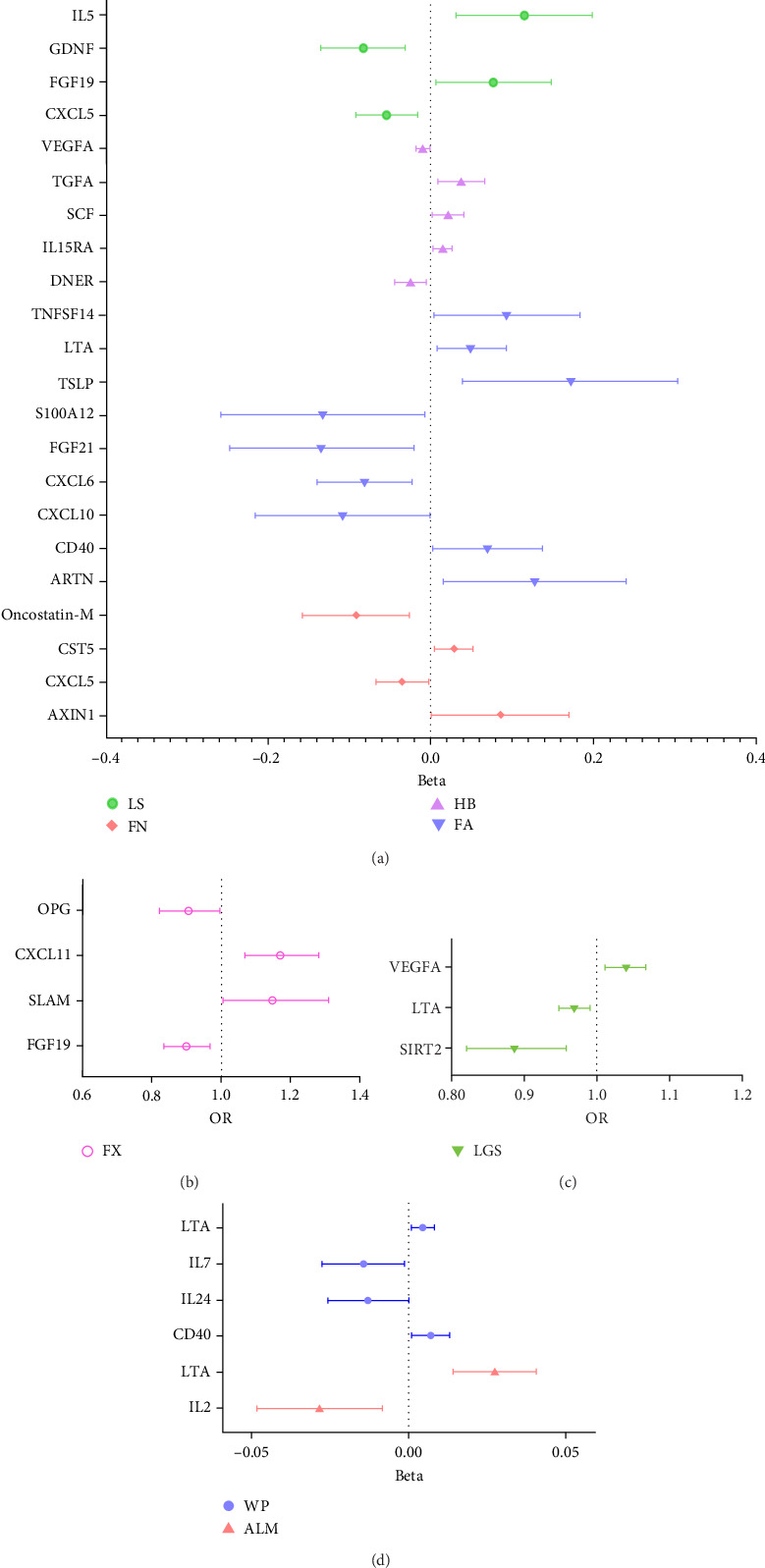
Forest plot results for main analysis B. (A, B) The results of osteoporosis traits; (C, D) the results of sarcopenia traits. ALM, appendicular lean mass; FA, forearm; FN, femoral neck; FX, fracture; HB, heel bone mineral density; LGS, low hand grip strength; LS, lumbar spine; WSs, walking speeds.

In analysis B, a less stringent statistical significance threshold of *p*  < 5 × 10^−6^ was applied, which allowed for the inclusion of 1800 SNPs (Supporting Information 1: Table S3). This analysis identified an expanded set of cytokines with causal effects on traits related to osteoporosis and sarcopenia, compared to main analysis A. The analysis encountered issues including heterogeneity, horizontal pleiotropy, and the presence of outliers. For osteoporosis traits, 26 cytokines with causal effects were identified (Figure 4A,B, Supporting Information 1: Table S4). Notable among these, three cytokines were linked to multiple traits: FGF19 (LS: Beta = 0.077, 95% CI: 0.006–0.149, *p*=0.033; FX: OR = 0.899, 95% CI: 0.837–0.968, *p*=0.004), CXCL5 (FN: Beta = −0.035, 95% CI: −0.067 to −0.002, *p*=0.039; LS: Beta = −0.054, 95% CI: −0.092 to −0.016, *p*=0.006), and Oncostatin-M (FN: Beta = −0.091, 95% CI: −0.157 to −0.026, *p*=0.006; LS: Beta = −0.101, 95% CI: −0.169 to −0.033, *p*=0.004). Besides, artemin (Beta = 0.128, 95% CI: 0.016–0.249, *p*=0.025), TNFSF14 (Beta = 0.094, 95% CI: 0.004–0.183, *p*=0.040), CD40 (Beta = 0.070, 95% CI: 0.004–0.137, *p*=0.039), CXCL10 (C─X─C motif chemokine 10, Beta = −0.108, 95% CI: −0.215 to −0.001, *p*=0.049), S100A12 (Protein S100-A12, OR = 0.876, 95% CI: 0.773–0.993, *p*=0.039), FGF21 (Beta = −0.134, 95% CI: −0.247 to −0.021, *p*=0.020), LTA (lymphotoxin-alpha, also known as TNF-*β*, Beta = 0.050, 95% CI: 0.008–0.093, *p*=0.020), CXCL6 (Beta = −0.081, 95% CI: −0.139 to −0.023, *p*=0.006), and TSLP (thymic stromal lymphopoietin; Beta = 0.172, 95% CI: 0.040–0.303, *p*=0.012) were causally associated with FA. CST5 (Cystatin D, Beta = 0.029, 95% CI: 0.005–0.052, *p*=0.016) and Axin-1 (Beta = 0.085, 95% CI: 0.001–0.169, *p*=0.047) were causally associated with FN. GDNF (glial cell line-derived neurotrophic factor; Beta = −0.083, 95% CI: −0.134 to −0.031, *p*=0.002), IL5 (Beta = 0.115, 95% CI: 0.031–0.198, *p*=0.007), TRAIL (TNF-related apoptosis-inducing ligand; Beta = −0.054, 95% CI: −0.099 to −0.009, *p*=0.018), and LIFR (Beta = 0.069, 95% CI: 0.002–0.135, *p*=0.043) were causally associated with LS. CCL4 (C─C motif ligands 4, Beta = 0.015, 95% CI: 0.001–0.030, *p*=0.049), DNER (Beta = −0.025, 95% CI: −0.045 to −0.005, *p*=0.013), IL15RA (interleukin 15 receptor subunit alpha, Beta = 0.014, 95% CI: 0.003–0.026, *p*=0.016), LIFR (Beta = 0.046, 95% CI: 0.014–0.079, *p*=0.005), SCF (stem cell factor, Beta = 0.021, 95% CI: 0.002–0.040, *p*=0.033), TGFA (Transforming growth factor alpha, Beta = 0.037, 95% CI: 0.008–0.066, *p*=0.011), VEGFA (vascular endothelial growth factor A) (TGFA, Beta = −0.010, 95% CI: −0.019 to −0.002, *p*=0.021) were causally associated with HB. However, outliers were identified in the majority analysis except IL15RA and VEGFA through MR-PRESSO. After correction, the *p*-values for CCL4 and LIFR were not significant, whereas the results of the other analyses were still significant (Supporting Information 1: Table S4). SLAM (OR = 1.149, 95% CI: 1.006–1.311, *p*=0.040), CXCL11 (C─X─C motif chemokine 11; OR = 1.171, 95% CI: 1.070–1.282, *p*=0.001), and OPG (osteoprotegerin, OR = 0.906, 95% CI: 0.823–0.997, *p*=0.044) were causally associated with FX.

For sarcopenia traits, a total of nine cytokines with causal effects were identified (Figure 4C,D). Among them, LTA was causally associated with all three sarcopenia traits (ALM: Beta = 0.028, 95% CI: 0.014–0.041, *p*=5.36e − 05; LGS: OR = 0.969, 95% CI: 0.948– 0.991, *p*=0.005; WP: Beta = 0.004, 95% CI: 0.001–0.008, *p*=0.017). While TNFSF12 (Beta = −0.036, 95% CI: −0.061 to −0.012, *p*=0.004), IL2 (Beta = −0.023, 95% CI: −0.049 to −0.009, *p*=0.005), GDNF (Beta = −0.023, 95% CI:−0.043 to −0.004, *p*=0.021), HGF (hepatocyte growth factor; Beta = −0.055, 95% CI:−0.102 to −0.008, *p*=0.021), IL-1*α* (Interleukin-1-alpha, Beta = 0.025, 95% CI: 0.001–0.049, *p*=0.040), and IL24 (OR = −0.034, 95% CI: −0.068 to −0.001, *p*=0.049) were causally associated with ALM. However, outliers were identified in the majority analysis except IL2 through MR-PRESSO (Supporting Information 1: Table S4). After correction, only the result of LTA was still significant. SIR2-like protein 2 (OR = 0.887, 95% CI: 0.822 –0.958, *p*=0.002) and VEGFA (OR = 1.040, 95% CI: 1.012–1.069, *p*=0.004) were causally associated with LGS. IL7 (Beta = −0.015, 95% CI: −0.028 to −0.001, *p*=0.005), CD40 (Beta = 0.007, 95% CI: 0.001–0.013, *p*=0.024), LTA (Beta = 0.004, 95% CI: 0.001–0.008, *p*=0.017), IL24 (Beta = −0.013, 95% CI: −0.026 to −0.001, *p*=0.049), and SULT1A1 (Sulfotransferase Family 1A Member 1, Beta = 0.011, 95% CI: 0.001–0.021, *p*=0.033) were causally associated with WP. While the result of SULT1A1 was not significant after being corrected through MR-PRESSO.

All *F* statistics of SNPs adopted in analysis B were strong enough for MR analysis (>10) (Supporting Information 1: Table S3). In the sensitivity analyses, directional pleiotropy was detected in the analysis of FN (Oncostatin-M), HB (IL15RA), and ALM (IL2 and LTA). While the heterogeneity was found in the analyses ALM (IL2 and LTA). Concerning the remaining four MR methodologies, the results were predominantly consistent with those obtained using the IVW method. However, discrepancies were noted in one method, which diverged from the IVW results in 11 separate analyses (Supporting Information 1: Table S4 and Supporting Information 3: Figure S2).

**Figure 5:**
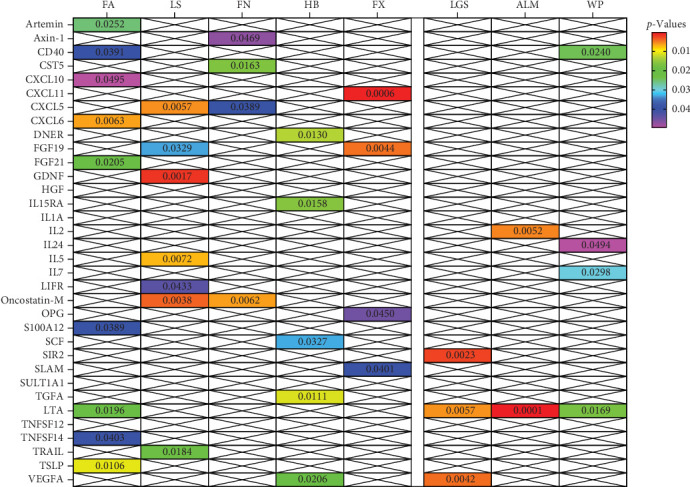
Heatmap of the causal associations of cytokines with osteosarcopenia traits in main analysis B. ALM, appendicular lean mass; FA, forearm; FN, femoral neck; FX, fracture; HB, heel bone mineral density; LGS, low hand grip strength; LS, lumbar spine; WSs, walking speeds.

Compared to analysis A, three cytokines with causal effects on both osteoporosis and sarcopenia were identified in analysis B (Figure 5). Among them, the results of LTA and CD40 were consistent. Besides, VEGFA was proven to a risk factor for both hand grip strength and HB.

### 3.3. Single-Cell Sequencing Reveals Cellular Origin of Casual Cytokines

**Figure 6:**
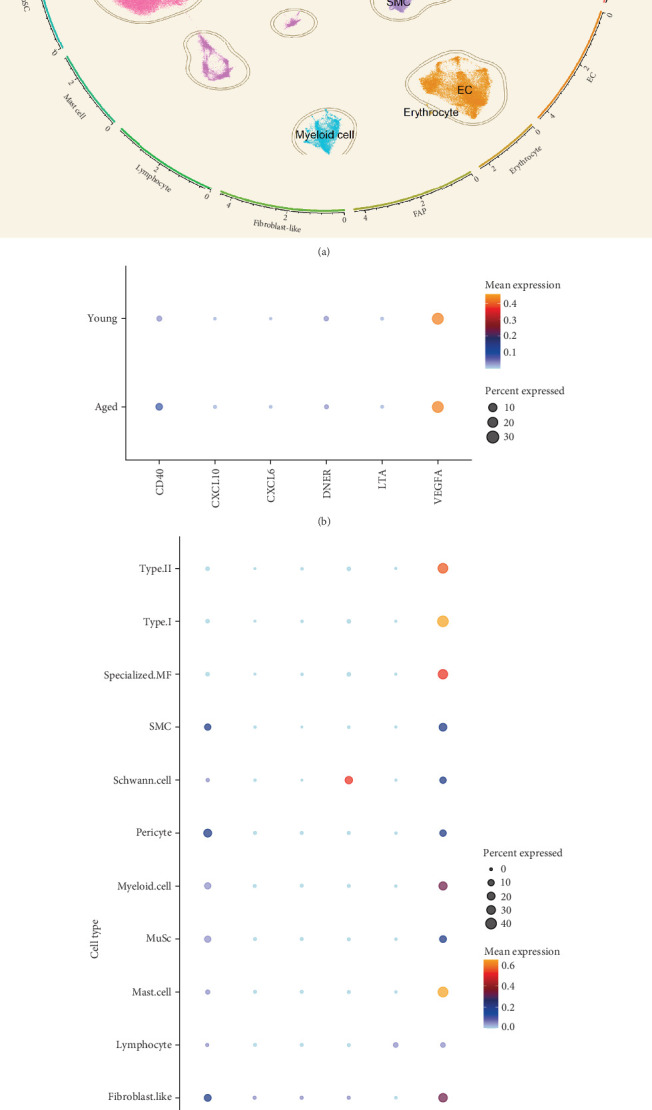
Single cell analysis of human skeletal muscle. (A) Uniform manifold approximation and projection (UMAP) dimensionality reduction clustering identifies multiple cell subpopulations within human skeletal muscle. (B) Distribution of candidate cytokines across cell types in human skeletal muscle. (C) The group analysis of candidate cytokines expression in human skeletal muscle. ECs, endothelial cells; FAPs, fibro-adipogenic progenitors; MuSCs, muscle stem cells; specialized MF, specialized myofibres; SMCs, smooth muscle cells; Type I, type I myofibres; type II, type II myofibres.

Analyses A and B identified six cytokines that exert a causal effect on both osteoporosis and sarcopenia traits (Table 2). Next, we aimed to verify the expression profile of each candidate cytokine at single-cell resolution. For the sarcopenia, 15 main skeletal muscle cell types were identified (Figure 6A). The uniform manifold approximation and projection (UMAP) plot revealed that skeletal muscle consisted mainly of large multinucleated muscle fibers (type I, type II, and specialized myonuclei) and mononuclear cells (MuSCs, stromal cells [FAPs, fibroblast-like cells, and adipocytes], vascular cells [pericytes, smooth muscle cells (SMCs), and endothelial cells (ECs)], immune cells [myeloid cells, lymphocytes, and mast cells], and glial cells [Schwann cells]). Among the six causal cytokines for osteosarcopenia, LTA was highly expressed in the lymphocyte, DNER was highly expressed in glial cells (Schwann cells), while CXCL6 and CXCL10 were insignificantly expressed in all cell types. CD40 was highly expressed across the mononucleated cells, and VEGFA was highly expressed in all cell types with the exception of erythrocyte and lymphocyte (Figure 6B). The group analysis revealed that the expression of LTA, VEGFA, and CXCL6 were consistent with MR results, with higher expression of LTA in the young group compared to the aged group, while VEGFA expression was higher in the aged group compared to the young group, and higher expression of CXCL6 in mast cells of the young group. While the total expressions for DNER and CD40 were opposite to the MR results, with higher gene expression levels in the aged group (Figure 6C). However, the expression of CD40 in myeloid cells was higher in the young group compared to the aged group (Supporting Information 4: Figure 3A).

**Figure 7:**
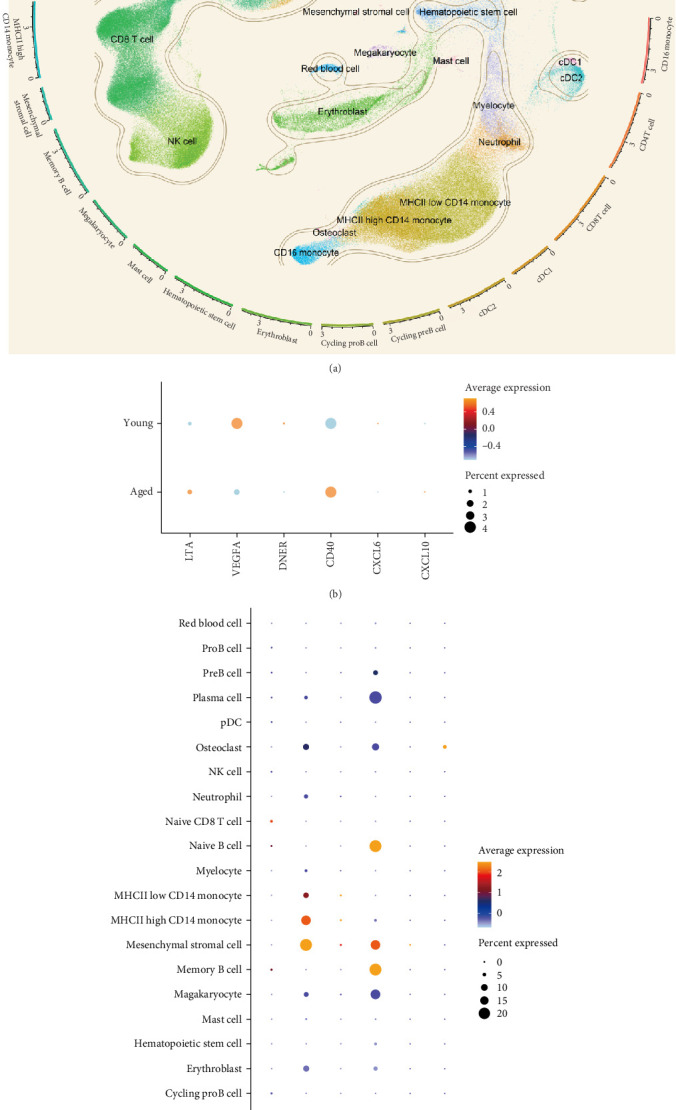
Single cell analysis of human bone marrow. (A) Uniform manifold approximation and projection (UMAP) dimensionality reduction clustering identifies multiple cell subpopulations within human bone marrow. (B) Distribution of candidate cytokines across cell types in human bone marrow. (C) The group analysis of candidate cytokines expression in human bone marrow.

In the analysis of scRNA sequencing data from healthy BM, 26 distinct cell types were identified (Figure 7A). These were primarily categorized into five major groups: T/NK cells, B cells, monocytes/dendritic cells (DCs), progenitor cells, and erythrocytes/megakaryocytes. Additionally, smaller clusters of osteoclasts, plasma cells, and mesenchymal stromal cells (MSCs) were detected. Among the six candidate cytokines for osteosarcopenia, DNER and CXCL6 were insignificantly expressed in all cell types. While LTA was highly expressed in lymphocytes, DNER was highly expressed in osteoclasts, VEGFA was highly in DCs, MSCs, and osteoclasts, and CD40 was highly expressed in B cells, megakaryocytes, MSCs, and plasma cells (Figure 7B). In the group analysis, the overall difference in expression of CXCL10 was consistent with MR results, while the rest results were opposite to the MR results (Figure 7C). However, in certain specific cell types, the results were consistent with MR analysis. Specifically, the expression of LTA in CD8 and CD4 T cells was higher in the young group compared to the aged group, and VEGFA in cDC2 was higher in the aged group compared to the young group (Supporting Information 4: Figure 3B).

## 4. Discussions

Despite increasing awareness of osteosarcopenia, our understanding of its pathophysiological mechanisms remains limited, resulting in a lack of effective pharmacological treatments. The shared pathophysiological features of osteoporosis and sarcopenia could facilitate the identification of potential pharmacological targets for osteosarcopenia treatment. Our study, through MR analysis, provided preliminary insights into the role of inflammation in osteosarcopenia development, suggesting that certain cytokines may have a potentially important impact. Furthermore, the cell-specific cytokine expression revealed by scRNA analysis would significantly enhance the design of experimental studies that utilize tissue- and cell-specific gene KOs for functional tracking, thus identifying novel therapeutic targets for osteosarcopenia treatment.

Previous studies have utilized MR to investigate the casual role of cytokines on osteoporosis and sarcopenia separately, yielding important insights into the genetic and molecular underpinnings of these conditions [20, 22, 36]. While in the current study, we expanded on previous research by incorporating new data on 91 cytokines with a larger sample size [26]. However, given the lack of a dedicated osteosarcopenia database, we relied on data associated with osteoporosis and sarcopenia separately, potentially limiting direct application to this combined phenotype. Within these constraints, this comprehensive approach allowed us to identify six cytokines—LTA, CD40, CXCL6, CXCL10, DNER, and VEGFA—that may be involved in osteosarcopenia traits.

Among them, LTA emerged as a particularly significant cytokine, serving as a protective element for both sarcopenia traits and FA BMD. LTA is a cytokine belonging to the TNF family [37]. Primarily secreted by activated T cells and B cells, it plays a crucial role in immune responses and inflammation. Compared to TNF-*α*, another well-known cytokine in the TNF family, the role of LTA in bone and muscle has been less extensively investigated. Previous research has suggested that LTA can bind TNF receptors (TNFR1 and TNFR2) and induce osteoclastogenesis alongside RANKL [38]. However, direct evidence of LTA specifically inducing osteoclastogenesis is limited. In contrast, pateclizumab, a specific blocker of LTA, showed much-reduced efficacy compared to the TNF blocker adalimumab in a clinical trial for rheumatoid arthritis, which may call into question the risky role of LTA in bone [39]. Furthermore, aligning with our results, several clinical studies have demonstrated that LTA may have played a protective role in osteoporosis. For instance, Kim et al. [40] found that serum LTA levels were lower in osteoporotic postmenopausal women compared to normal postmenopausal women, and these levels increased after estrogen plus progestogen therapy. More recently, Cedeno-Veloz et al. [41] also found that LTA exhibited a negative correlation with FX risk. Simultaneously, Ribeiro et al. [42] indicated that serum LTA levels were higher in participants with normal muscle strength compared to dynapenic participants. However, as a proinflammatory cytokine, the precise mechanisms of LTA in musculoskeletal health warrant further investigation, given the limited experimental and clinical evidence available. The multifaceted nature of LTA's role underscores the need for further research to fully understand its mechanisms and therapeutic potential in osteosarcopenia.

CD40, through its interaction with CD40L, regulates immune responses and inflammation and has a dual role in bone metabolism, as observed in various clinical and experimental settings. For instance, CD40 knockout (KO) and CD40L KO mice, as well as T-cell-deficient nude mice, exhibited increased bone loss, enhanced bone resorption, and reduced OPG production [43]. Additionally, activated CD40L^−/−^ T cells from both humans and mice promoted osteoclast differentiation of monocytes due to IFN-*γ* deficiency. However, studies have also shown that mice lacking CD40L in T cells were resistant to ovariectomy-induced bone loss [44]. The methylation of CpG sites in the CD40 promoter influenced BMD acquisition, and downregulation of OPG is linked to lower BMD in TT women with the rs1883832 variant of the CD40 gene [45]. In line with our results, another MR study by Cao et al. [46] also supported the protective role of CD40 on BMD, showing a positive correlation between CD40 and BMD at four sites. The mechanisms underlying the associations between bone and CD40 are not yet fully understood, necessitating further functional studies to elucidate this relationship. Significantly less research has been conducted on the role of CD40 in skeletal muscle. An interesting study revealed that CD40/CD40L interactions play an important role in intestinal muscle hypercontraction, supporting our findings of CD40^‘^s protective role on WS [47]. However, increased serum levels of CD40L have been observed in various muscle diseases, including hereditary muscular dystrophies, immune-mediated necrotizing myopathy, and sporadic inclusion body myositis, although these studies had limited sample sizes [48]. Our findings suggested a potential role for the CD40/CD40L system in the development of osteosarcopenia, highlighting the need for further research to explore and confirm its mechanistic involvement.

Contrary to the majority of the literature, our findings also indicate that VEGFA is a risk factor for both hand grip strength and HB. VEGFA is widely recognized for its role in promoting angiogenesis, which are critical for musculoskeletal health and repair [49]. VEGFA's role in bone metabolism has been predominantly viewed as beneficial. For instance, Hu and Olsen [50] highlighted the crucial role of VEGFA in bone development and homeostasis, emphasizing its importance in maintaining bone vascularity and promoting osteogenesis. Similarly, Buettmann et al. [51] demonstrated the necessity of VEGFA from early osteoblast lineage cells for effective FX healing in mice. These studies underscored VEGFA's positive impact on bone formation and repair processes. However, our results aligned more closely with emerging evidence suggesting a dual role for VEGFA in bone health. Xu et al. [52] found that increased VEGFA expression can promote apoptosis of osteoblasts, thereby accelerating osteoporosis. Additionally, VEGFA plays a crucial role in muscle tissue by promoting angiogenesis and aiding muscle regeneration, enhancing blood flow, and facilitating recovery from damage. Despite these beneficial roles, VEGFA was also upregulated in several pathological disorders, including a range of cardiovascular diseases and tumors. Recently, St. Sauver et al. [53] highlighted VEGFA as a significant biomarker associated with increased mortality risk. In conjunction with our findings, these studies suggested that the role of VEGFA in musculoskeletal metabolism is complex and warrants further investigation to fully elucidate the conditions under which it may contribute to bone deterioration and muscle weakness.

In addition to the cytokines mentioned above, we also identified three cytokines that had opposite effects on bone and muscle: CXCL6, CXCL10, and DNER. CXCL6 and CXCL10 both belong to CXCL chemokine family, which primarily attract the neutrophils and T cells to sites of inflammation, infection, and trauma, respectively. DNER functions through the Notch signaling pathway, though this role remains controversial. These cytokines were primarily studied for their roles in immune response and inflammation, their impact on musculoskeletal health warrants further investigation. These findings underscored that inflammation may exert context-dependent and at times opposing effects on bone and muscle—an observation consistent with existing literature on certain cytokines. For instance, RANKL, a well-known target for osteoporosis, has recently been found to improve skeletal muscle function by inducing mitochondrial biogenesis [54]. Given the limited direct research on osteosarcopenia, further studies are warranted to clarify the net impact of these cytokines on both tissues when they are concurrently affected.

The current study employed MR to leverage the statistical power obtained from large cohort GWAS, identifying candidate cytokines that may regulate the musculoskeletal system and potentially contribute to the development of osteosarcopenia. However, functional validation and characterization of additional candidate cytokines are still needed. Establishing the mechanistic role of each candidate cytokine in musculoskeletal metabolism is essential, as translational studies could then leverage these genetic determinants as therapeutic targets. To further this objective, we employed publicly available single-cell datasets to investigate cytokine expression at the single-cell level. Our analysis revealed that CD40 and VEGFA were highly expressed across various cell populations within muscle and BM, whereas other cytokines exhibited high expression levels only in specific cell types. Specifically, CD40 was highly expressed in monocytes within muscle and immune cells within BM. VEGFA demonstrated strong expression in multinucleated myofibers and adipocytes in muscle, as well as in monocytes and MSCs in BM. Furthermore, LTA showed high expression in lymphocytes within both muscle and BM. The expression patterns of these candidate cytokines suggest that they may not directly induce primary (intrinsic) musculoskeletal metabolic changes but rather trigger secondary (extrinsic or systemic) effects by modulating the surrounding inflammatory microenvironment. While most cytokine expression patterns aligned with MR findings, some discrepancies were observed—particularly in BM single-cell data—highlighting the complexity of interpreting results across datasets not specifically tailored to osteosarcopenia. These inconsistencies further emphasize that our findings remain exploratory and require direct functional validation in controlled experimental systems. Despite these discrepancies, our findings suggest that these candidate cytokines play key roles in the maintenance or degradation of bone tissue. These results provide a framework for testing the genes in cell-specific mouse models, where each gene can be knocked out based on its specific expression pattern. This approach will be crucial for designing functional validation experiments to investigate the mechanistic roles of each candidate cytokine and advance them as potential therapeutic targets.

This study has several limitations that must be acknowledged. First, although MR analysis provides a robust approach to inferring causality, it is still limited by the assumptions inherent in the method. One key assumption is the absence of pleiotropy, where genetic variants affect the outcome through pathways other than the exposure of interest. Despite our efforts to use sensitivity analyses, such as MR-Egger regression, to account for potential pleiotropy, the presence of unmeasured pleiotropy cannot be entirely ruled out, and this could still influence the results. Second, the study's findings were based on data from large cohort GWAS; however, although the sample size is larger than in previous studies, it may still be insufficient to detect smaller effect sizes or to generalize findings across diverse populations. Furthermore, the reliance on separate datasets for osteoporosis and sarcopenia—due to the absence of a large-scale osteosarcopenia-specific database—may limit the specificity of our conclusions regarding the combined osteosarcopenia phenotype. The majority of the data used in this study was derived from populations of European ancestry, which may reduce the applicability of our findings to other ethnic groups. Third, while the scRNA-seq analysis provided valuable insights into cell-specific cytokine expression, the resolution and depth of the single-cell data may vary depending on the dataset used. Discrepancies between the MR and scRNA-seq results could arise from technical variations in the scRNA-seq data, differences in the cell populations captured, or the dynamic nature of cytokine expression, which can change based on the tissue environment or experimental conditions. Moreover, potential measurement errors in both GWAS and scRNA-seq data could introduce biases, which should be considered when interpreting the results. Lastly, the identification of cytokines through MR analysis and scRNA-seq provides potential targets, but functional validation in experimental models is essential to confirm these findings.

## 5. Conclusions

In summary, this study bridged genetic and cellular mechanisms in osteosarcopenia, leveraging large cohort GWAS data and scRNA-seq. We identified candidate cytokines—LTA, CD40, CXCL6, CXCL10, DNER, and VEGFA—that were potentially crucial in regulating musculoskeletal health and may serve as potential therapeutic targets for osteosarcopenia. Single-cell sequencing provided detailed insights into their specific roles in different cell types. This integrative approach not only enhanced the understanding of osteosarcopenia but also facilitated the functional characterization of these candidate cytokines using cell-specific models. Overall, our findings pave the way for the development of targeted therapies to mitigate bone and muscle loss in the aging population, ultimately improving patient outcomes, while additional functional and clinical studies are still necessary to confirm their roles and therapeutic potential.

## Figures and Tables

**Table 1:** The sources for all statistical summary datasets used in this study.

Consortium	Phenotype	Sample size	PMID/source	GWAS ID	Population
—	Plasma protein	14,824	37563310	GCST90274758 to GCST90274848	European
MRC-IEU	Low hand grip strength	256,523	33510174	ebi-a-GCST90007526	European
UK Biobank	Appendicular lean mass	450,243	33097823	ebi-a-GCST90000025	European
MRC-IEU	Usual walking pace	459,915	30104761	ukb-b-4711	European
GEFOS	Forearm bone mineral density	8143	26367794	ieu-a-977	European
GEFOS	Lumbar spine bone mineral density	28,498	26367794	ieu-a-982	European
GEFOS	Femoral neck bone mineral density	32,735	26367794	ieu-a-980	European
GEFOS	Fracture	264,973	30158200	—	Predominant European
GEFOS	Heel bone mineral density	426,824	30598549	GCST006979	European

**Table 2:** Integrated cytokine analysis: MR and scRNA-seq findings.

	MR analysis		Single cell analysis	
	Osteoporosis traits	Sarcopenia traits	Bone marrow	Muscle
LTA^a,b^	FA (+)	GS, ALM, WS (+)	Lymphocytes	Lymphocytes
CD40^a,b^	FA (+)	WS (+)	B cells, megakaryocytes, MSCs, and plasma cells	Mononucleated cells
VEGFA^b^	HB (−)	GS (−)	Dendritic cells, mesenchymal stem cells, and osteoclasts	Highly expressed in all cell types except erythrocytes and lymphocytes
DNER^a^	HB (−)	WS (+)	Insignificantly expressed in all cell types overall	Schwann cells
CXCL6^a^	FA (−)	ALM (+)	Insignificantly expressed in all cell types overall	
CXCL10^a^	HB (+)	WS (−)	Insignificantly expressed in all cell types overall	

Abbreviations: ALM, appendicular lean mass; FA, forearm; FN, femoral neck; FX, fracture; GS, hand grip strength; HB, heel bone mineral density; LS, lumbar spine; WSs, walking speeds.

^a^Cytokines identified in analysis A.

^b^Cytokines identified in analysis B.

## Data Availability

The original contributions presented in the study are included in the article/supporting information, further inquiries can be directed to the corresponding authors.

## Supporting Information

Supporting Information 1 Table S1: SNPs adopted in analysis A. Table S2: MR results of main analysis A. Table S3: SNPs adopted in analysis B. Table S4: MR results of main analysis B.

Supporting Information 2 Figure S1: MR results of main analysis A. The figure for each analysis was arranged in the following order: (1) comparison of results using different MR methods; (2) leave-one-out sensitivity analysis; and (3) funnel plot of MR analysis.

Supporting Information 3 Figure S2: MR results of main analysis B. The figure for each analysis was arranged in the following order: (1) comparison of results using different MR methods; (2) leave-one-out sensitivity analysis; and (3) funnel plot of MR analysis.

Supporting Information 4 Figure S3: Expression of candidate cytokines in different cell types between subgroups. (A) Results of human skeletal muscle. (B) Results of human bone marrow.
