# Supplementary material for: Causal Associations of Inflammatory Cytokines With Osteosarcopenia: Insights From Mendelian Randomization and Single Cell Analysis
Source: Mediators Inflamm. 2025 Apr 3;2025:6005225. doi: 10.1155/mi/6005225 (PMC11986192; doi:10.1155/mi/6005225)
Supplement: Supporting Information 2 — Figure S1: MR results of main analysis A. The figure for each analysis was arranged in the following order: (1) comparison of results using different MR methods; (2) leave-one-out sensitivity analysis; and (3) funnel plot of MR analysis. [file 6005225.f2.pdf]

Figure S1:MR results of Main analysis A. The figure for each analysis were arranged in the following order: 1. Comparison of results using different MR methods; 2. Leave-one-out sensitivity analysis; 3. Funnel plot of MR analysis.

Results for FA

CD40

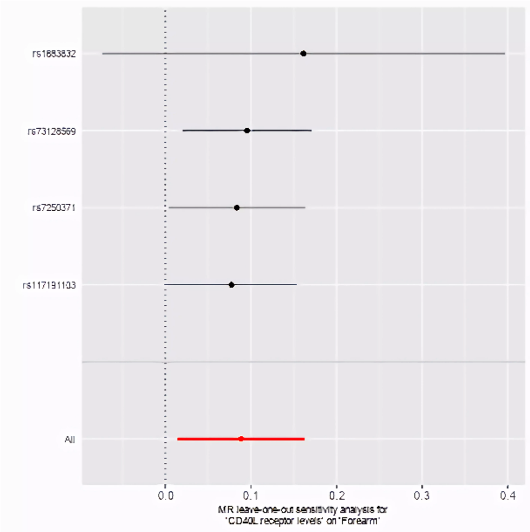

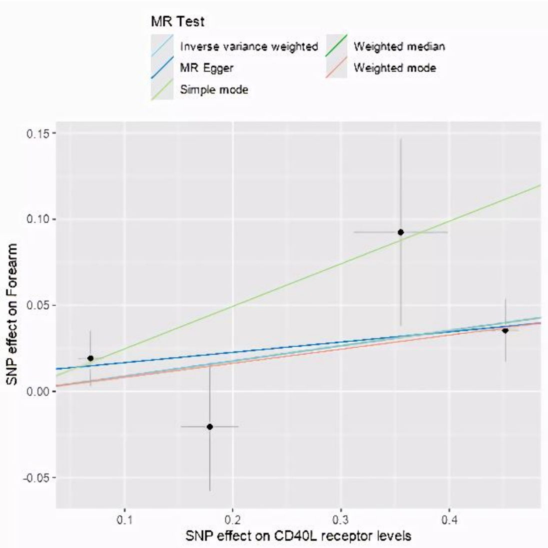

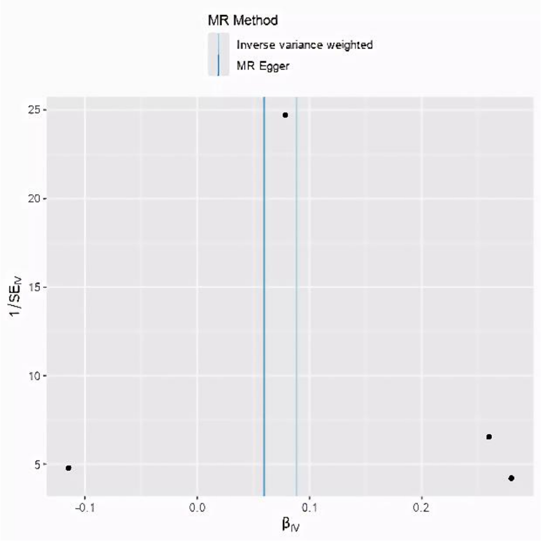

CXCL6

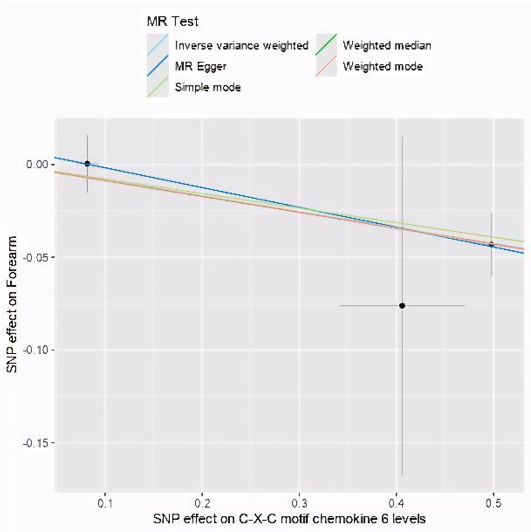
MR Test
Inverse variance weighted
MR Egger
Simple mode
Weighted median
Weighted mode
0.00
-0.05
-0.10
-0.15
SNP effect on Forearm
0.1
0.2
0.3
0.4
0.5
SNP effect on C-X-C motif chemokine 6 levels

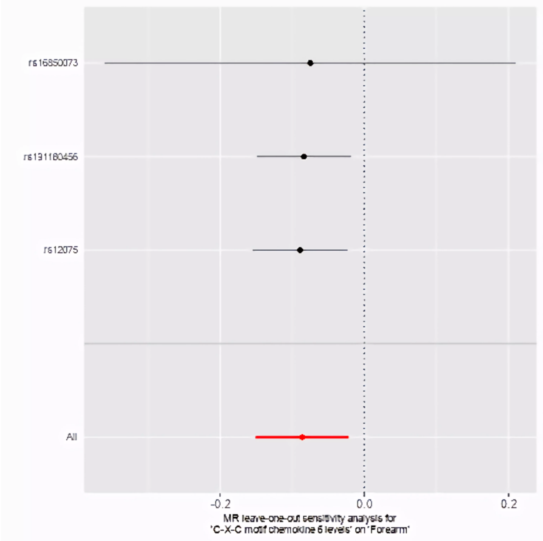
rs16850073
rs131180456
rs12075
All
-0.2
0.0
0.2
MR leave-one-out sensitivity analysis for
'C-X-C motif chemokine 6 levels' on 'Forearm'

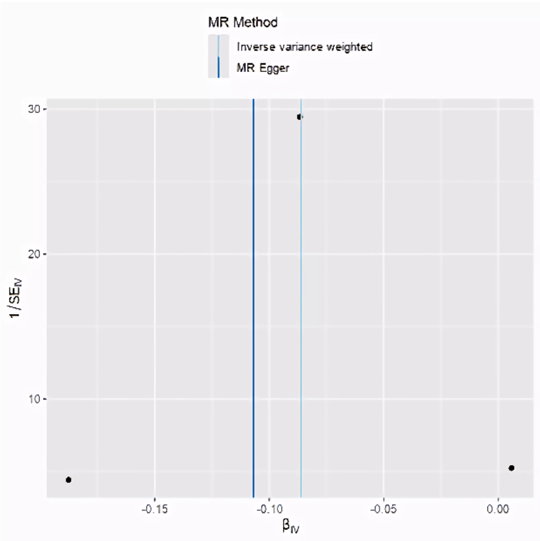
MR Method
Inverse variance weighted
MR Egger
30
20
10
1/SE$_{IV}$
-0.15
-0.10
-0.05
0.00
β$_{IV}$

FGF19

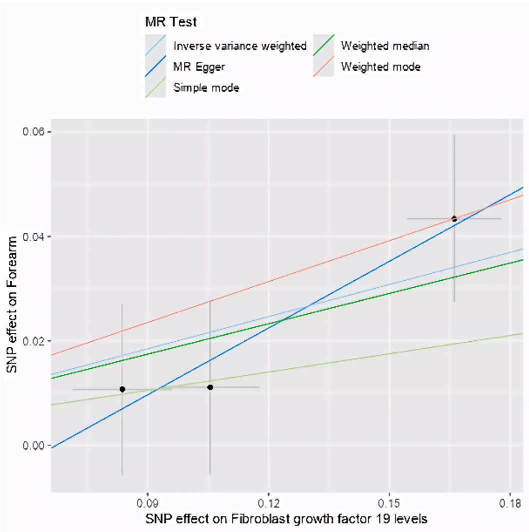
MR Test
Inverse variance weighted
MR Egger
Simple mode
Weighted median
Weighted mode
0.06
0.04
0.02
0.00
SNP effect on Forearm
0.09
0.12
0.15
0.18
SNP effect on Fibroblast growth factor 19 levels

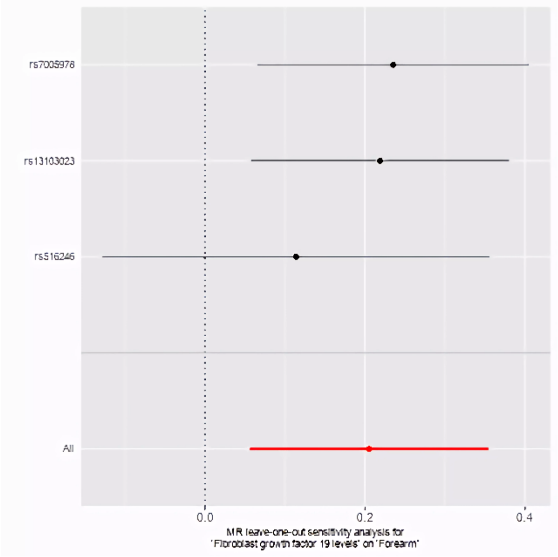
rs7005978
rs13103023
rs516246
All
0.0
0.2
0.4
MR leave-one-out sensitivity analysis for
'Fibroblast growth factor 19 levels' on 'Forearm'

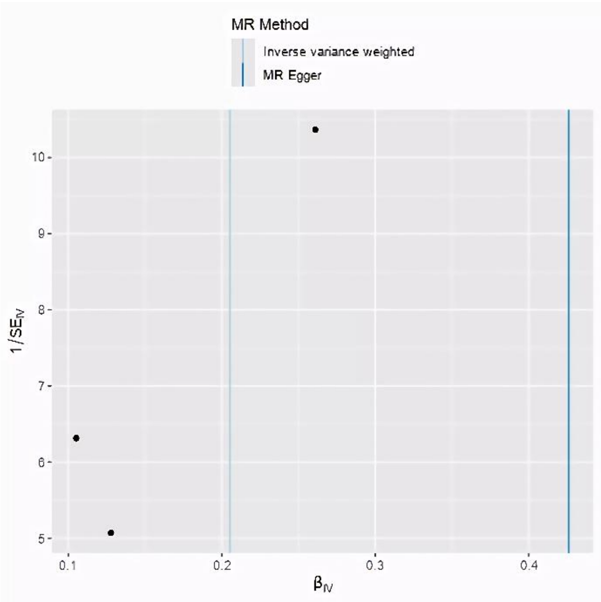
MR Method
Inverse variance weighted
MR Egger
10
9
8
7
6
5
1/SE$_{IV}$
0.1
0.2
0.3
0.4
β$_{IV}$

FGF21

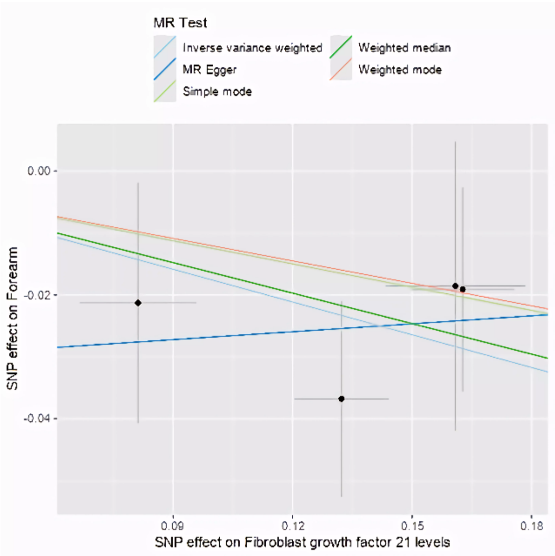
MR Test
Inverse variance weighted
MR Egger
Simple mode
Weighted median
Weighted mode
0.00
-0.02
-0.04
SNP effect on Forearm
0.09
0.12
0.15
0.18
SNP effect on Fibroblast growth factor 21 levels

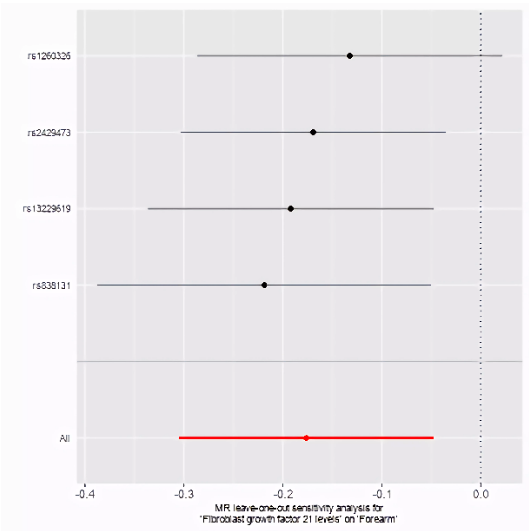
rs1260326
rs2429473
rs13229619
rs838131
All
-0.4
-0.3
-0.2
-0.1
0.0
MR leave-one-out sensitivity analysis for
'Fibroblast growth factor 21 levels' on 'Forearm'

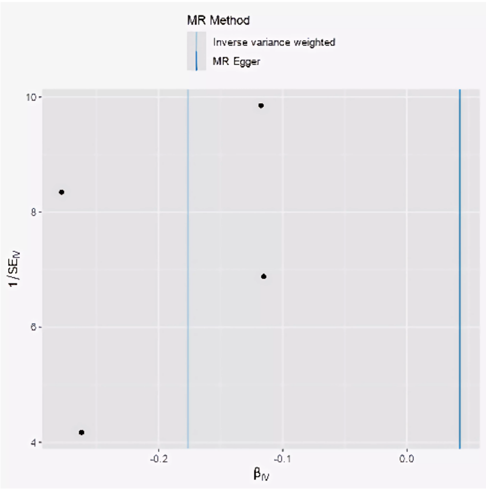
MR Method
Inverse variance weighted
MR Egger
10
8
6
4
1/SE$_{IV}$
-0.2
-0.1
0.0
β$_{IV}$

LTA

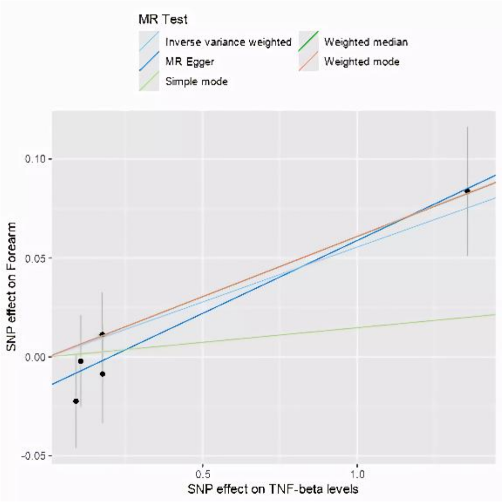
MR Test
Inverse variance weighted
MR Egger
Simple mode
Weighted median
Weighted mode
SNP effect on Forearm
0.10
0.05
0.00
-0.05
0.5
1.0
SNP effect on TNF-beta levels

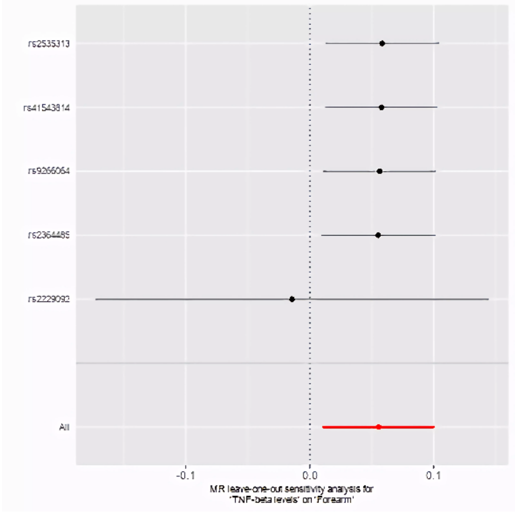
rs2535313
rs41543814
rs9266064
rs2364485
rs2229092
All
-0.1
0.0
0.1
MR leave-one-out sensitivity analysis for
'TNF-beta levels' on 'Forearm'

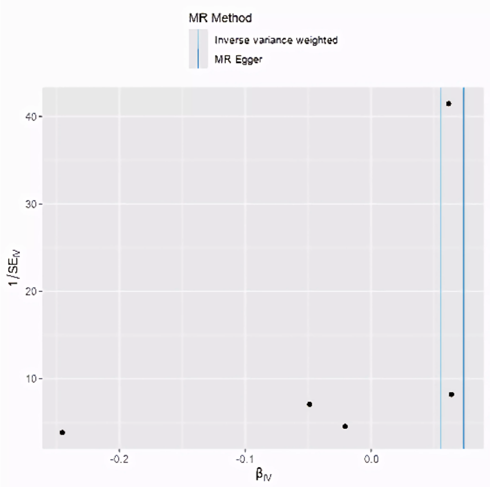
MR Method
Inverse variance weighted
MR Egger
40
30
20
10
$1/SE_{IV}$
-0.2
-0.1
0.0
β_{IV}

TNFSF14

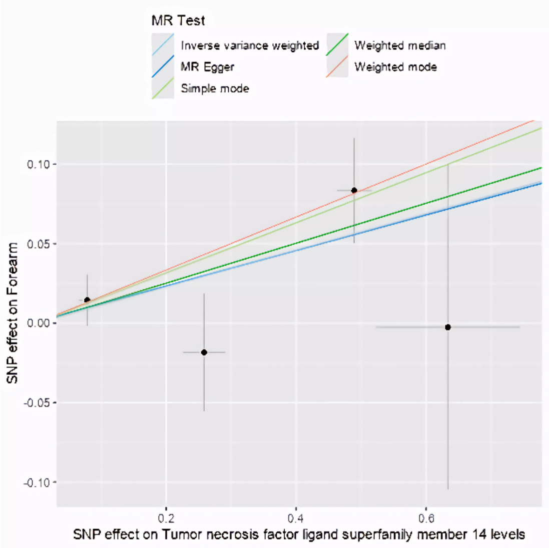
MR Test
Inverse variance weighted
MR Egger
Simple mode
Weighted median
Weighted mode
SNP effect on Forearm
0.10
0.05
0.00
-0.05
-0.10
0.2
0.4
0.6
SNP effect on Tumor necrosis factor ligand superfamily member 14 levels

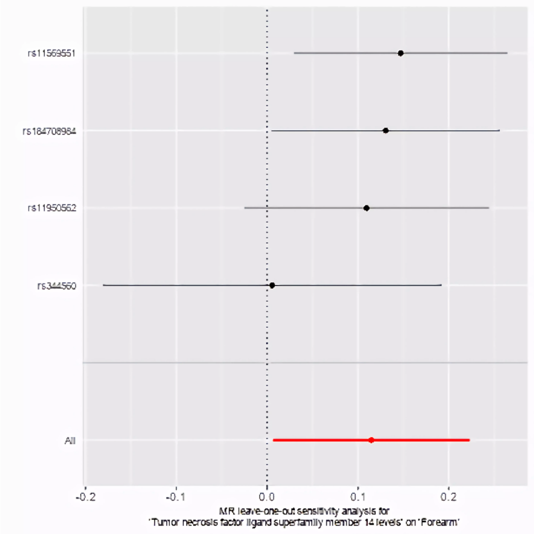
rs11569551
rs184708984
rs11950562
rs344560
All
-0.2
-0.1
0.0
0.1
0.2
MR leave-one-out sensitivity analysis for
'Tumor necrosis factor ligand superfamily member 14 levels' on 'Forearm'

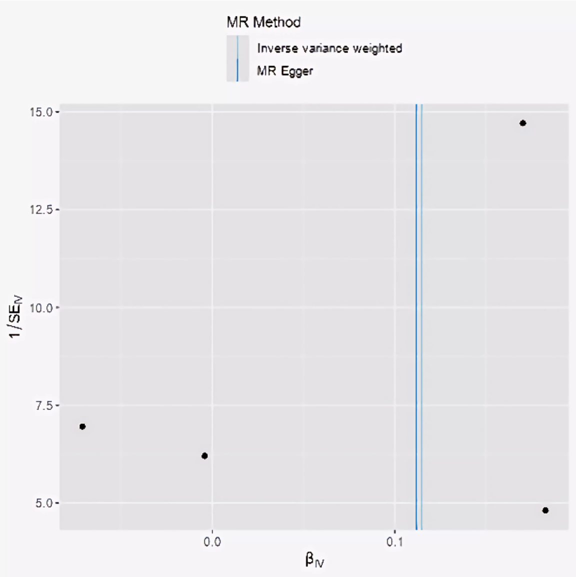

Results for FN

CXCL5

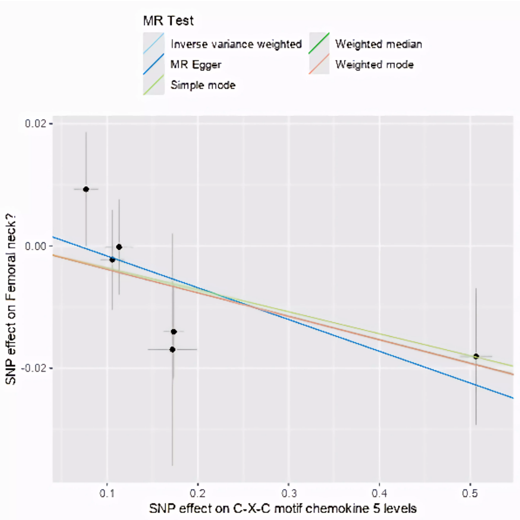

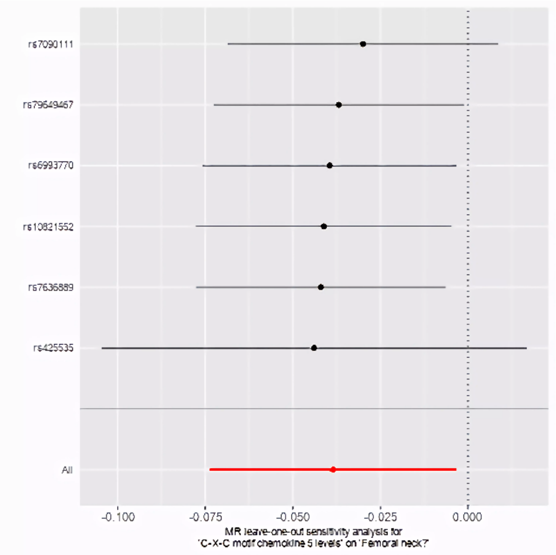

MCP3

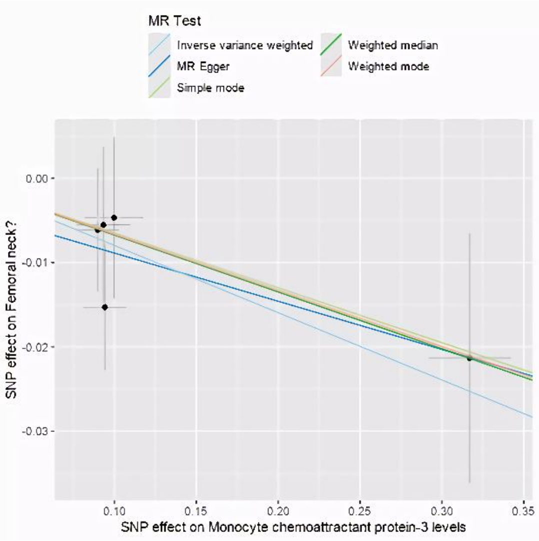

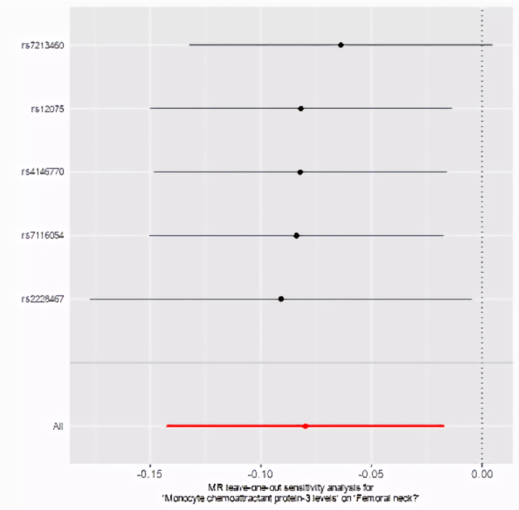

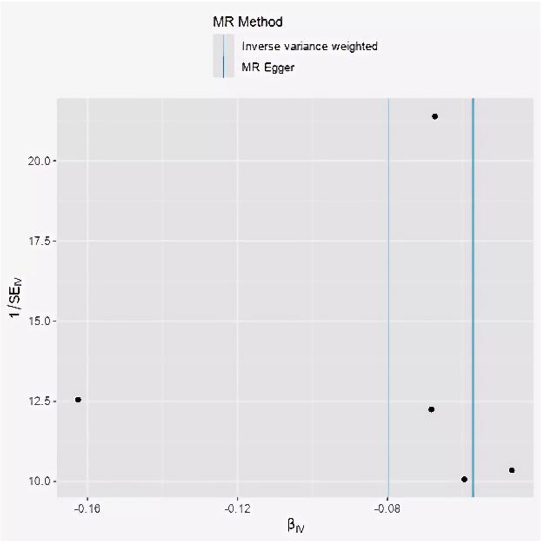
MR Method
Inverse variance weighted
MR Egger
20.0
17.5
15.0
12.5
10.0
-0.16
-0.12
-0.08
1/SE$_{IV}$
β$_{IV}$

FGF21

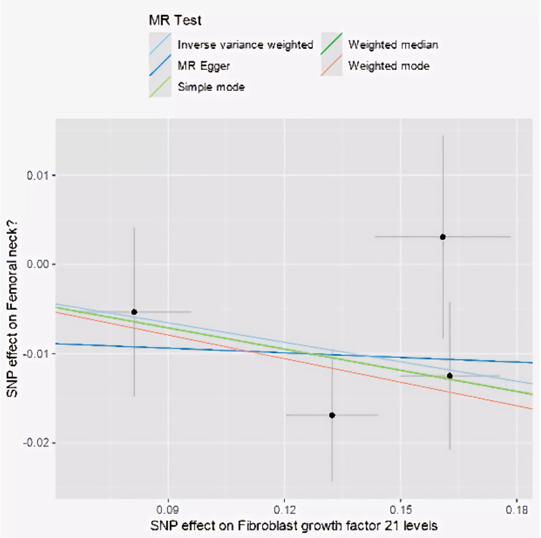
MR Test
Inverse variance weighted
MR Egger
Simple mode
Weighted median
Weighted mode
0.01
0.00
-0.01
-0.02
0.09
0.12
0.15
0.18
SNP effect on Femoral neck?
SNP effect on Fibroblast growth factor 21 levels

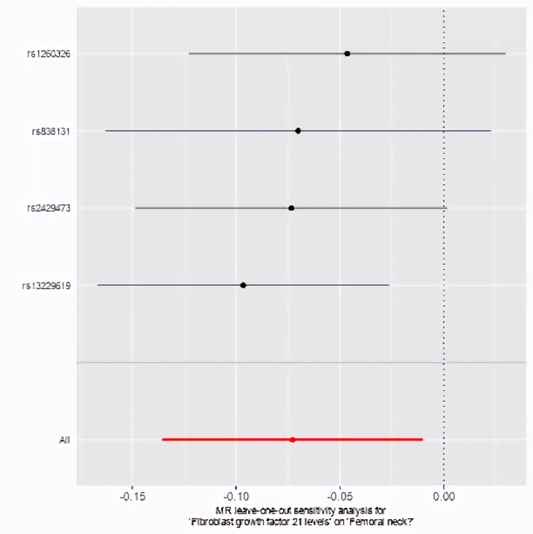
rs1260326
rs838131
rs2429473
rs13229619
All
-0.15
-0.10
-0.05
0.00
MR leave-one-out sensitivity analysis for
'Fibroblast growth factor 21 levels' on 'Femoral neck?'

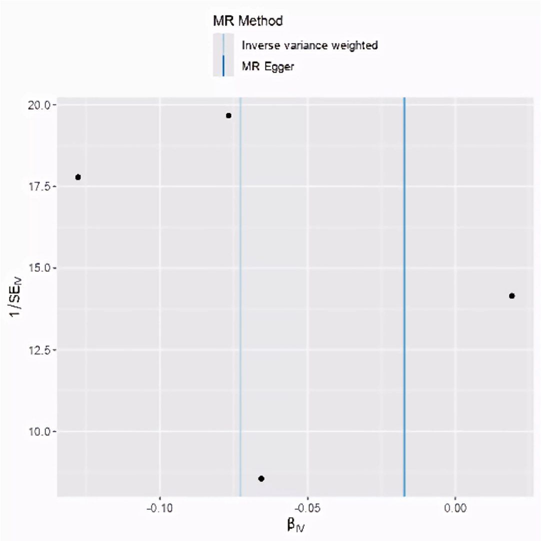
MR Method
Inverse variance weighted
MR Egger
20.0
17.5
15.0
12.5
10.0
-0.10
-0.05
0.00
1/SE$_{IV}$
β$_{IV}$

IL10

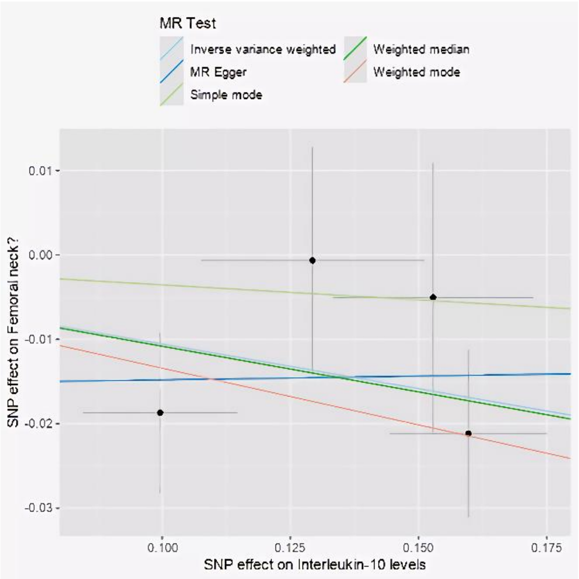

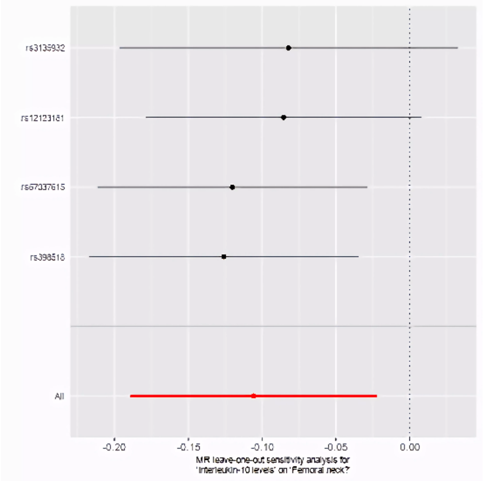

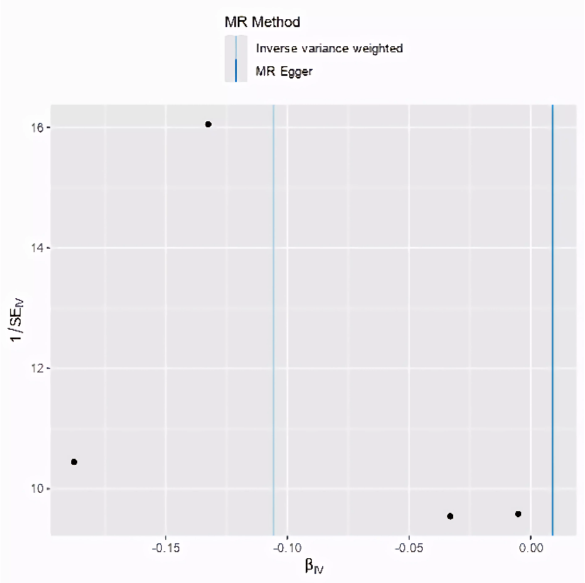

Results for LS

CXCL11

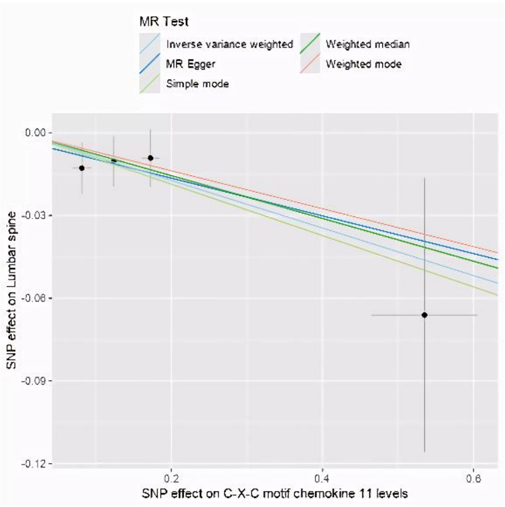

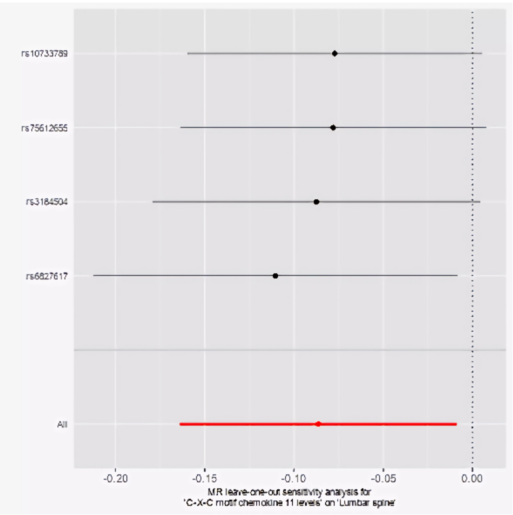

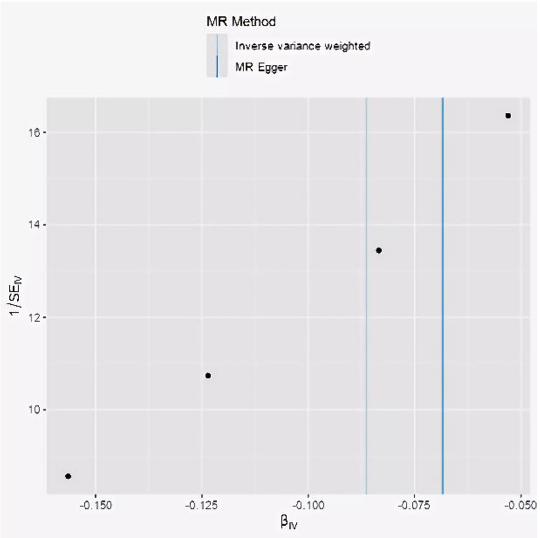
MR Method
Inverse variance weighted
MR Egger
16
14
12
10
1/SE$_{IV}$
-0.150
-0.125
-0.100
-0.075
-0.050
β$_{IV}$

CXCL5

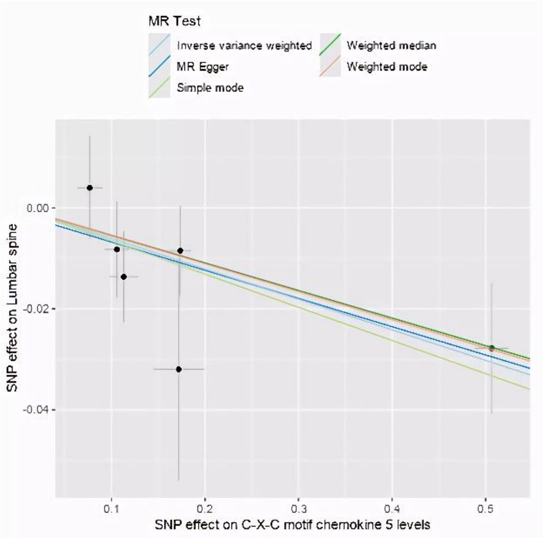
MR Test
Inverse variance weighted
MR Egger
Simple mode
Weighted median
Weighted mode
0.00
-0.02
-0.04
SNP effect on Lumbar spine
0.1
0.2
0.3
0.4
0.5
SNP effect on C-X-C motif chemokine 5 levels

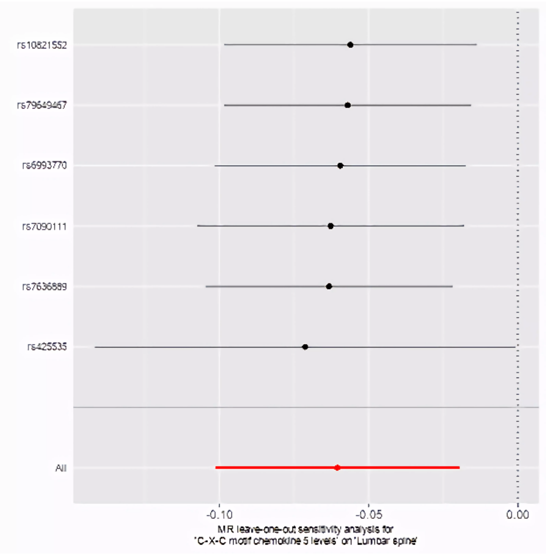
rs10821552
rs79649467
rs6993770
rs7090111
rs7636889
rs425535
All
-0.10
-0.05
0.00
MR leave-one-out sensitivity analysis for
'C-X-C motif chemokine 5 levels' on 'Lumbar spine'

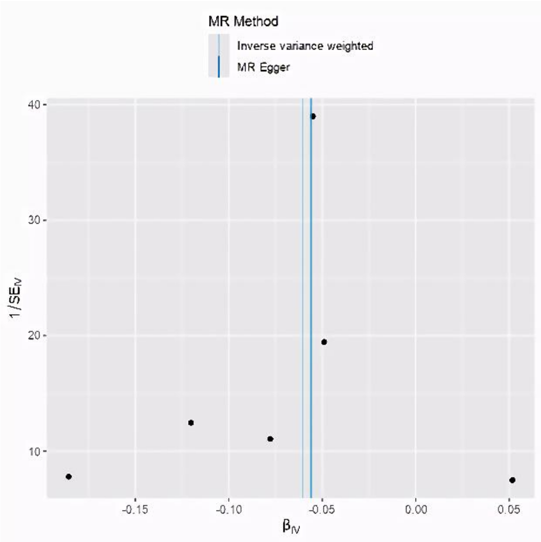
MR Method
Inverse variance weighted
MR Egger
40
30
20
10
1/SE$_{IV}$
-0.15
-0.10
-0.05
0.00
0.05
β$_{IV}$

IL10

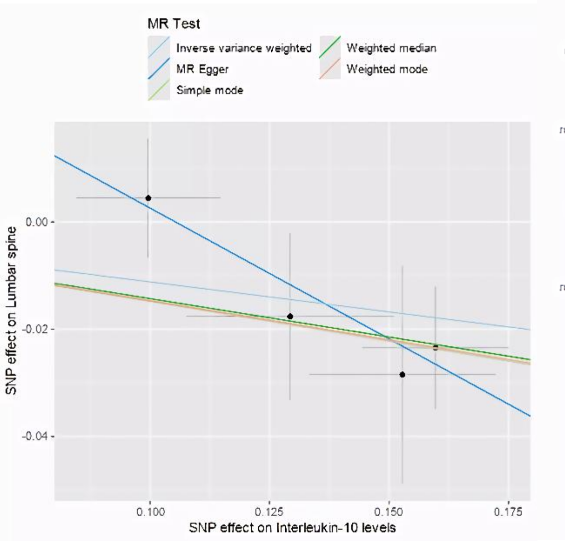
MR Test
Inverse variance weighted
MR Egger
Simple mode
Weighted median
Weighted mode
SNP effect on Lumbar spine
SNP effect on Interleukin-10 levels

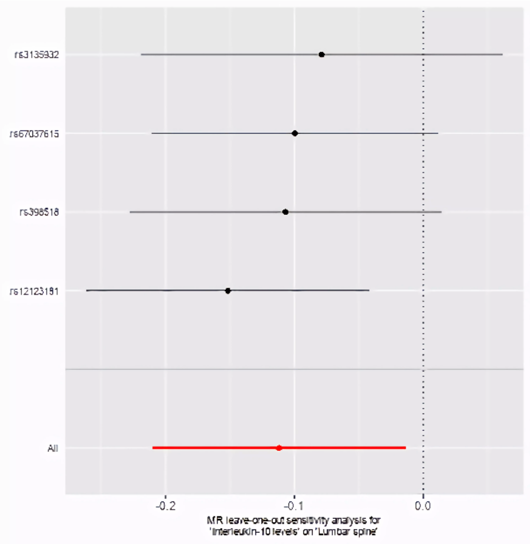
rs3135932
rs67037615
rs398518
rs12123191
All
MR leave-one-out sensitivity analysis for
'Interleukin-10 levels' on 'Lumbar spine'

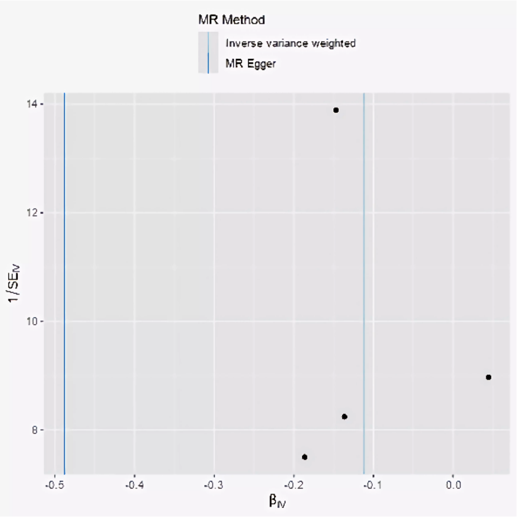
MR Method
Inverse variance weighted
MR Egger
$1/SE_{IV}$
β_{IV}

FGF19

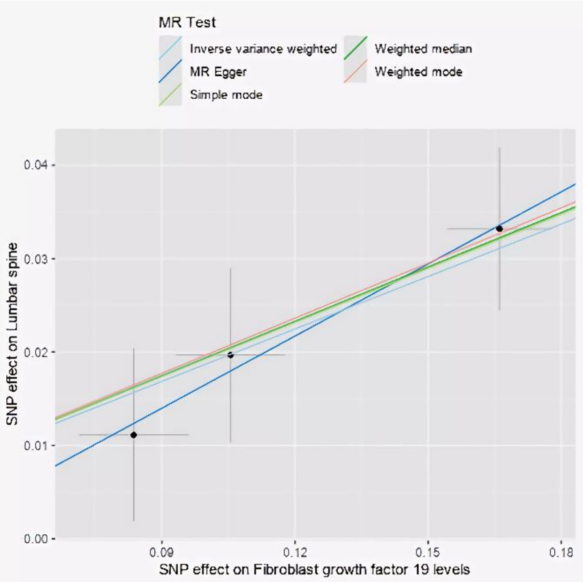
MR Test
Inverse variance weighted
MR Egger
Simple mode
Weighted median
Weighted mode
SNP effect on Lumbar spine
SNP effect on Fibroblast growth factor 19 levels

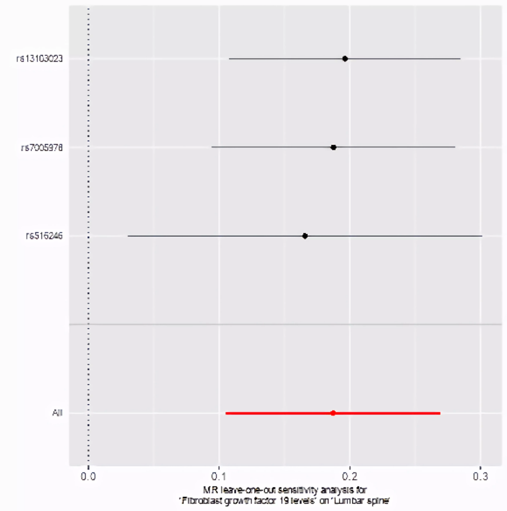
rs13103023
rs7005978
rs516246
All
MR leave-one-out sensitivity analysis for
'Fibroblast growth factor 19 levels' on 'Lumbar spine'

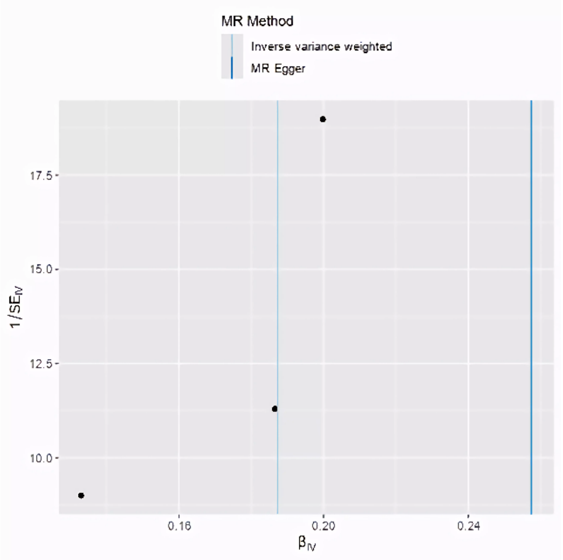

Oncostatin-M

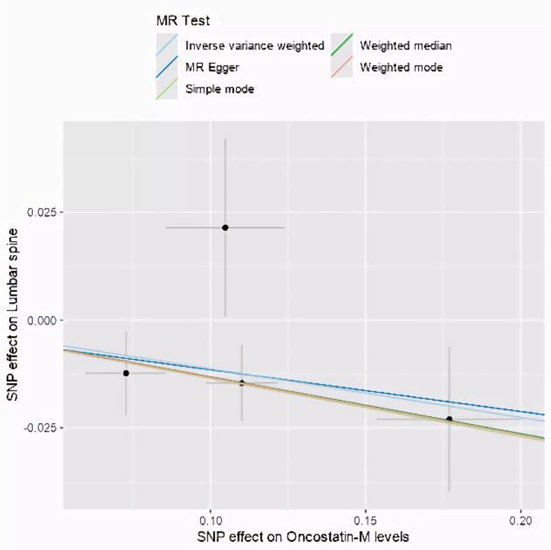

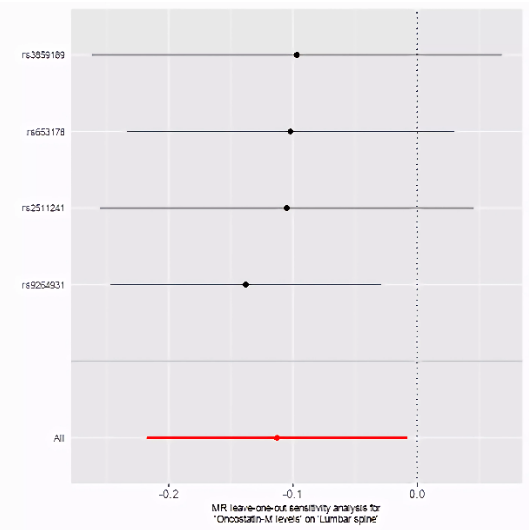

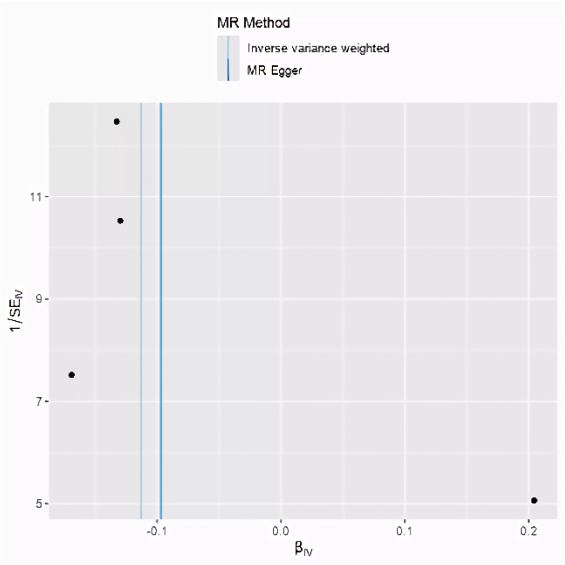

Results for HB

CCL4

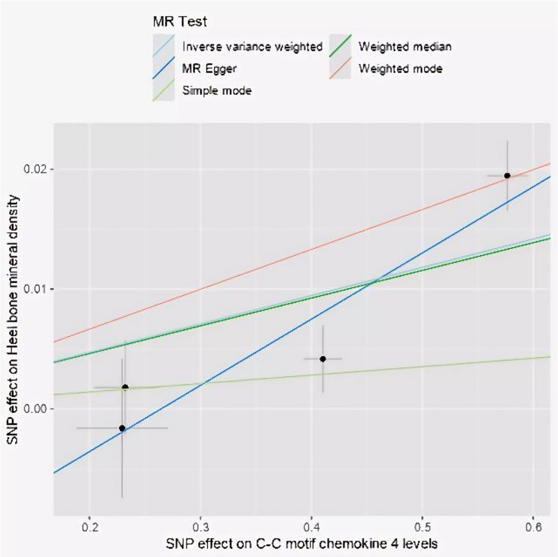

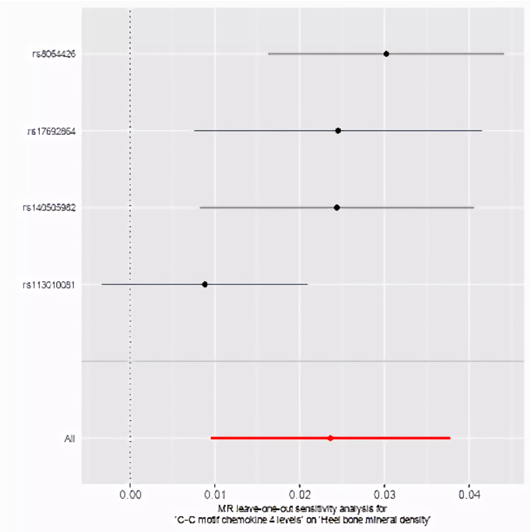

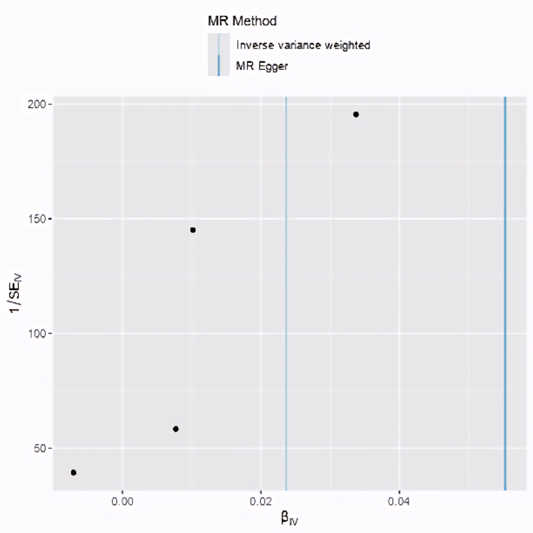

CXCL10

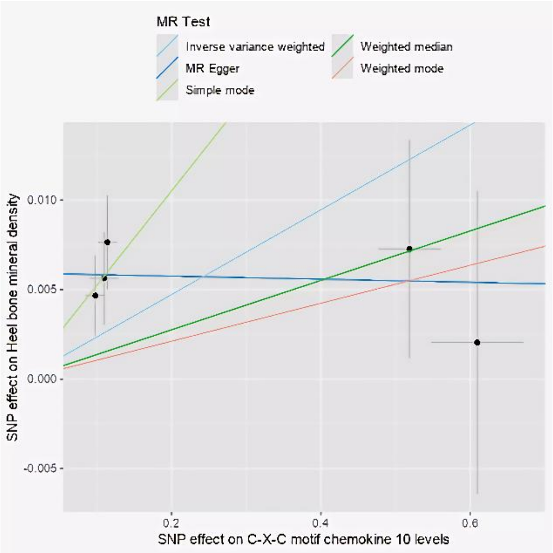

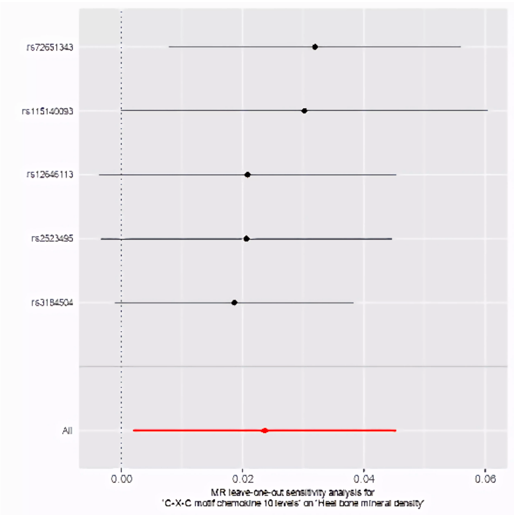

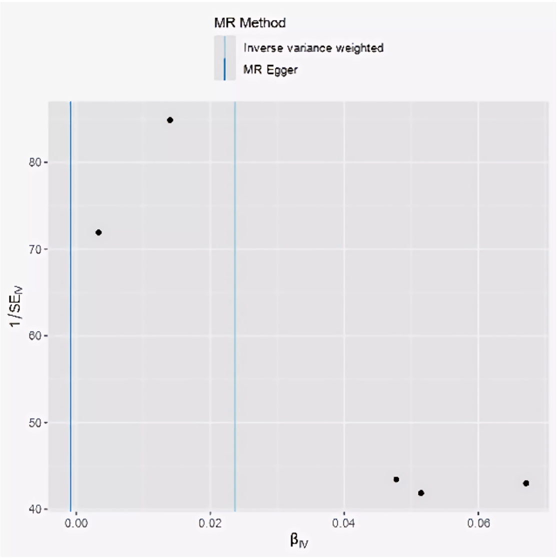
MR Method
Inverse variance weighted
MR Egger
80
70
60
50
40
1/SE$_{IV}$
0.00
0.02
0.04
0.06
β$_{IV}$

DNER

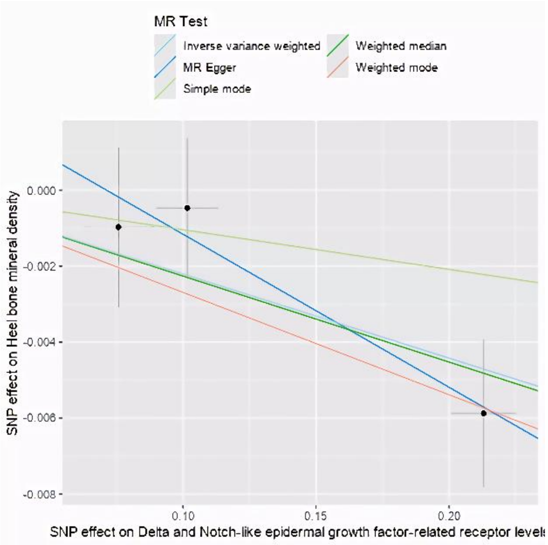
MR Test
Inverse variance weighted
MR Egger
Simple mode
Weighted median
Weighted mode
SNP effect on Heel bone mineral density
0.000
-0.002
-0.004
-0.006
-0.008
0.10
0.15
0.20
SNP effect on Delta and Notch-like epidermal growth factor-related receptor level

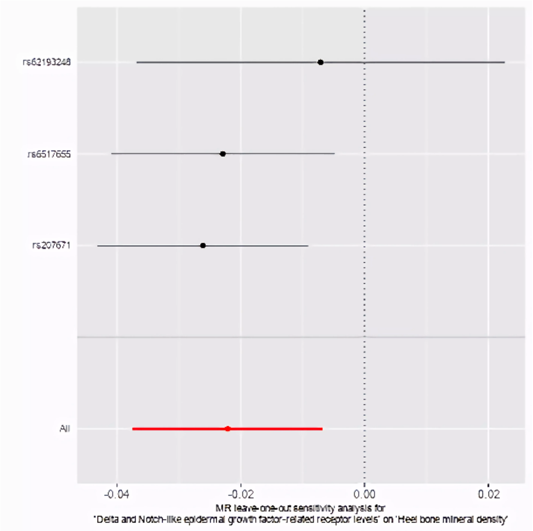
rs62193248
rs6517655
rs207671
All
-0.04
-0.02
0.00
0.02
MR leave-one-out sensitivity analysis for
'Delta and Notch-like epidermal growth factor-related receptor levels' on 'Heel bone mineral density'

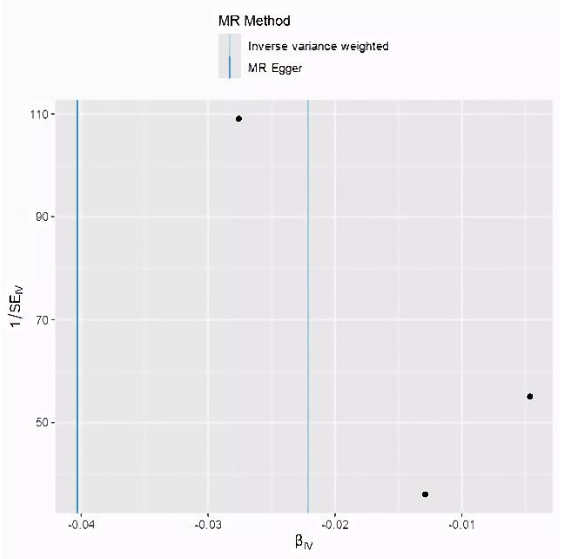
MR Method
Inverse variance weighted
MR Egger
110
90
70
50
1/SE$_{IV}$
-0.04
-0.03
-0.02
-0.01
β$_{IV}$

LIFR

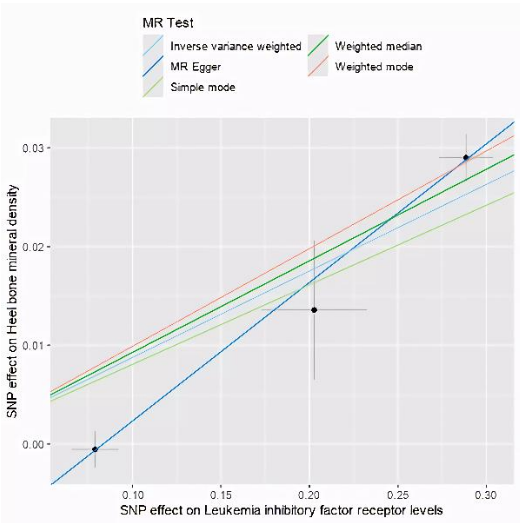
MR Test
Inverse variance weighted
MR Egger
Simple mode
Weighted median
Weighted mode
SNP effect on Heel bone mineral density
SNP effect on Leukemia inhibitory factor receptor levels

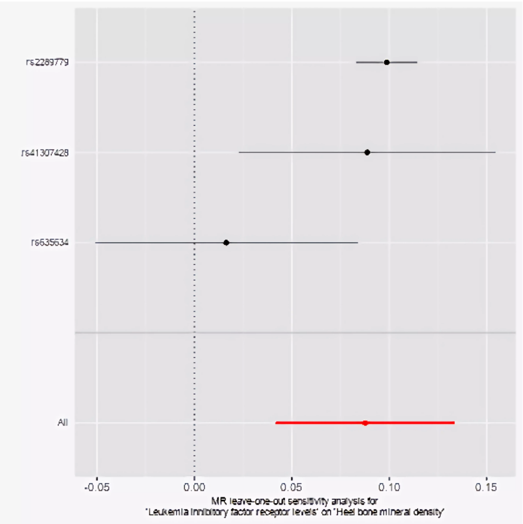
rs2289779
rs41307428
rs635634
All
MR leave-one-out sensitivity analysis for
'Leukemia inhibitory factor receptor levels' on 'Heel bone mineral density'

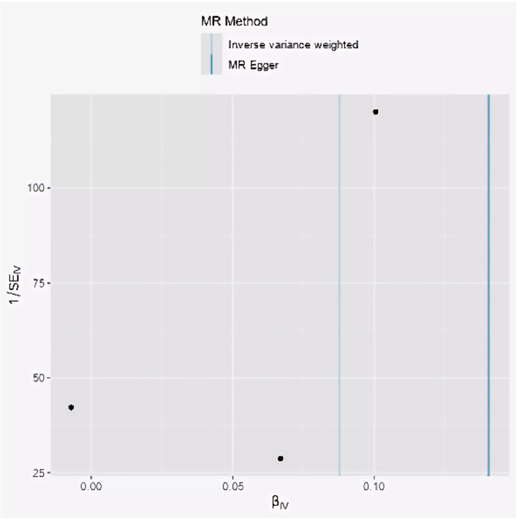
MR Method
Inverse variance weighted
MR Egger
1/SE$_{IV}$
β$_{IV}$

T cell CD5

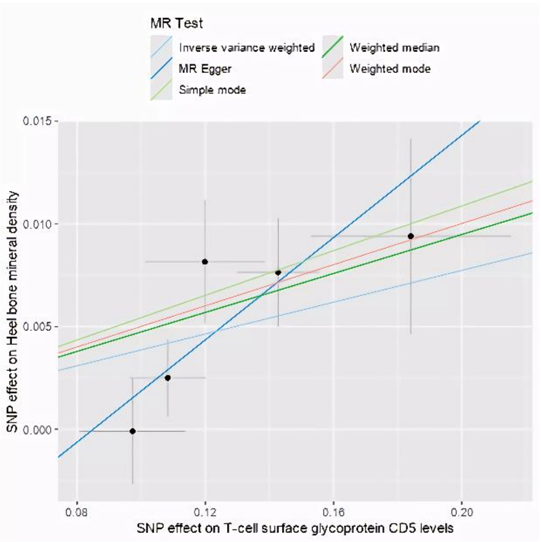
MR Test
Inverse variance weighted
MR Egger
Simple mode
Weighted median
Weighted mode
SNP effect on Heel bone mineral density
SNP effect on T-cell surface glycoprotein CD5 levels

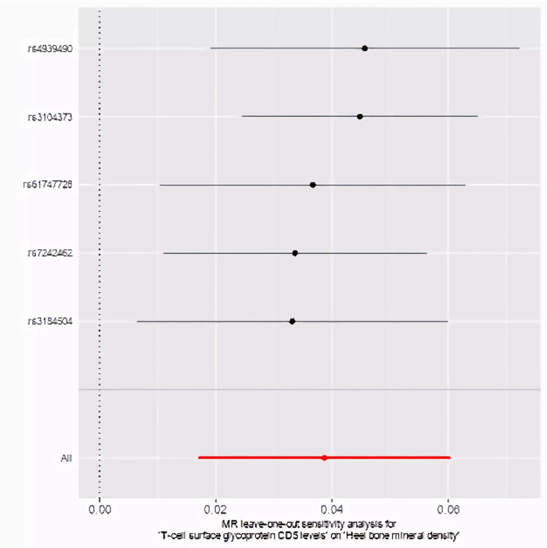
rs4939490
rs3104373
rs61747728
rs7242462
rs3184504
All
MR leave-one-out sensitivity analysis for
'T-cell surface glycoprotein CD5 levels' on 'Heel bone mineral density'

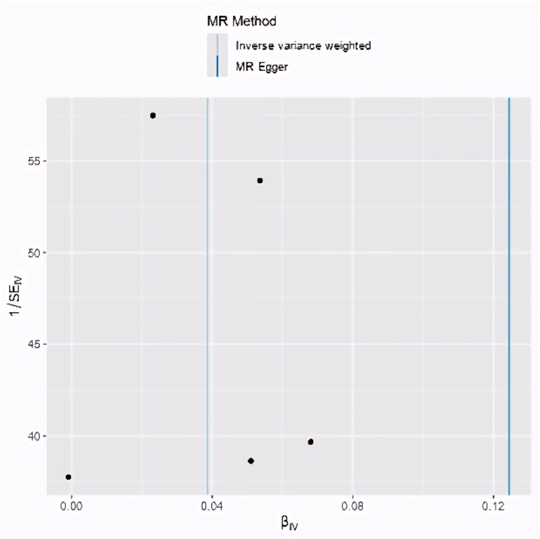

Results of Fracture

Oncostatin-M

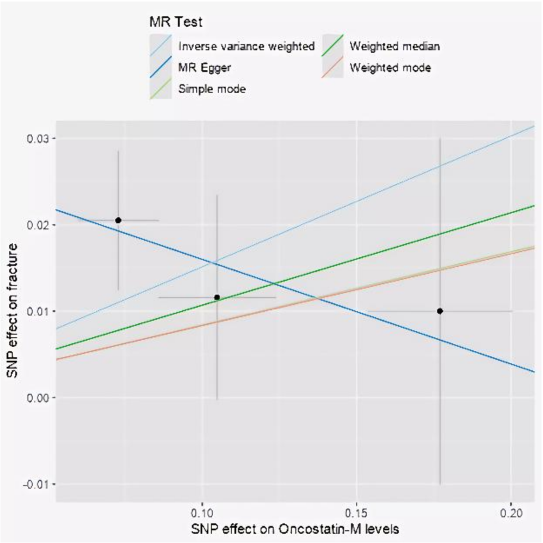

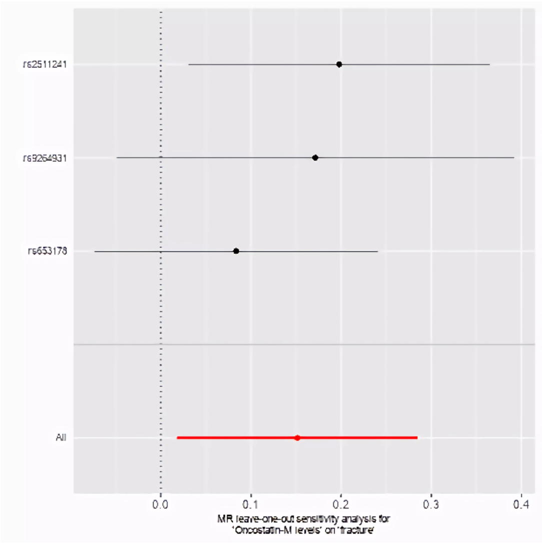

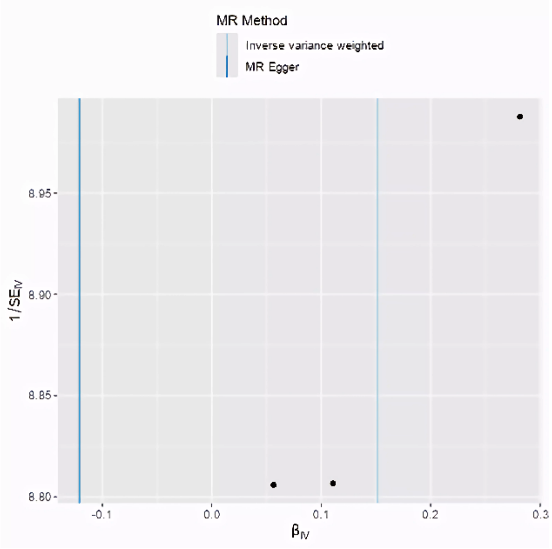

Results of ALM

CXCL6

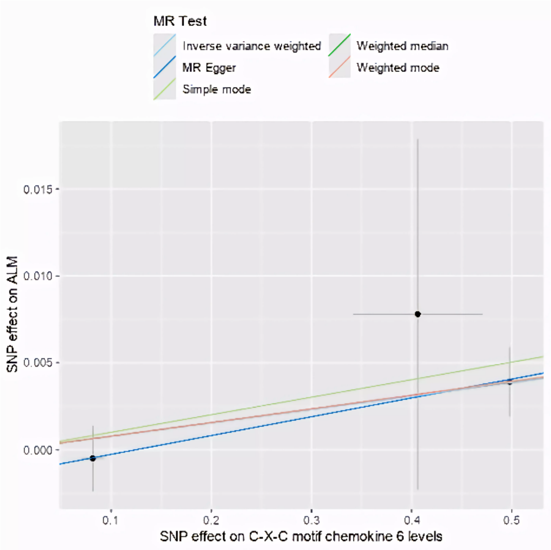
MR Test
Inverse variance weighted
MR Egger
Simple mode
Weighted median
Weighted mode
SNP effect on ALM
0.015
0.010
0.005
0.000
0.1
0.2
0.3
0.4
0.5
SNP effect on C-X-C motif chemokine 6 levels

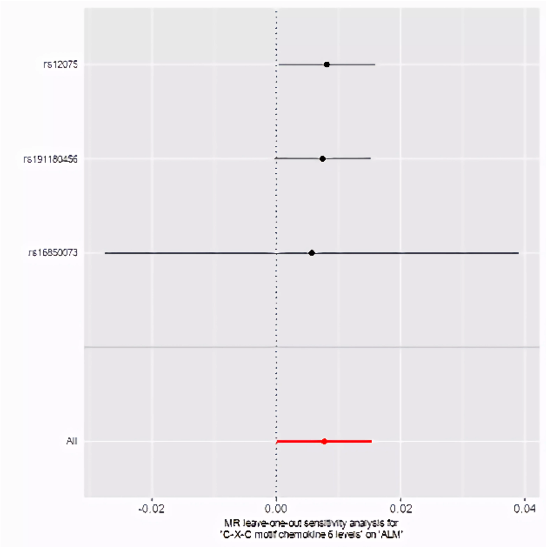
rs12075
rs191180456
rs16850073
All
-0.02
0.00
0.02
0.04
MR leave-one-out sensitivity analysis for
'C-X-C motif chemokine 6 levels' on 'ALM'

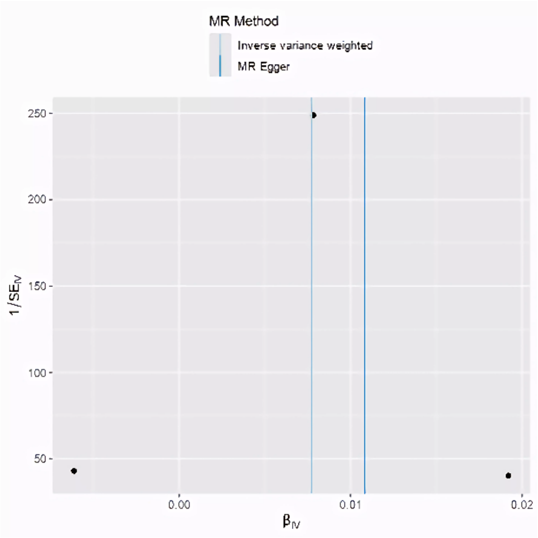
MR Method
Inverse variance weighted
MR Egger
250
200
150
100
50
0.00
0.01
0.02

DNER

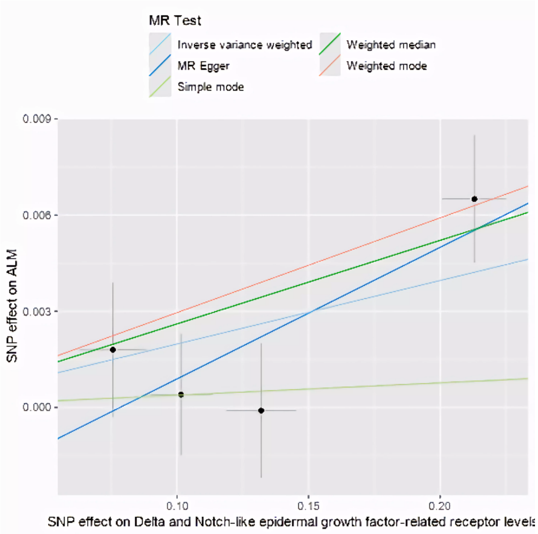
MR Test
Inverse variance weighted
MR Egger
Simple mode
Weighted median
Weighted mode
SNP effect on ALM
0.009
0.006
0.003
0.000
0.10
0.15
0.20
SNP effect on Delta and Notch-like epidermal growth factor-related receptor levels

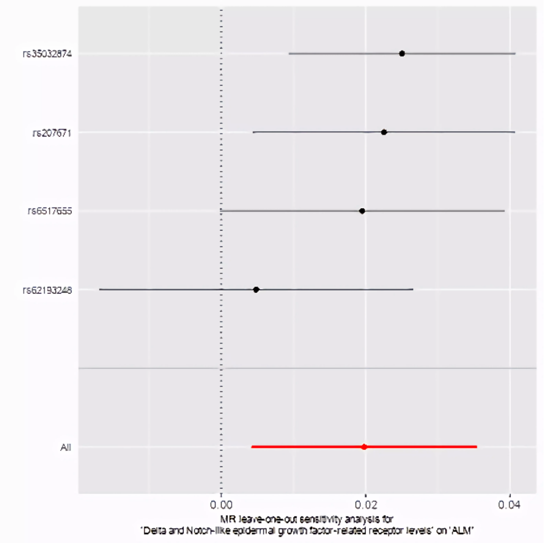
rs35032874
rs207671
rs6517655
rs62193248
All
0.00
0.02
0.04
MR leave-one-out sensitivity analysis for
'Delta and Notch-like epidermal growth factor-related receptor levels' on 'ALM'

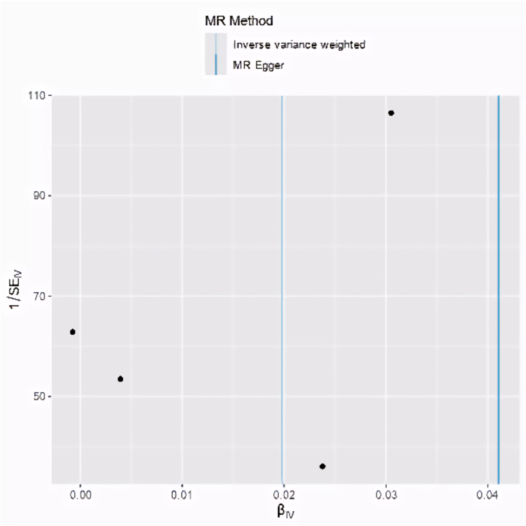
MR Method
Inverse variance weighted
MR Egger
110
90
70
50
1/SE$_{IV}$
0.00
0.01
0.02
0.03
0.04
β$_{IV}$

FGF5

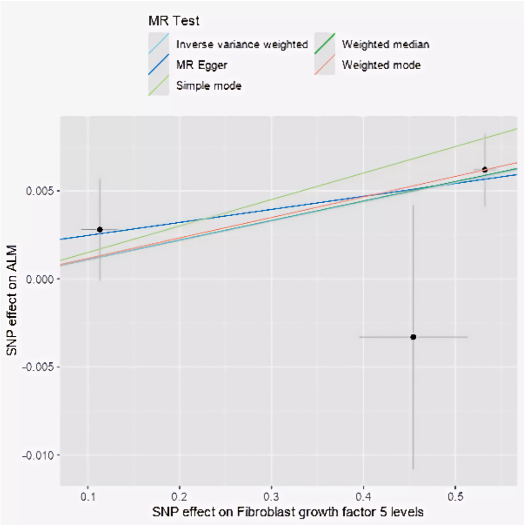
MR Test
Inverse variance weighted
MR Egger
Simple mode
Weighted median
Weighted mode
0.005
0.000
-0.005
-0.010
SNP effect on ALM
0.1
0.2
0.3
0.4
0.5
SNP effect on Fibroblast growth factor 5 levels

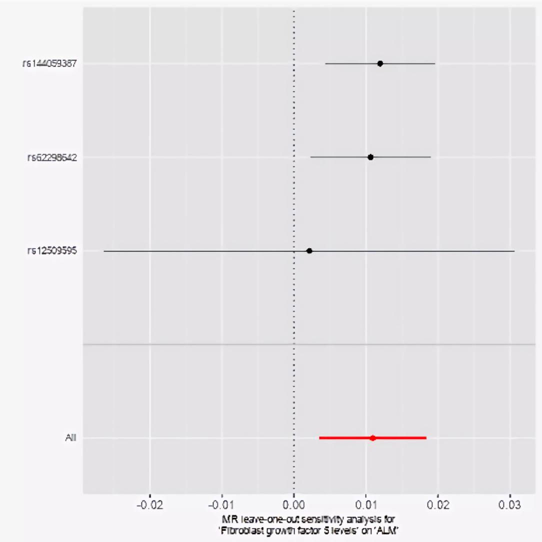
rs144059387
rs62298642
rs12509595
All
-0.02
-0.01
0.00
0.01
0.02
0.03
MR leave-one-out sensitivity analysis for
'Fibroblast growth factor 5 levels' on 'ALM'

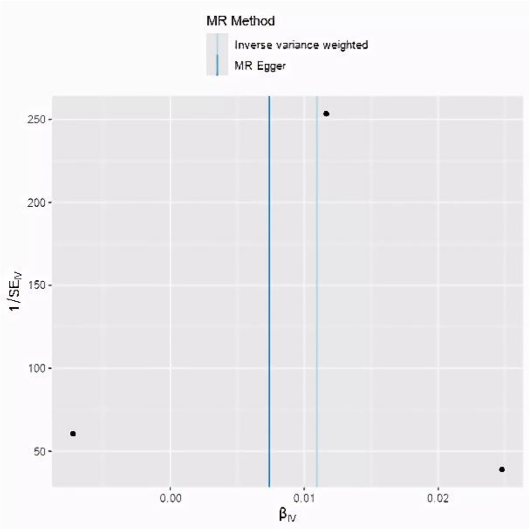
MR Method
Inverse variance weighted
MR Egger
250
200
150
100
50
1/SE$_{IV}$
0.00
0.01
0.02
β$_{IV}$

GDNF

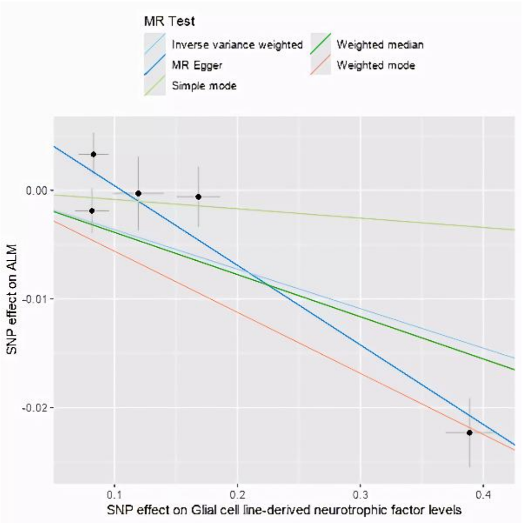
MR Test
Inverse variance weighted
MR Egger
Simple mode
Weighted median
Weighted mode
SNP effect on ALM
0.00
-0.01
-0.02
0.1
0.2
0.3
0.4
SNP effect on Glial cell line-derived neurotrophic factor levels

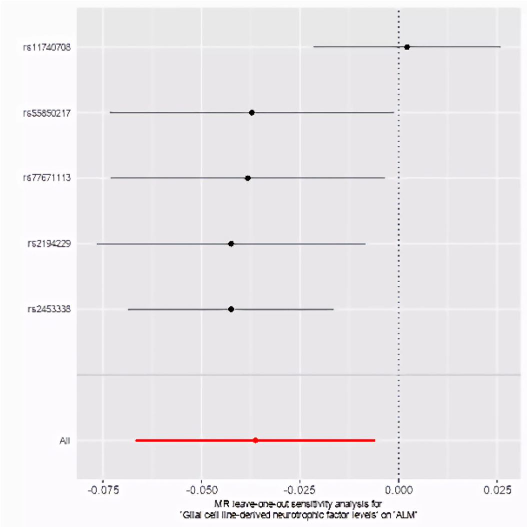
rs11740708
rs55850217
rs77671113
rs2194229
rs2453338
All
-0.075
-0.050
-0.025
0.000
0.025
MR leave-one-out sensitivity analysis for
'Glial cell line-derived neurotrophic factor levels' on 'ALM'

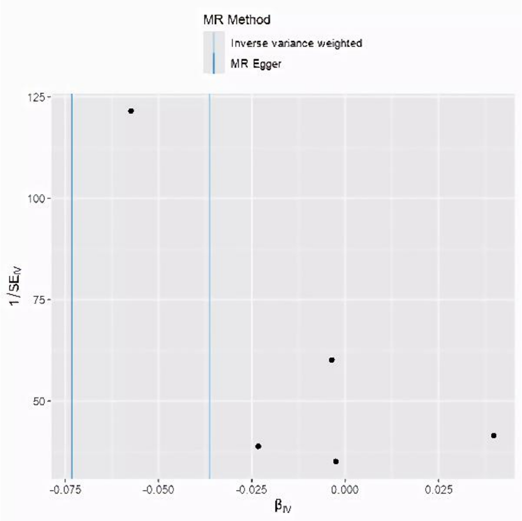
MR Method
Inverse variance weighted
MR Egger
125
100
75
50
$1/SE_{IV}$
-0.075
-0.050
-0.025
0.000
0.025
β_{IV}

IL10RB

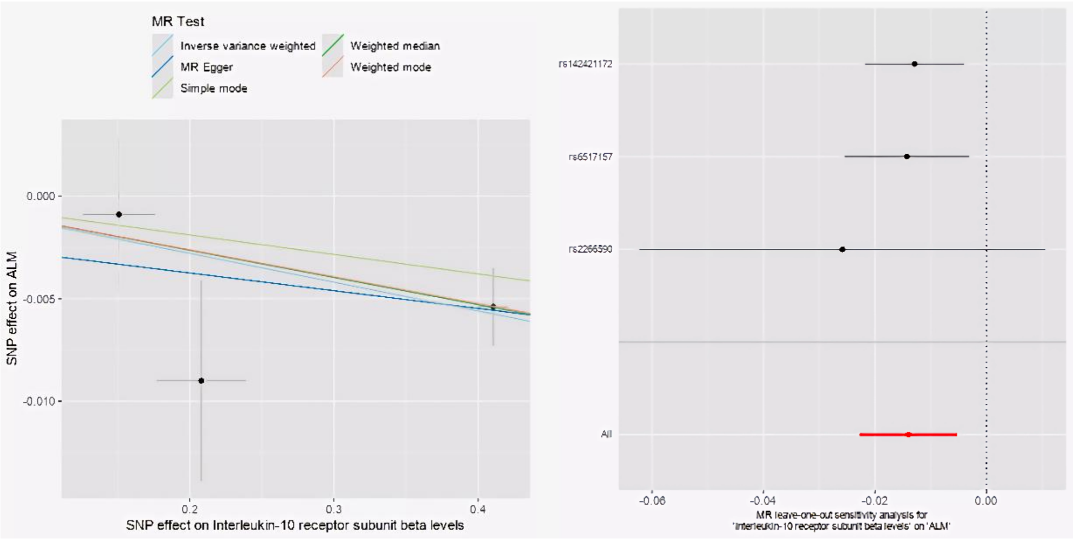
MR Test
Inverse variance weighted
MR Egger
Simple mode
Weighted median
Weighted mode
SNP effect on ALM
0.000
-0.005
-0.010
0.2
0.3
0.4
SNP effect on Interleukin-10 receptor subunit beta levels
rs142421172
rs6517157
rs2266590
All
-0.06
-0.04
-0.02
0.00
MR leave-one-out sensitivity analysis for
'Interleukin-10 receptor subunit beta levels' on 'ALM'

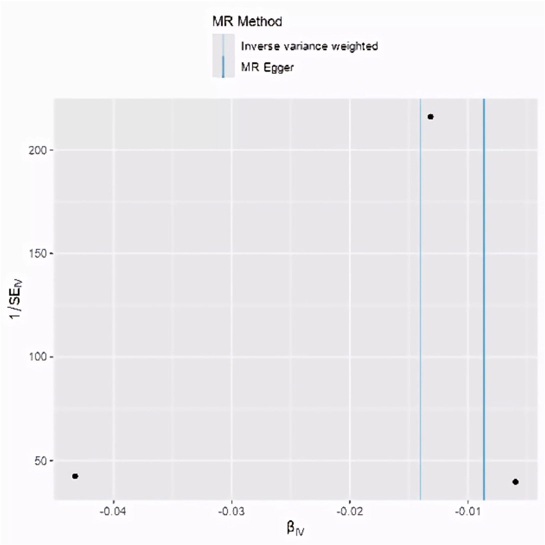
MR Method
Inverse variance weighted
MR Egger
200
150
100
50
1/SE$_{IV}$
-0.04
-0.03
-0.02
-0.01
β$_{IV}$

LTA

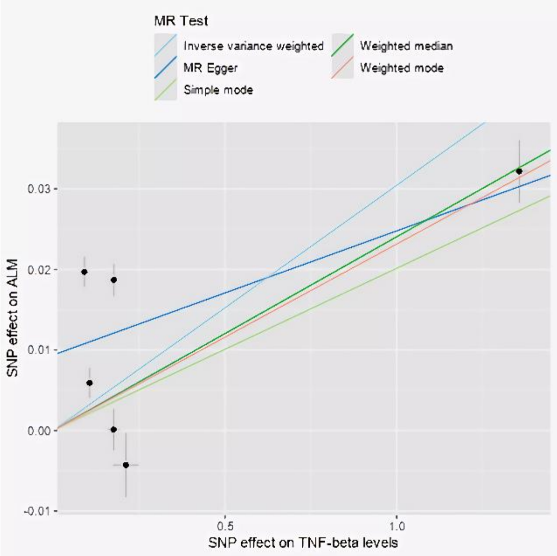
MR Test
Inverse variance weighted
MR Egger
Simple mode
Weighted median
Weighted mode
0.03
0.02
0.01
0.00
-0.01
SNP effect on ALM
0.5
1.0
SNP effect on TNF-beta levels

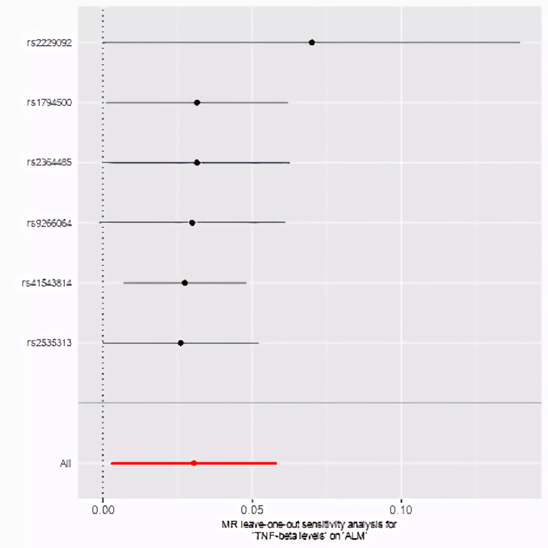
rs2229092
rs1794500
rs2364485
rs9266064
rs41543814
rs2535313
All
0.00
0.05
0.10
MR leave-one-out sensitivity analysis for
'TNF-beta levels' on 'ALM'

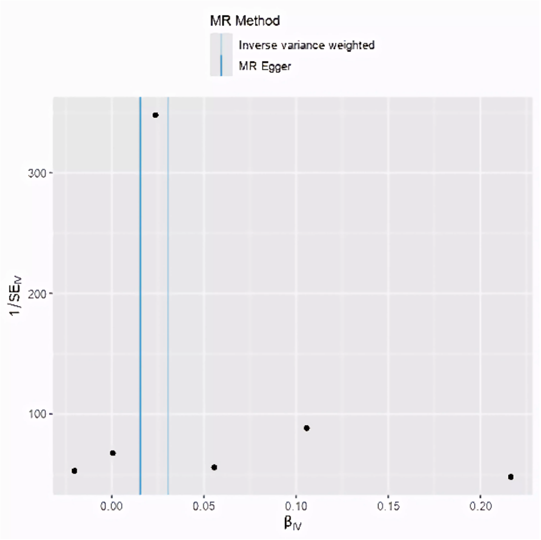
MR Method
Inverse variance weighted
MR Egger
300
200
100
1/SE$_{IV}$
0.00
0.05
0.10
0.15
0.20
β$_{IV}$

TNFSF12

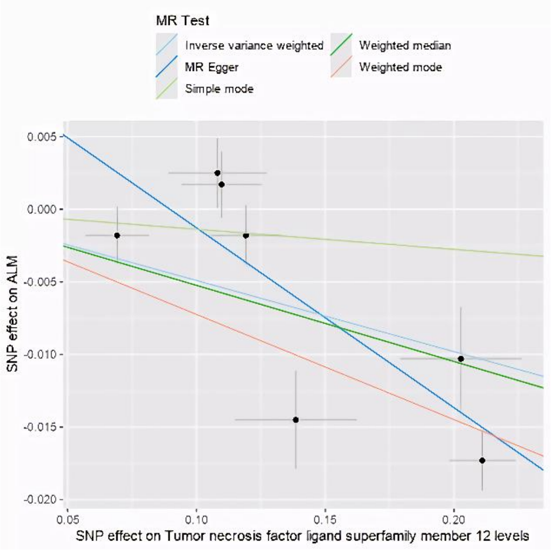

MR Test
Inverse variance weighted
MR Egger
Simple mode
Weighted median
Weighted mode
0.005
0.000
-0.005
-0.010
-0.015
-0.020
SNP effect on ALM
0.05
0.10
0.15
0.20
SNP effect on Tumor necrosis factor ligand superfamily member 12 levels

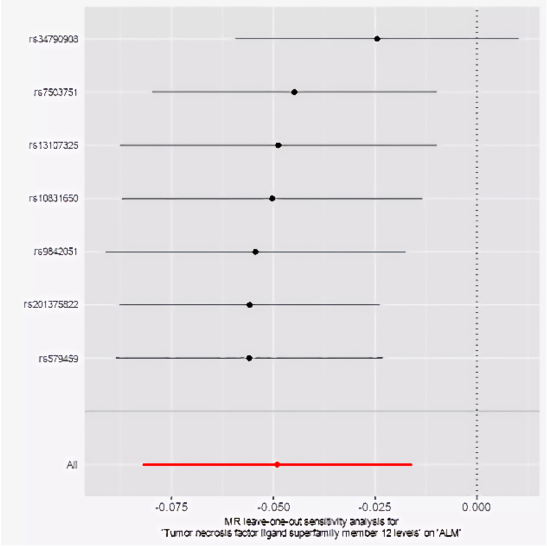

rs34790908
rs7503751
rs13107325
rs10831650
rs9842051
rs201375822
rs579459
All
-0.075
-0.050
-0.025
0.000
MR leave-one-out sensitivity analysis for
'Tumor necrosis factor ligand superfamily member 12 levels' on 'ALM'

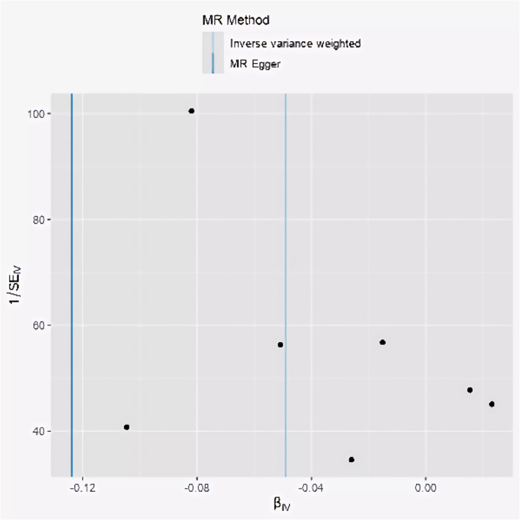

MR Method
Inverse variance weighted
MR Egger
100
80
60
40
1/SE$_{IV}$
-0.12
-0.08
-0.04
0.00
β_{IV}

Results of LGS

T-cell CD6

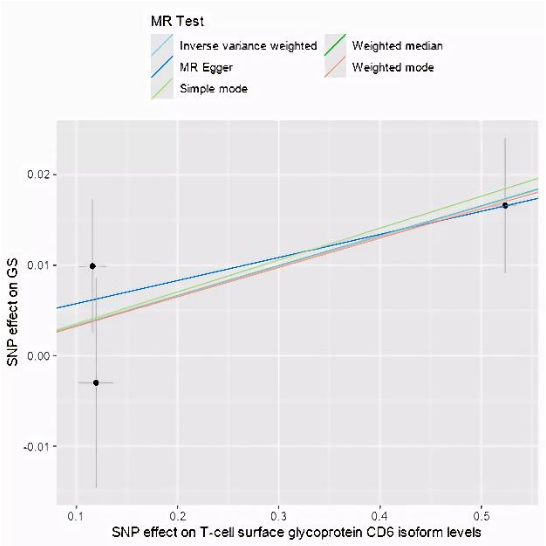

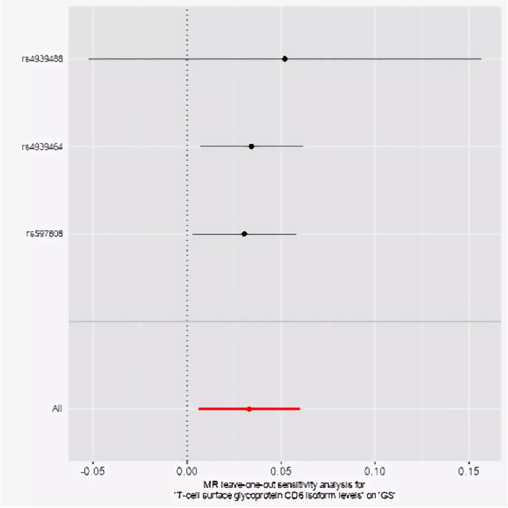

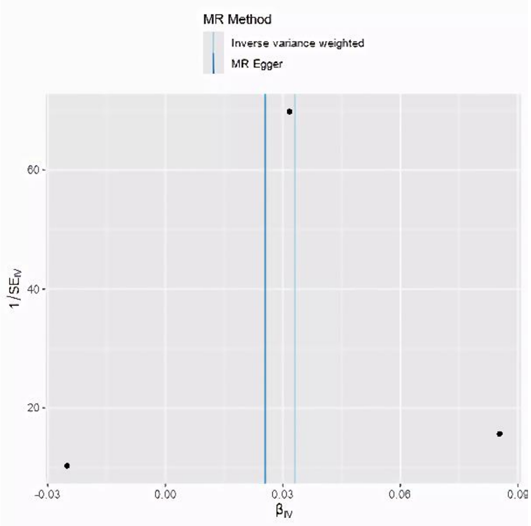

MCP2

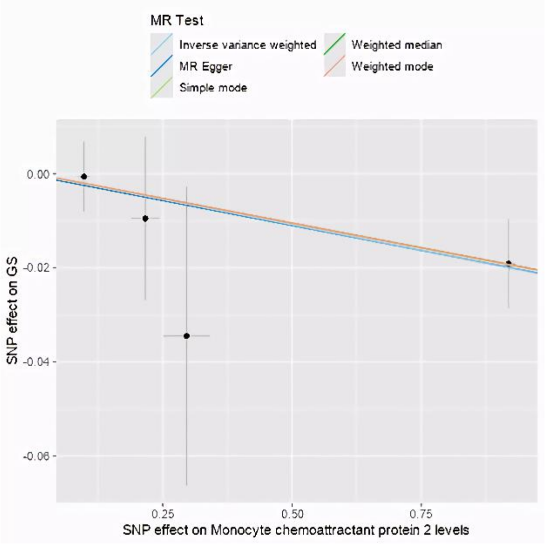

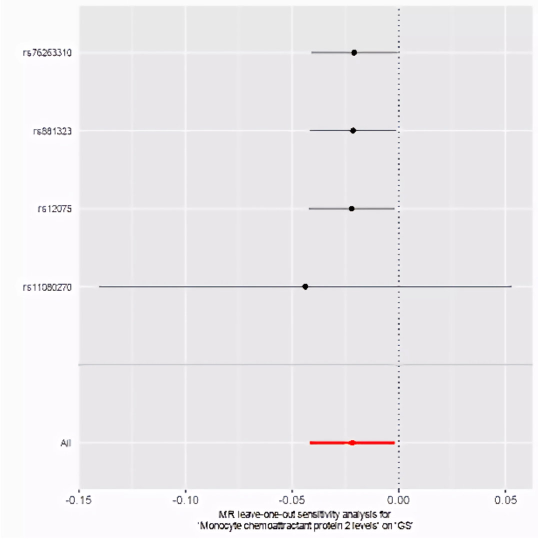

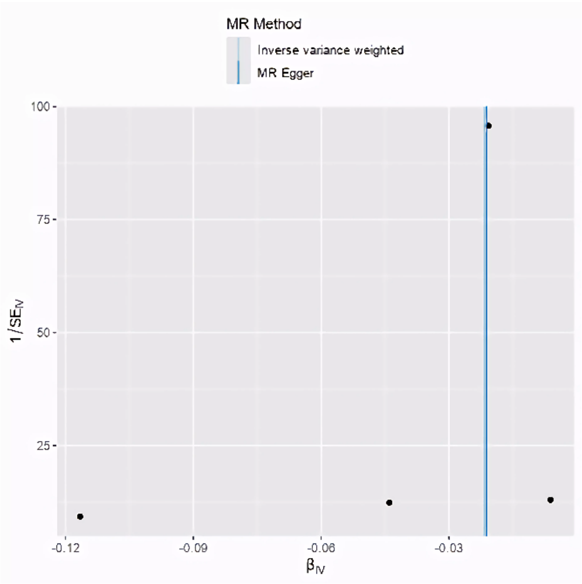
MR Method
Inverse variance weighted
MR Egger
1/SE$_{IV}$
β_{IV}

LTA

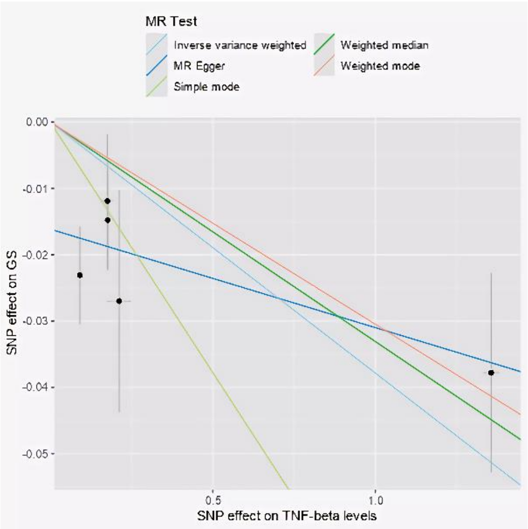
MR Test
Inverse variance weighted
MR Egger
Simple mode
Weighted median
Weighted mode
SNP effect on GS
SNP effect on TNF-beta levels

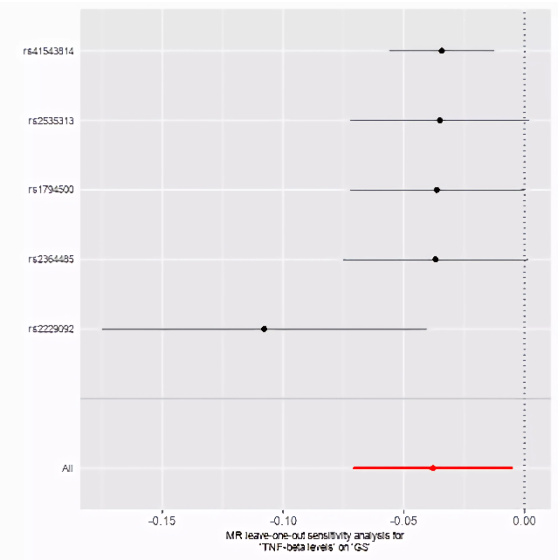
rs41543814
rs2535313
rs1794500
rs2364485
rs2229092
All
MR leave-one-out sensitivity analysis for
'TNF-beta levels' on 'GS'

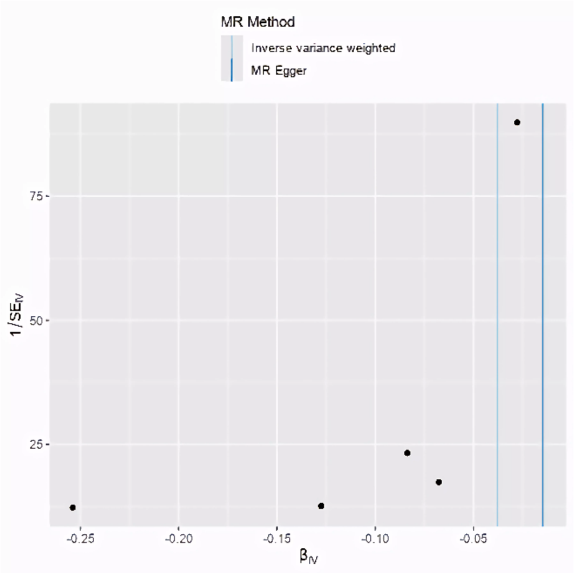
MR Method
Inverse variance weighted
MR Egger
1/SE$_{IV}$
β_{IV}

Results of WP

CD40

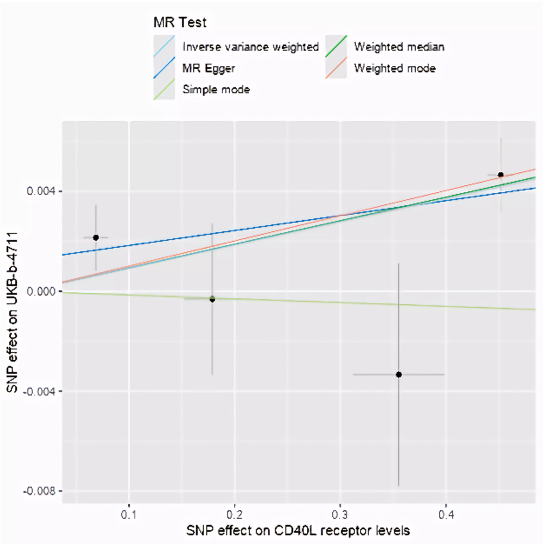

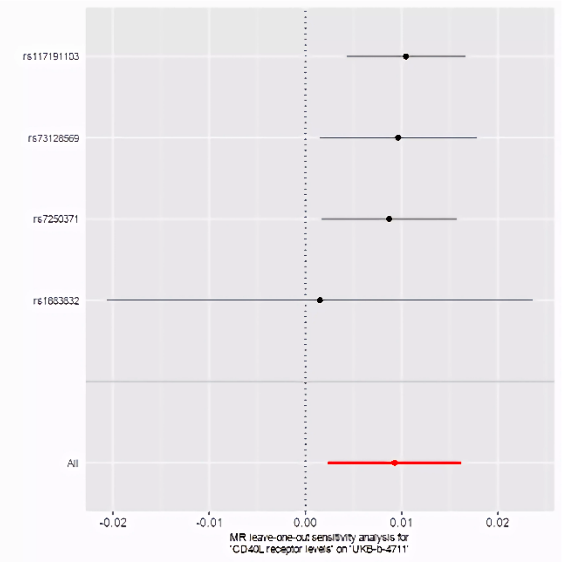

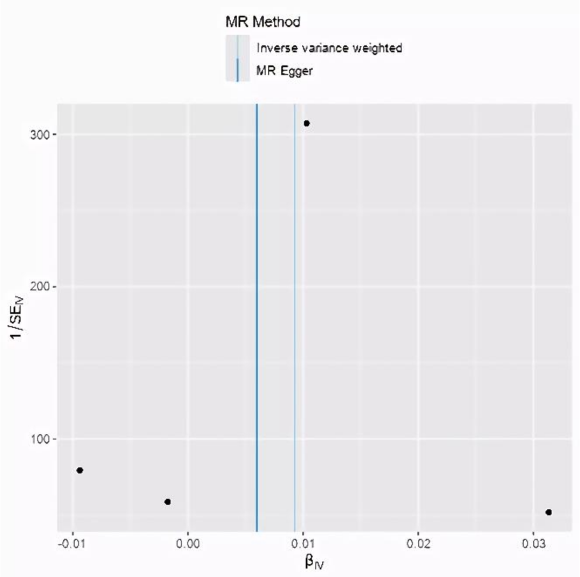

CX3CL1

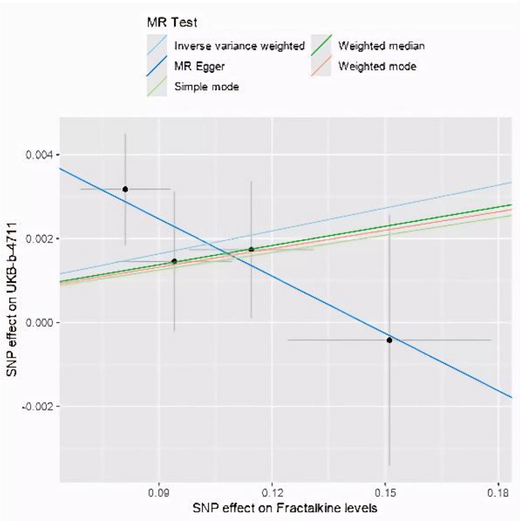

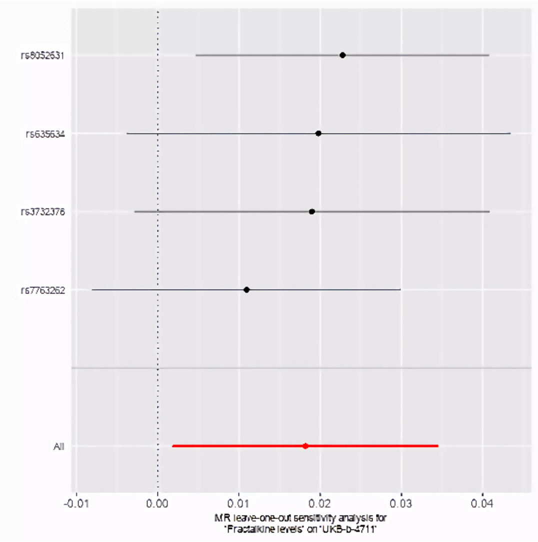

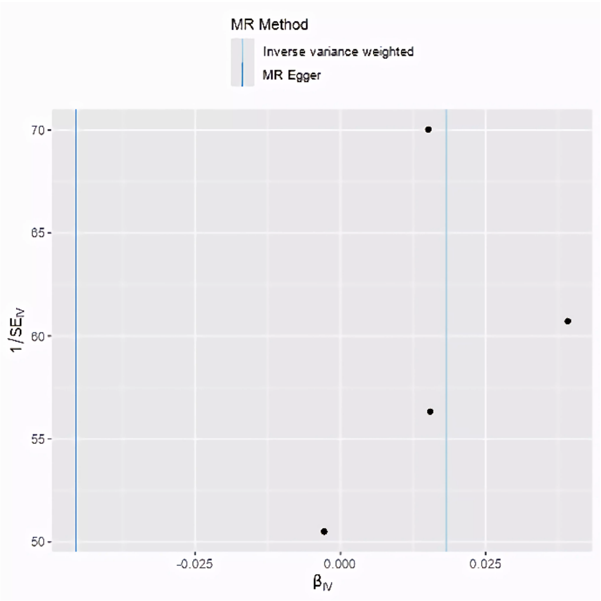
MR Method
Inverse variance weighted
MR Egger
1/SE$_{IV}$
β_{IV}

CXCL10

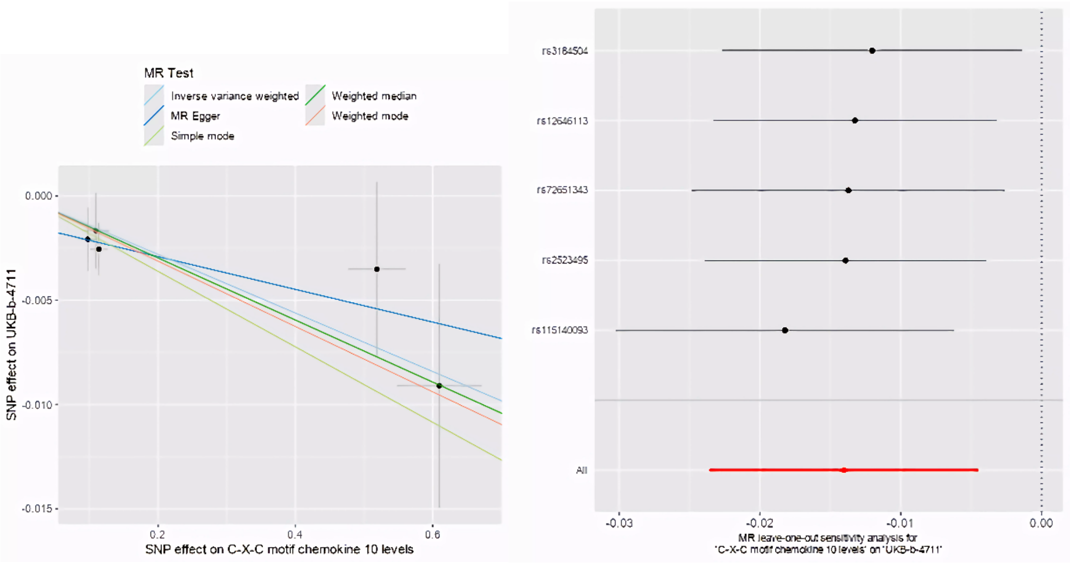
MR Test
Inverse variance weighted
MR Egger
Simple mode
Weighted median
Weighted mode
SNP effect on UKB-b-4711
SNP effect on C-X-C motif chemokine 10 levels
rs3184504
rs12646113
rs72651343
rs2523495
rs115140093
All
MR leave-one-out sensitivity analysis for
'C-X-C motif chemokine 10 levels' on 'UKB-b-4711'

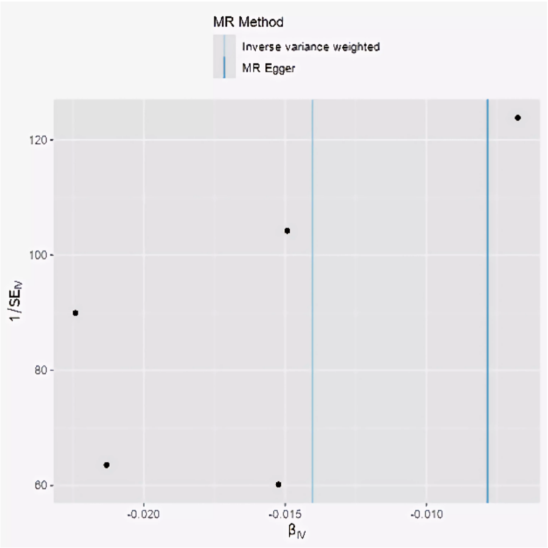
MR Method
Inverse variance weighted
MR Egger
1/SE$_{IV}$
β_{IV}

FLT3L

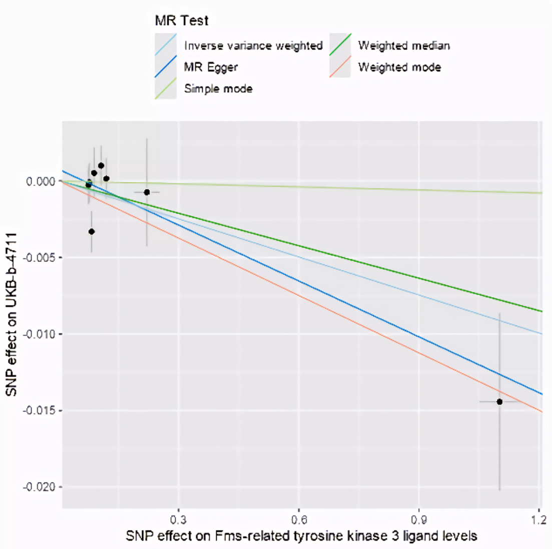
MR Test
Inverse variance weighted
MR Egger
Simple mode
Weighted median
Weighted mode
SNP effect on UKB-b-4711
0.000
-0.005
-0.010
-0.015
-0.020
0.3
0.6
0.9
1.2
SNP effect on Fms-related tyrosine kinase 3 ligand levels

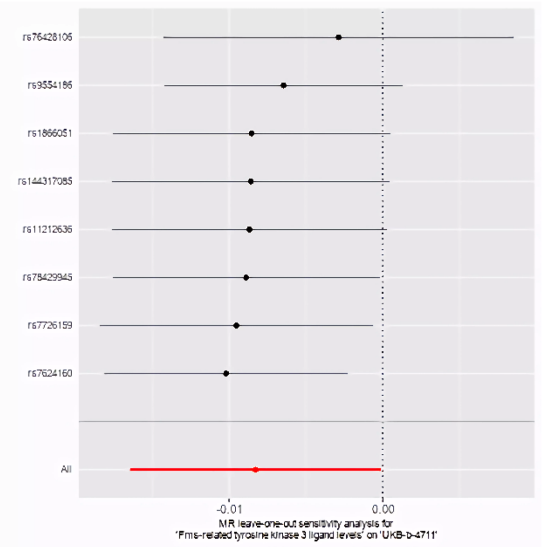
rs76428106
rs9554186
rs1866051
rs144317085
rs11212636
rs78429945
rs7726159
rs7624160
All
-0.01
0.00
MR leave-one-out sensitivity analysis for
'Fms-related tyrosine kinase 3 ligand levels' on 'UKB-b-4711'

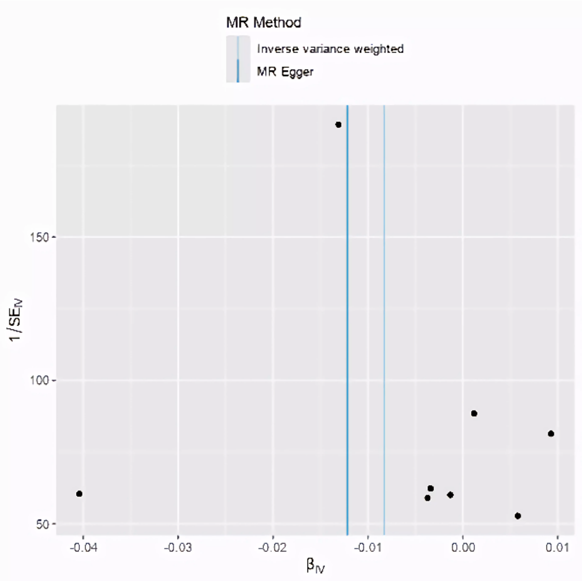
MR Method
Inverse variance weighted
MR Egger
$1/SE_{IV}$
150
100
50
-0.04
-0.03
-0.02
-0.01
0.00
0.01
β_{IV}

LTA

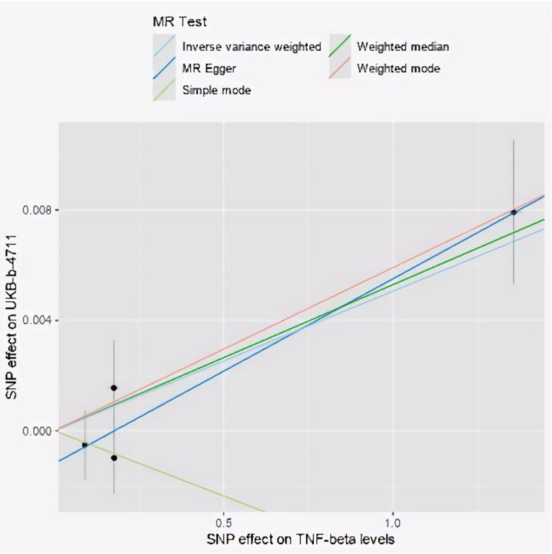
MR Test
Inverse variance weighted
MR Egger
Simple mode
Weighted median
Weighted mode
SNP effect on UKB-b-4711
0.008
0.004
0.000
0.5
1.0
SNP effect on TNF-beta levels

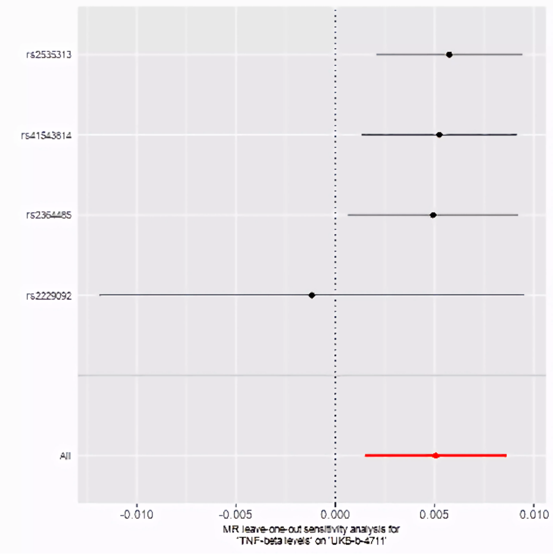
rs2535313
rs41543814
rs2364485
rs2229092
All
-0.010
-0.005
0.000
0.005
0.010
MR leave-one-out sensitivity analysis for
'TNF-beta levels' on 'UKB-b-4711'

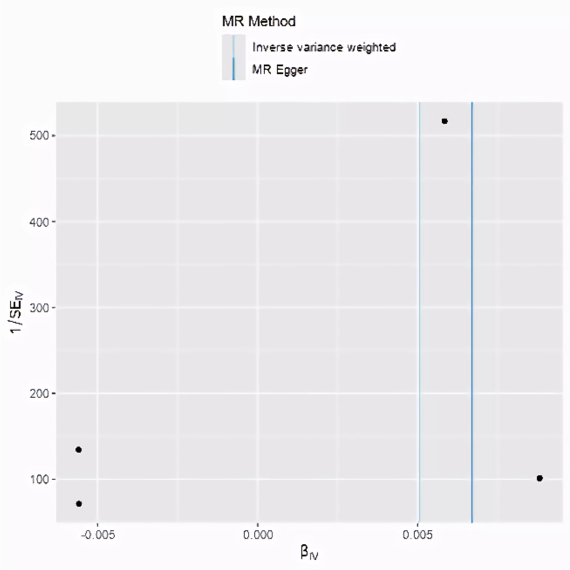
MR Method
Inverse variance weighted
MR Egger
500
400
300
200
100
1/SE$_{IV}$
-0.005
0.000
0.005
β$_{IV}$

SLAM

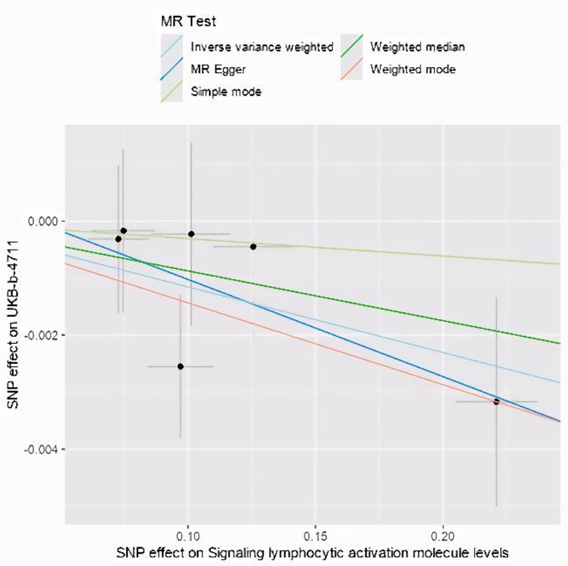
MR Test
Inverse variance weighted
MR Egger
Simple mode
Weighted median
Weighted mode
0.000
-0.002
-0.004
SNP effect on UKB-b-4711
0.10
0.15
0.20
SNP effect on Signaling lymphocytic activation molecule levels

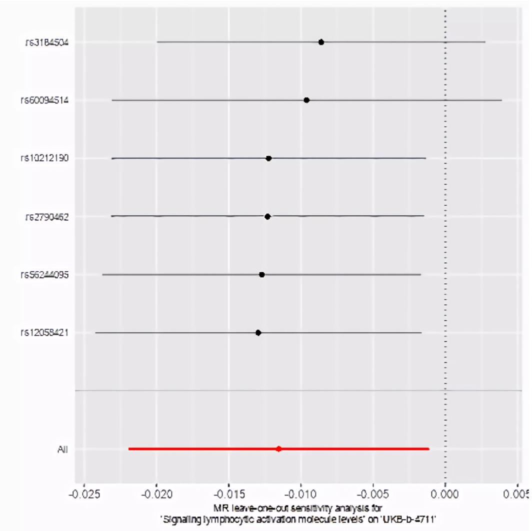
rs3184504
rs60094514
rs10212190
rs2790462
rs56244095
rs12058421
All
-0.025
-0.020
-0.015
-0.010
-0.005
0.000
MR leave-one-out sensitivity analysis for
'Signaling lymphocytic activation molecule levels' on 'UKB-b-4711'

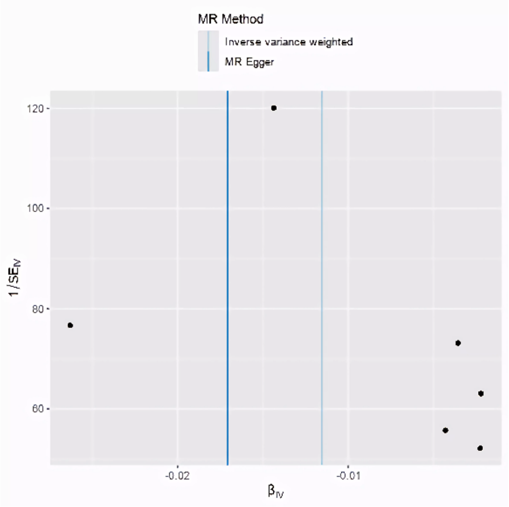
MR Method
Inverse variance weighted
MR Egger
120
100
80
60
1/SE$_{IV}$
-0.02
-0.01
β$_{IV}$
