# Supplementary material for: Causal Associations of Inflammatory Cytokines With Osteosarcopenia: Insights From Mendelian Randomization and Single Cell Analysis
Source: Mediators Inflamm. 2025 Apr 3;2025:6005225. doi: 10.1155/mi/6005225 (PMC11986192; doi:10.1155/mi/6005225)
Supplement: Supporting Information 3 — Figure S2: MR results of main analysis B. The figure for each analysis was arranged in the following order: (1) comparison of results using different MR methods; (2) leave-one-out sensitivity analysis; and (3) funnel plot of MR analysis. [file 6005225.f3.pdf]

Figure S2: MR results of Main analysis B. The figure for each analysis were arranged in the following order: 1. Comparison of results using different MR methods; 2. Leave-one-out sensitivity analysis; 3. Funnel plot of MR analysis.

Results for FA

ARTN

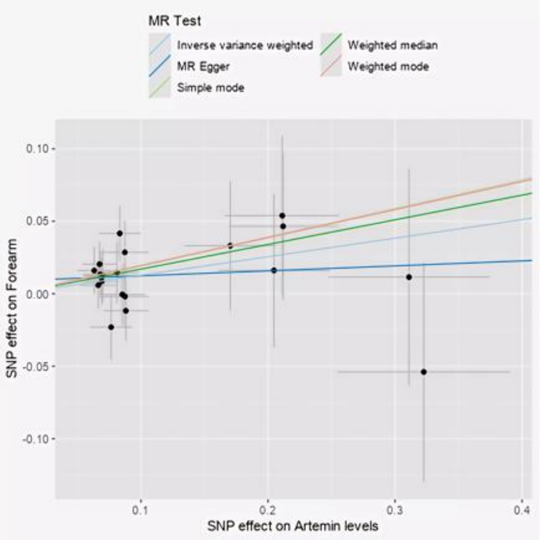

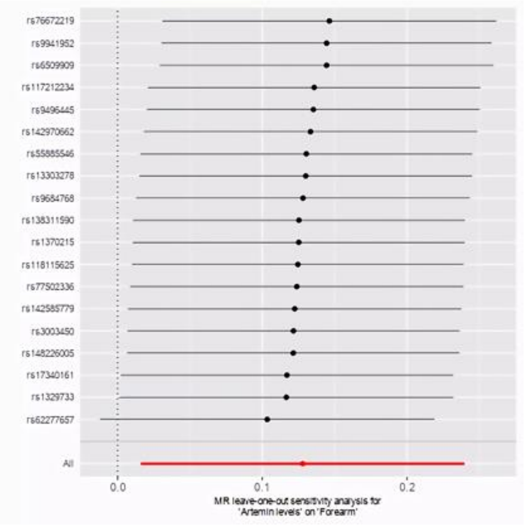

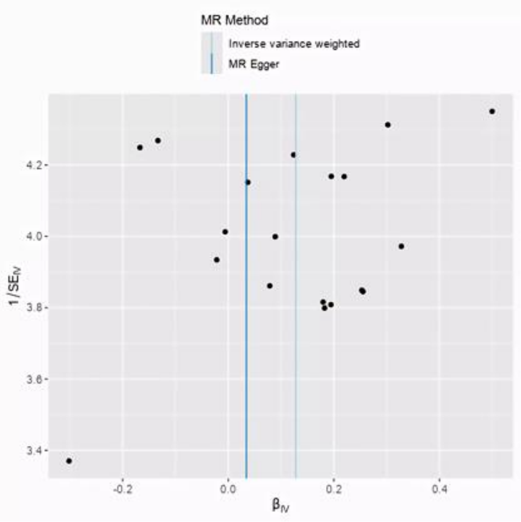

CD40

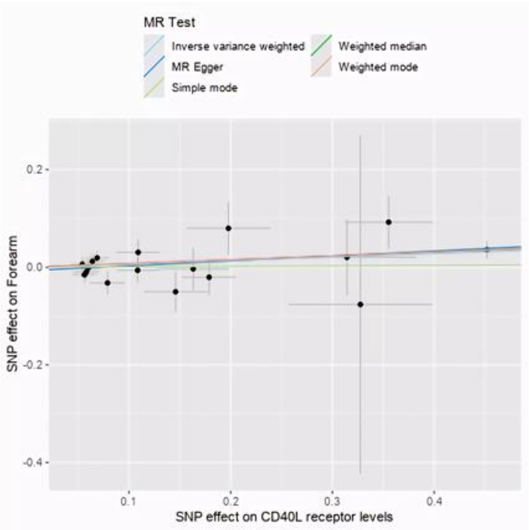
MR Test
Inverse variance weighted
MR Egger
Simple mode
Weighted median
Weighted mode
SNP effect on Forearm
SNP effect on CD40L receptor levels

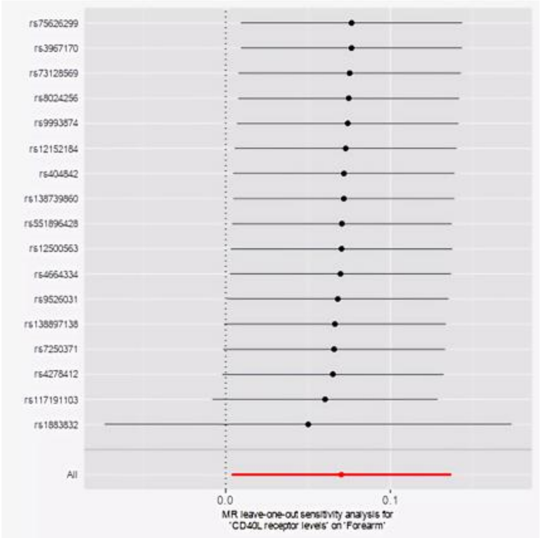
All
MR leave-one-out sensitivity analysis for
'CD40L receptor levels' on 'Forearm'

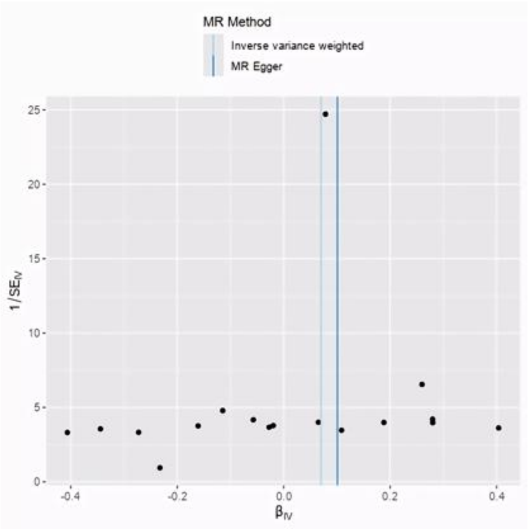
MR Method
Inverse variance weighted
MR Egger

CXCL10

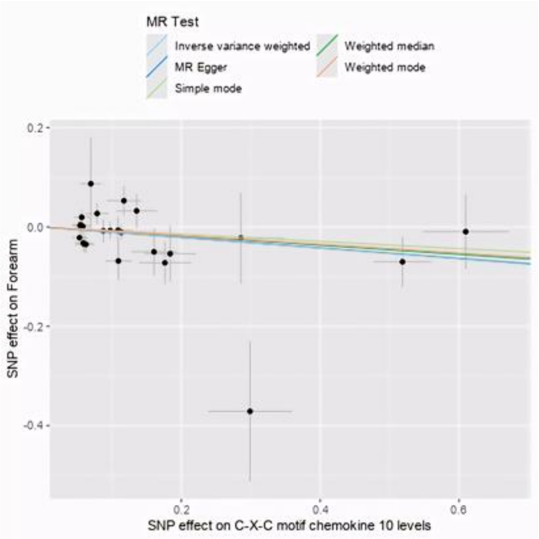
MR Test
Inverse variance weighted
MR Egger
Simple mode
Weighted median
Weighted mode
SNP effect on Forearm
SNP effect on C-X-C motif chemokine 10 levels

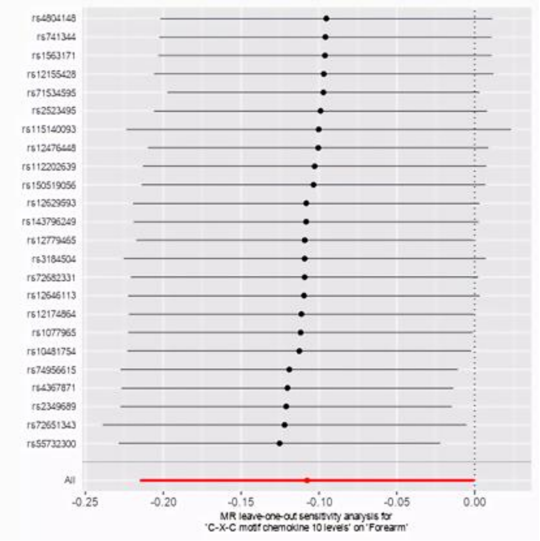
All
MR leave-one-out sensitivity analysis for
'C-X-C motif chemokine 10 levels' on 'Forearm'

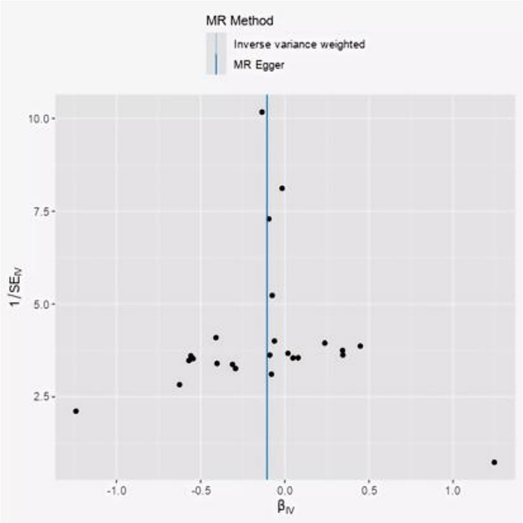

CXCL6

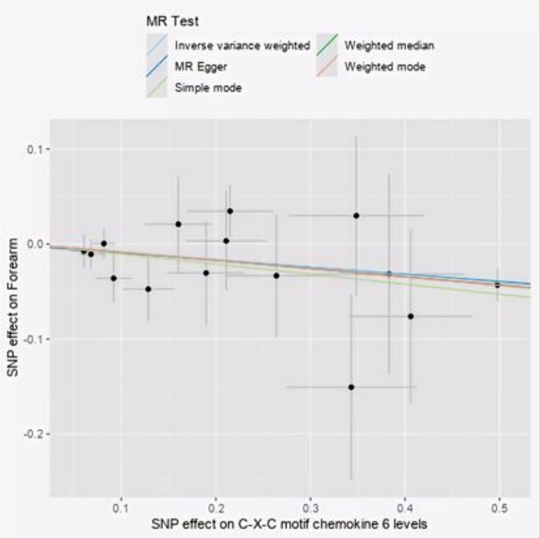

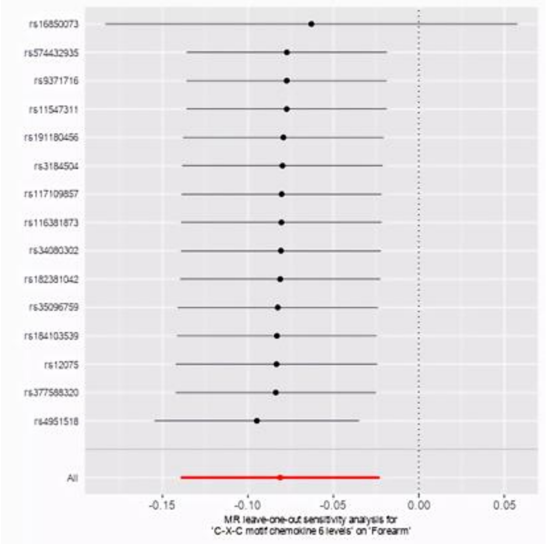

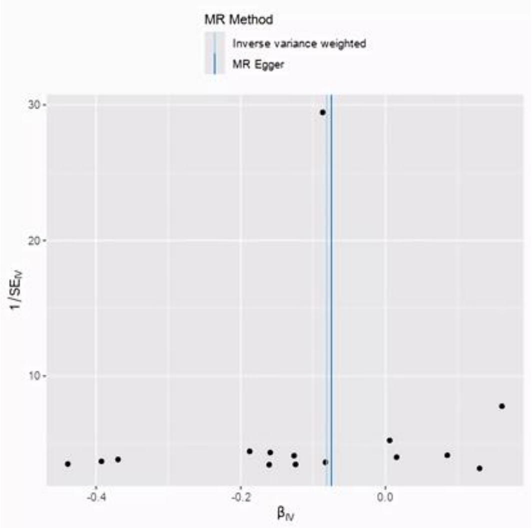

S100A12

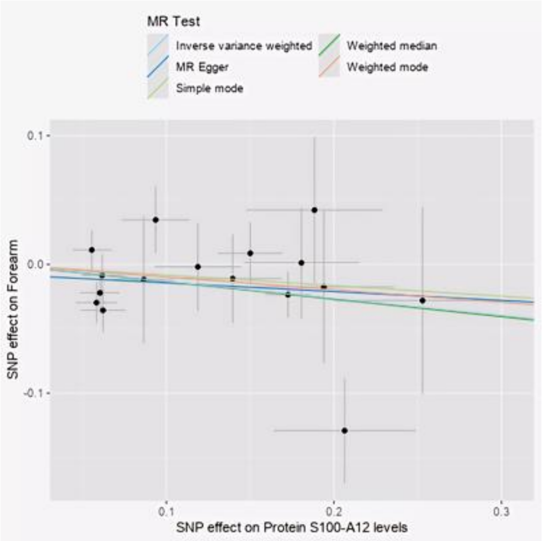
MR Test
Inverse variance weighted
MR Egger
Simple mode
Weighted median
Weighted mode
0.1
0.0
-0.1
SNP effect on Forearm
0.1
0.2
0.3
SNP effect on Protein S100-A12 levels

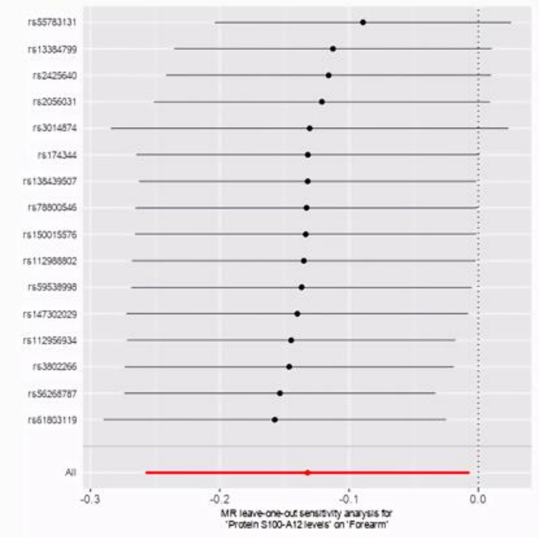
rs55783131
rs13384799
rs2425640
rs2056031
rs3014874
rs174344
rs138439507
rs78800546
rs150015576
rs112988802
rs59538998
rs147302029
rs112956934
rs3802266
rs56268787
rs61803119
All
-0.3
-0.2
-0.1
0.0
MR leave-one-out sensitivity analysis for
'Protein S100-A12 levels' on 'Forearm'

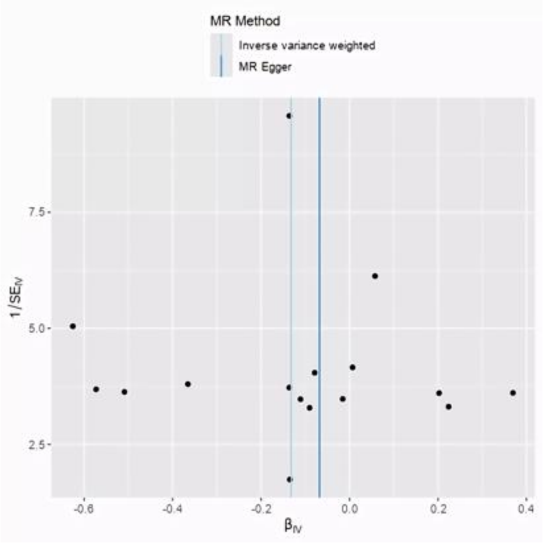
MR Method
Inverse variance weighted
MR Egger
7.5
5.0
2.5
$1/SE_{IV}$
-0.6
-0.4
-0.2
0.0
0.2
0.4
β_{IV}

FGF21

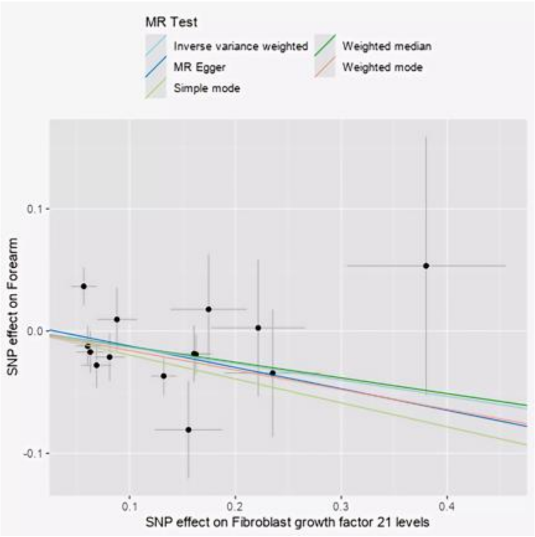
MR Test
Inverse variance weighted
MR Egger
Simple mode
Weighted median
Weighted mode
0.1
0.0
-0.1
SNP effect on Forearm
0.1
0.2
0.3
0.4
SNP effect on Fibroblast growth factor 21 levels

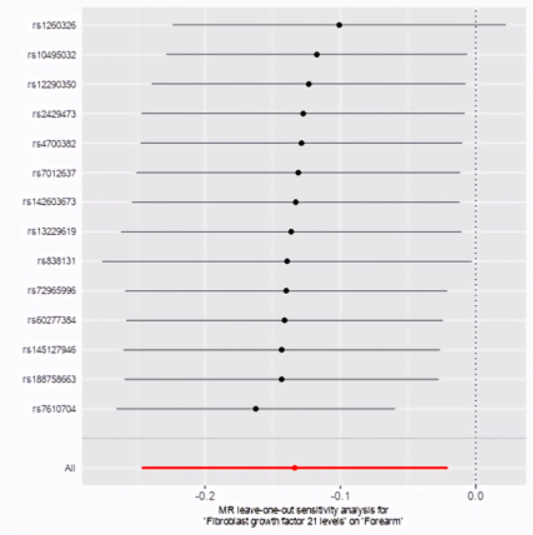
rs1260326
rs10495032
rs12290350
rs2429473
rs4700382
rs7012637
rs142603673
rs13229619
rs838131
rs72965996
rs60277384
rs145127946
rs188758663
rs7610704
All
-0.2
-0.1
0.0
MR leave-one-out sensitivity analysis for
'Fibroblast growth factor 21 levels' on 'Forearm'

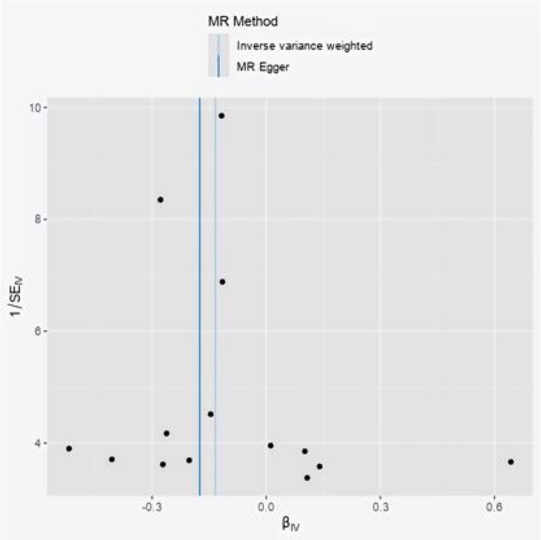
MR Method
Inverse variance weighted
MR Egger
1/SE_IV
β_IV
10
8
6
4
-0.3
0.0
0.3
0.6

LTA

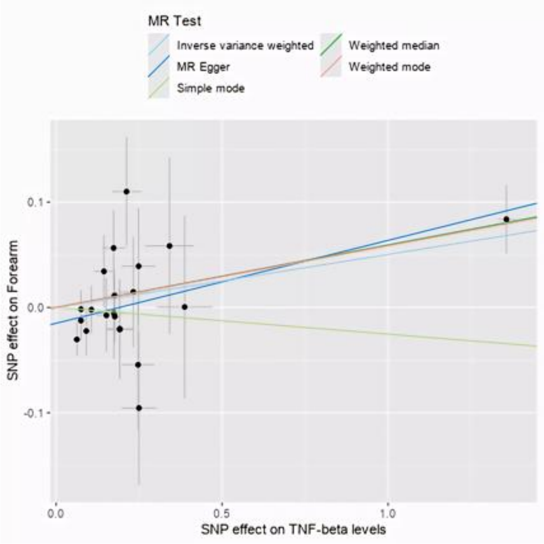
MR Test
Inverse variance weighted
MR Egger
Simple mode
Weighted median
Weighted mode
SNP effect on Forearm
SNP effect on TNF-beta levels
0.1
0.0
-0.1
0.0
0.5
1.0

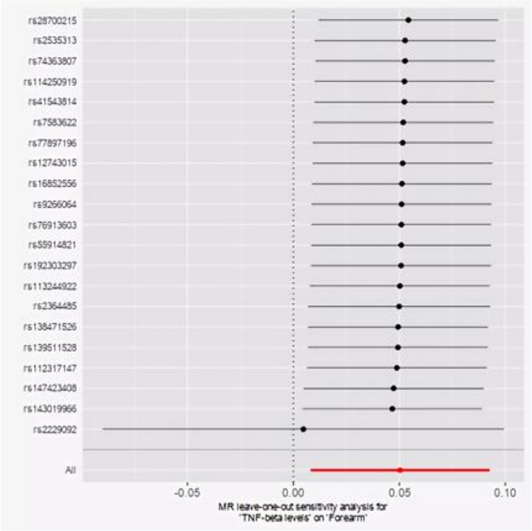
rs28700215
rs2535313
rs74363807
rs114250919
rs41543814
rs7583622
rs77897196
rs12743015
rs16852556
rs9266064
rs76913603
rs55914821
rs192303297
rs113244922
rs2364485
rs138471526
rs139511528
rs112317147
rs147423408
rs143019966
rs2229092
All
-0.05
0.00
0.05
0.10
MR leave-one-out sensitivity analysis for
'TNF-beta levels' on 'Forearm'

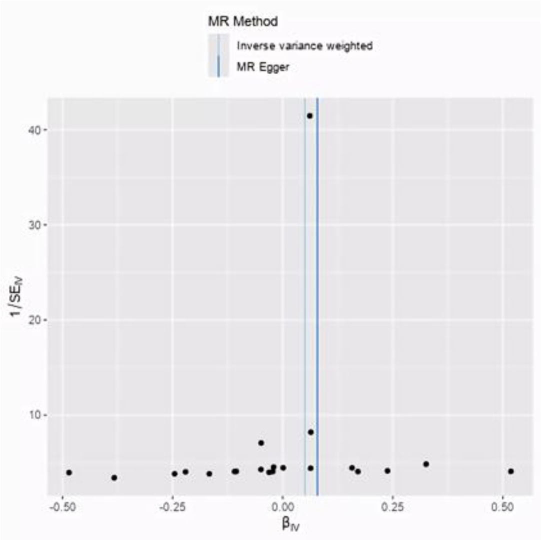
MR Method
Inverse variance weighted
MR Egger
1/SE_IV
β_IV
40
30
20
10
-0.50
-0.25
0.00
0.25
0.50

TNFSF14

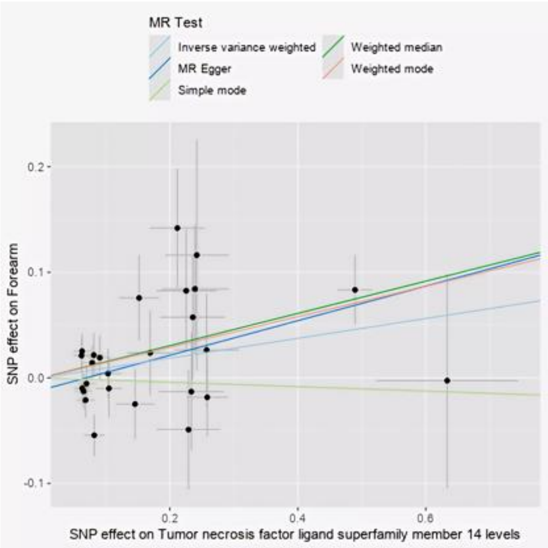

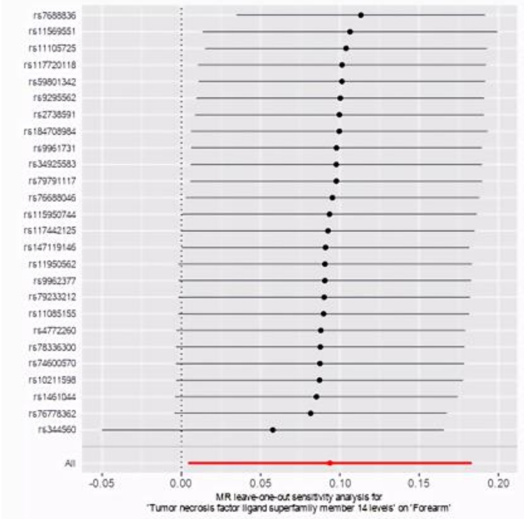

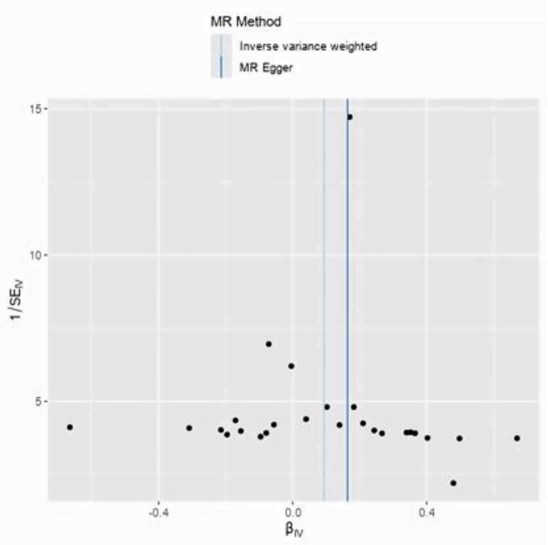

TSLP

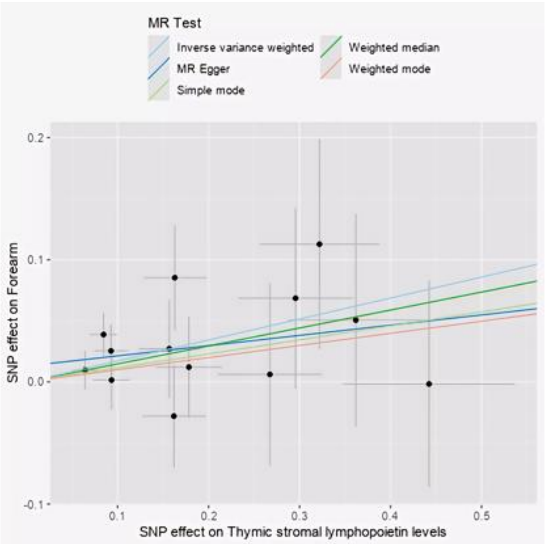

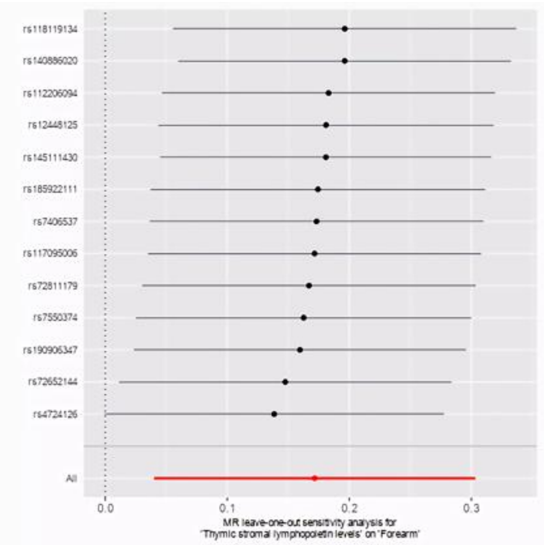

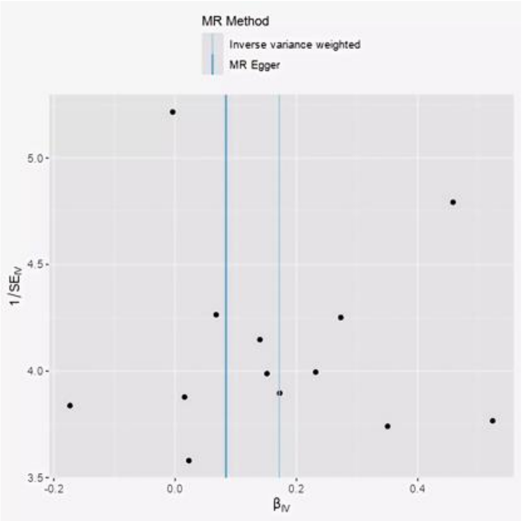

Results of FN

AXIN1

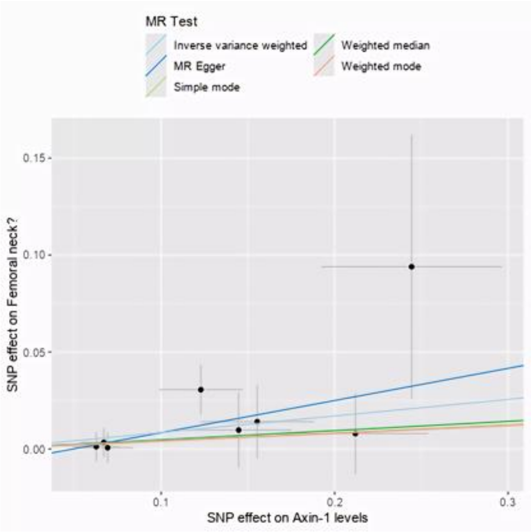

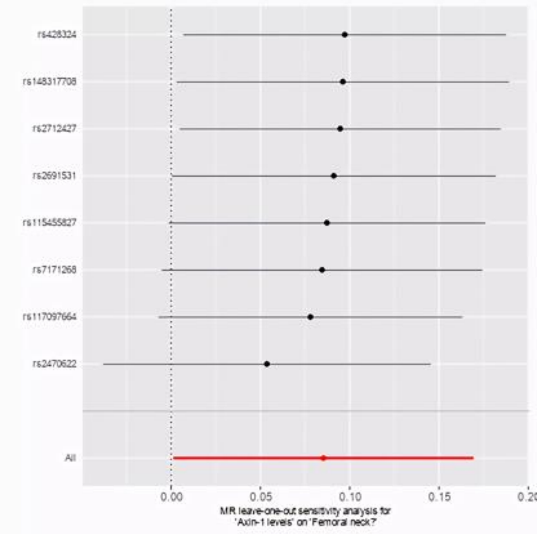

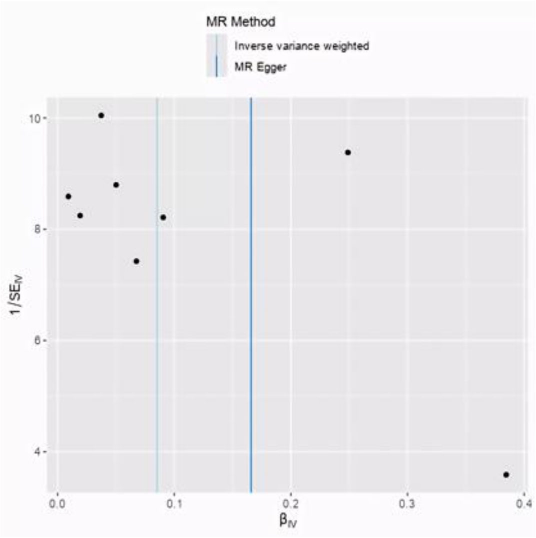

CXCL5

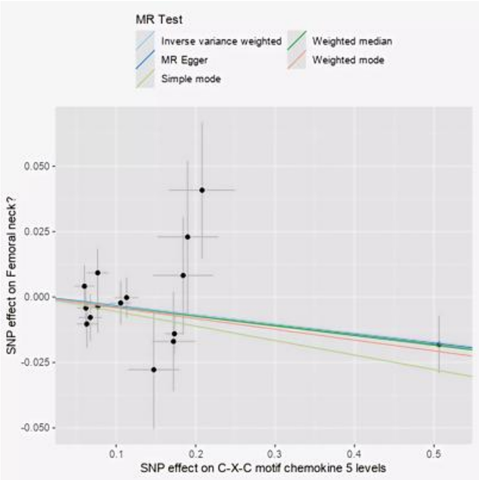
MR Test
Inverse variance weighted
MR Egger
Simple mode
Weighted median
Weighted mode
SNP effect on Femoral neck?
SNP effect on C-X-C motif chemokine 5 levels

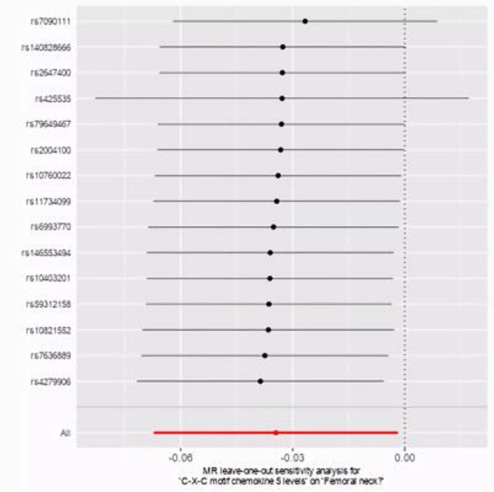
All
MR leave-one-out sensitivity analysis for
'C-X-C motif chemokine 5 levels' on 'Femoral neck?'

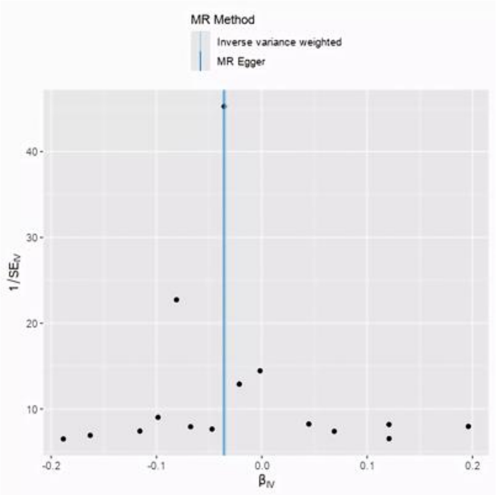
MR Method
Inverse variance weighted
MR Egger
$1/SE_{IV}$
β_{IV}

CST5

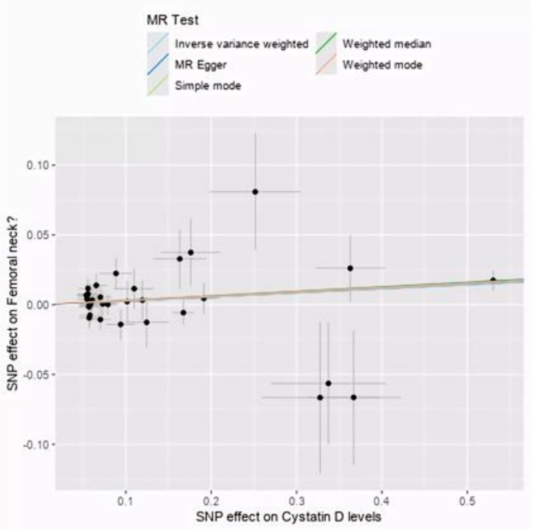
MR Test
Inverse variance weighted
MR Egger
Simple mode
Weighted median
Weighted mode
SNP effect on Femoral neck?
SNP effect on Cystatin D levels

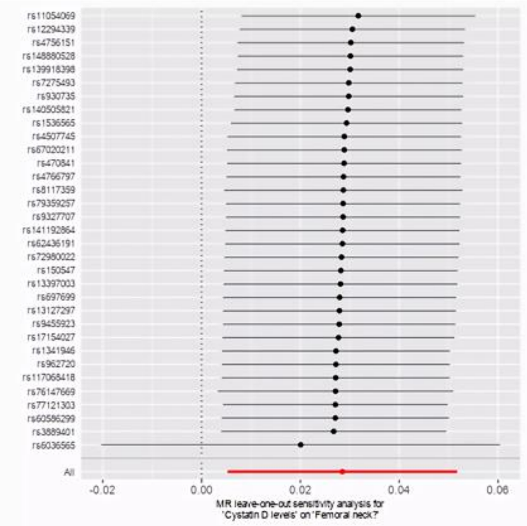
All
MR leave-one-out sensitivity analysis for
'Cystatin D levels' on 'Femoral neck?'

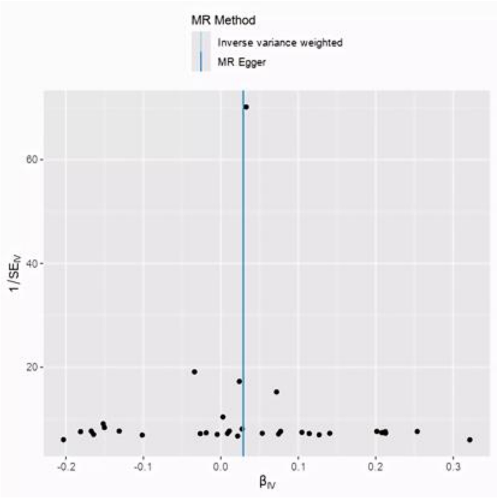

Oncostatin-M

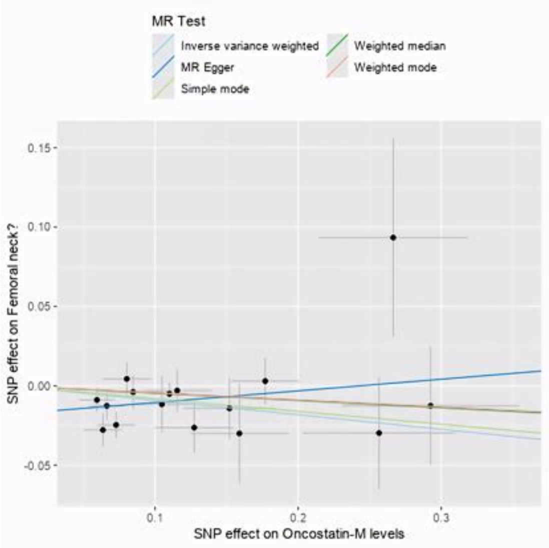

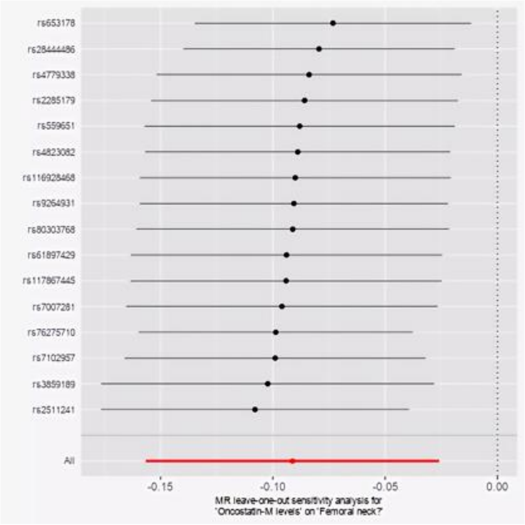

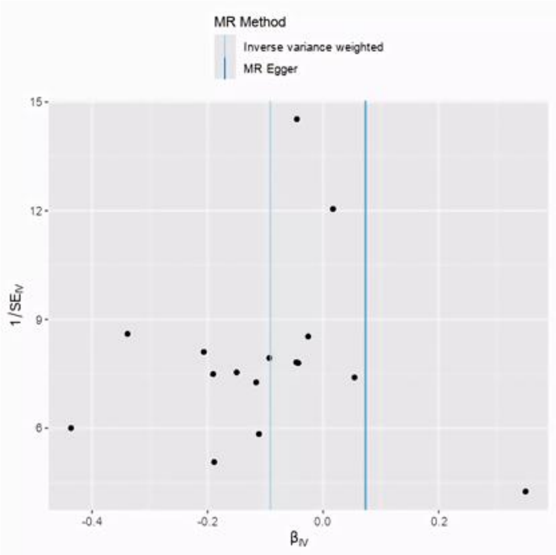

Results of HB

CCL4

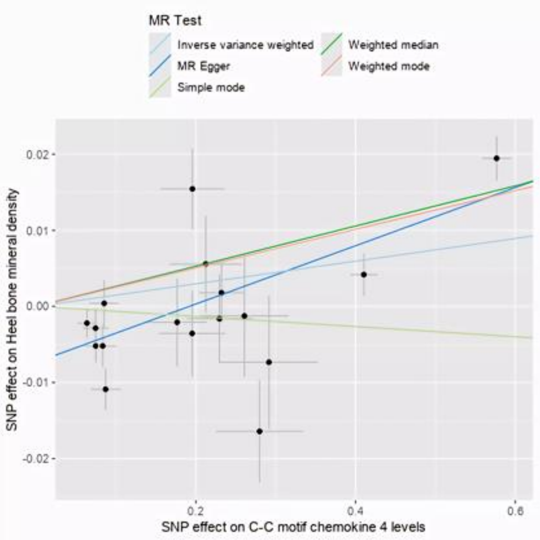

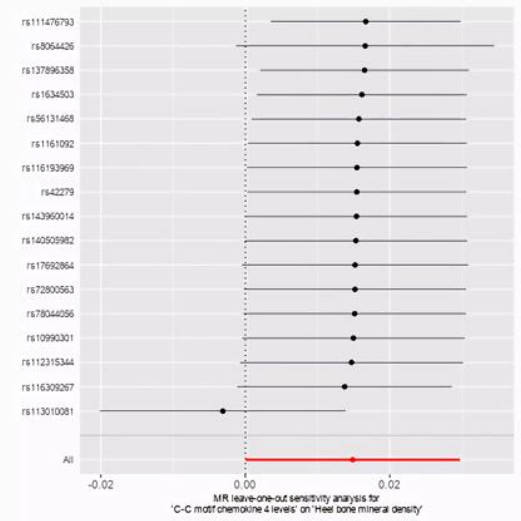

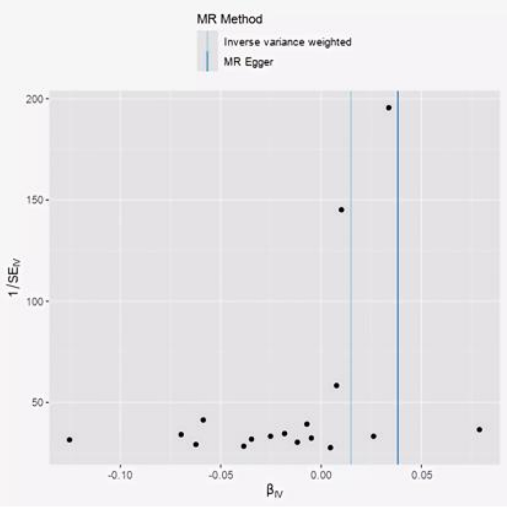

DNER

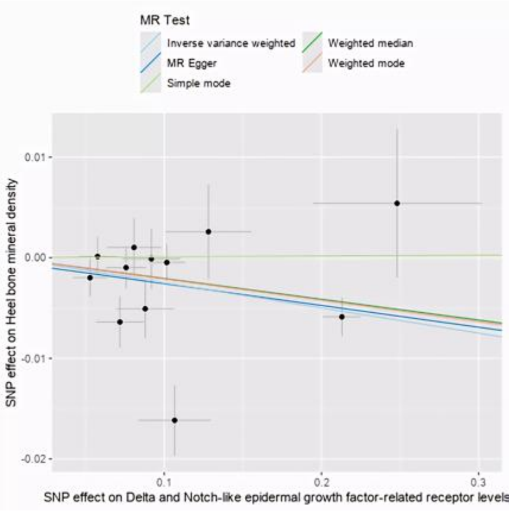

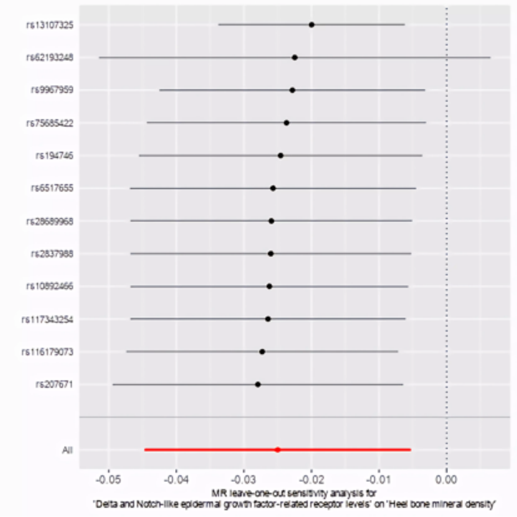

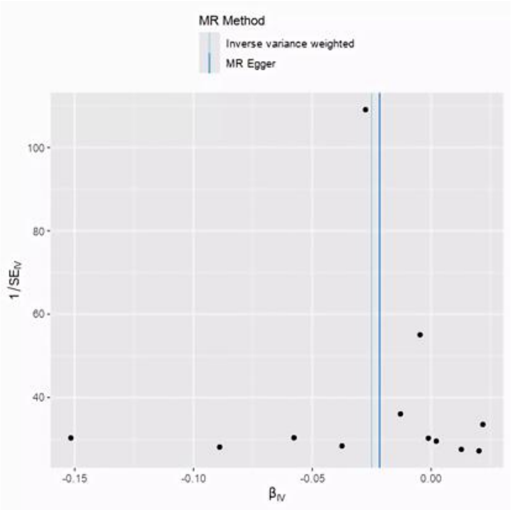
MR Method
Inverse variance weighted
MR Egger
100
80
60
40
1/SE_IV
-0.15
-0.10
-0.05
0.00
β_IV

IL15RA

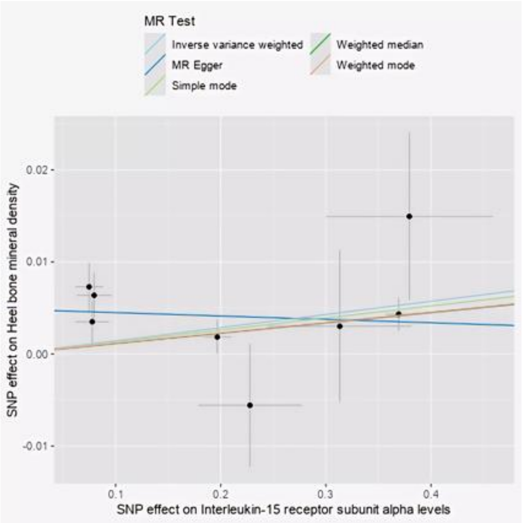
MR Test
Inverse variance weighted
MR Egger
Simple mode
Weighted median
Weighted mode
0.02
0.01
0.00
-0.01
SNP effect on Heel bone mineral density
0.1
0.2
0.3
0.4
SNP effect on Interleukin-15 receptor subunit alpha levels

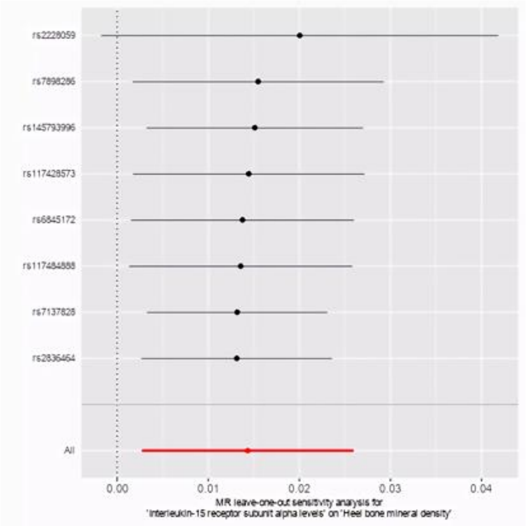
rs2228059
rs7898286
rs145793996
rs117428573
rs6845172
rs117484888
rs7137828
rs2836464
All
0.00
0.01
0.02
0.03
0.04
MR leave-one-out sensitivity analysis for
'Interleukin-15 receptor subunit alpha levels' on 'Heel bone mineral density'

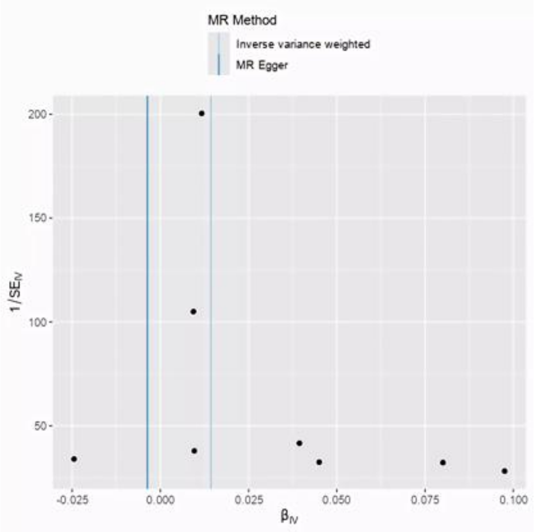
MR Method
Inverse variance weighted
MR Egger
200
150
100
50
1/SE_IV
-0.025
0.000
0.025
0.050
0.075
0.100
β_IV

LIFR

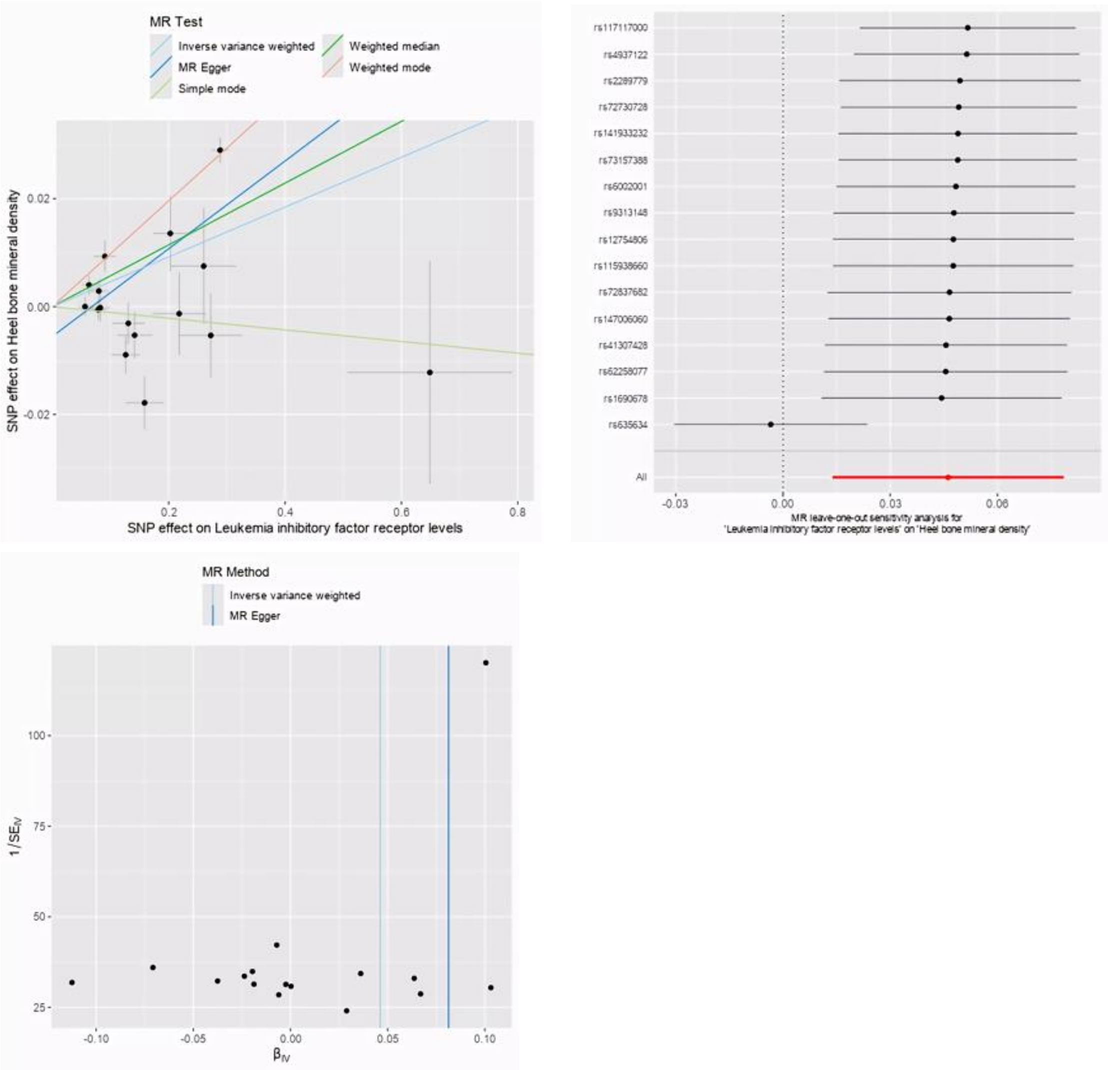
MR Test
Inverse variance weighted
MR Egger
Simple mode
Weighted median
Weighted mode
SNP effect on Heel bone mineral density
SNP effect on Leukemia inhibitory factor receptor levels
MR leave-one-out sensitivity analysis for
'Leukemia inhibitory factor receptor levels' on 'Heel bone mineral density'
All
MR Method
Inverse variance weighted
MR Egger
$1/SE_{IV}$
β_{IV}

SCF

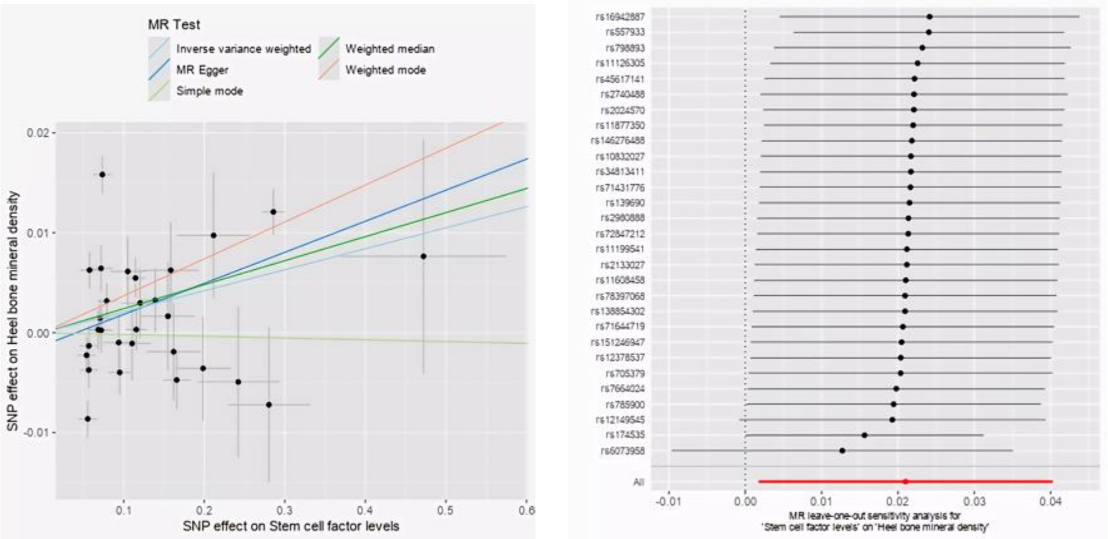
MR Test
Inverse variance weighted
MR Egger
Simple mode
Weighted median
Weighted mode
SNP effect on Heel bone mineral density
SNP effect on Stem cell factor levels
MR leave-one-out sensitivity analysis for
'Stem cell factor levels' on 'Heel bone mineral density'
All

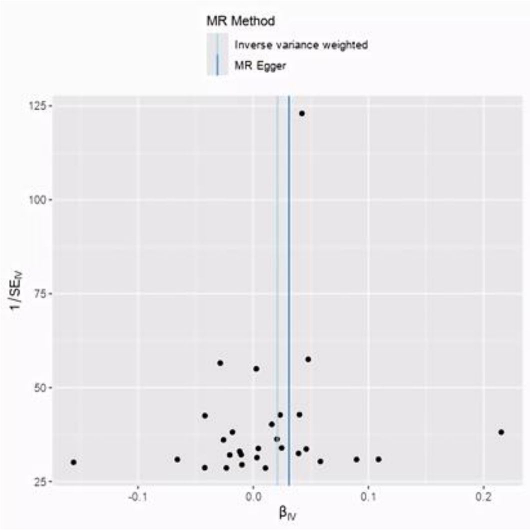
MR Method
Inverse variance weighted
MR Egger
125
100
75
50
25
1/SE_IV
-0.1
0.0
0.1
0.2
β_IV

TGFA

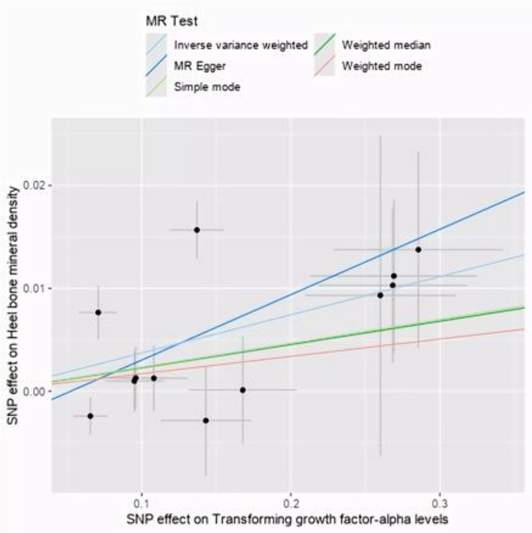
MR Test
Inverse variance weighted
MR Egger
Simple mode
Weighted median
Weighted mode
0.02
0.01
0.00
SNP effect on Heel bone mineral density
0.1
0.2
0.3
SNP effect on Transforming growth factor-alpha levels

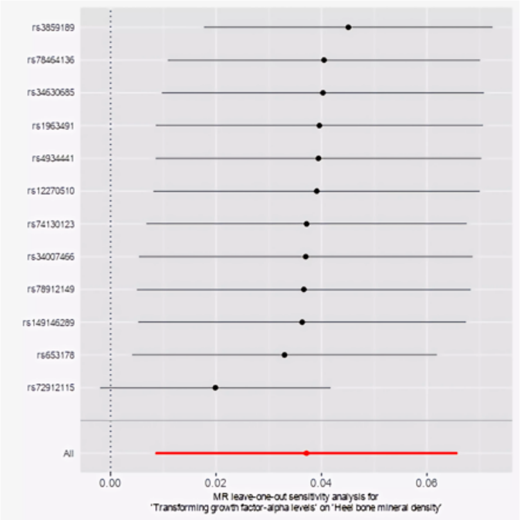
rs3859189
rs78464136
rs34630685
rs1963491
rs4934441
rs12270510
rs74130123
rs34007466
rs78912149
rs149146289
rs653178
rs72912115
All
0.00
0.02
0.04
0.06
MR leave-one-out sensitivity analysis for
'Transforming growth factor-alpha levels' on 'Heel bone mineral density'

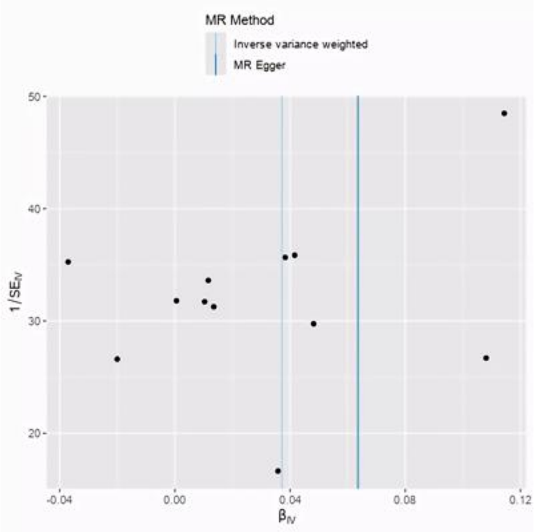
MR Method
Inverse variance weighted
MR Egger
50
40
30
20
1/SE_IV
-0.04
0.00
0.04
0.08
0.12
β_IV

VEGFA

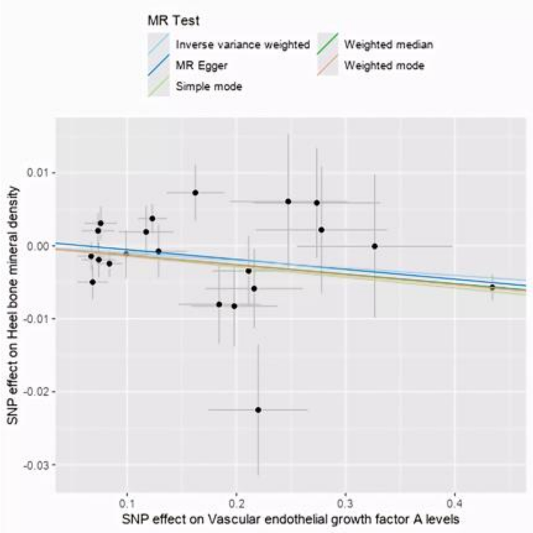

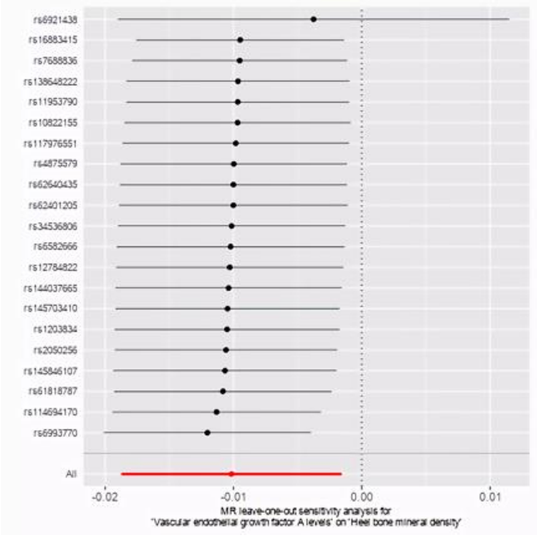

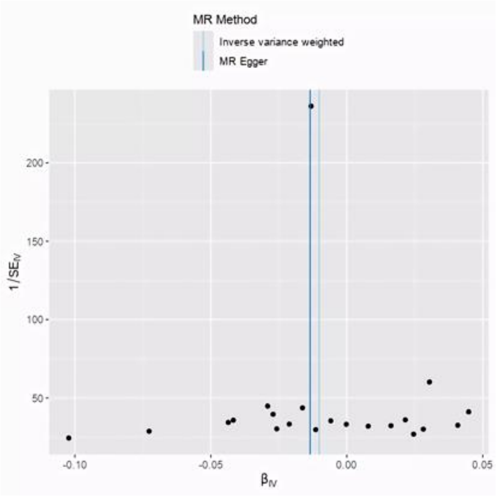

Results of LS

CXCL5

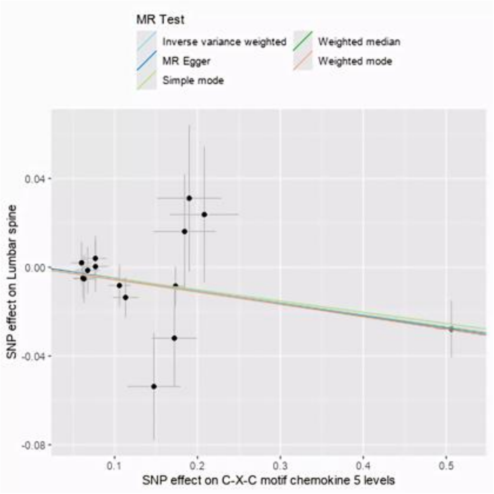

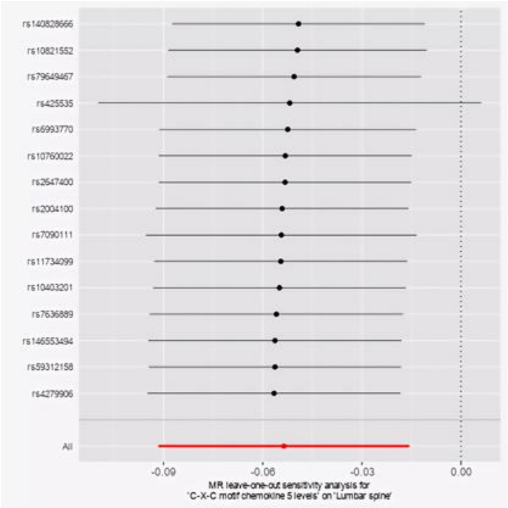

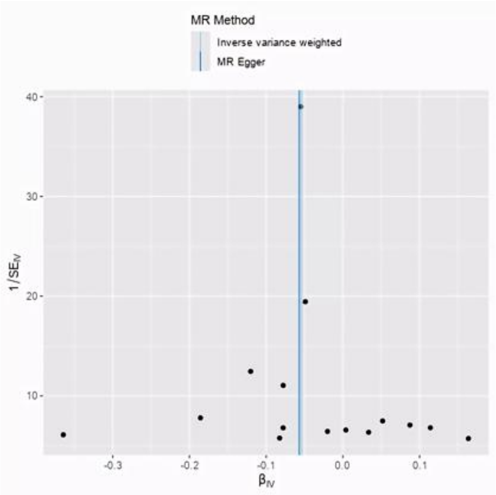
MR Method
Inverse variance weighted
MR Egger
1/SE_IV
β_IV

FGF19

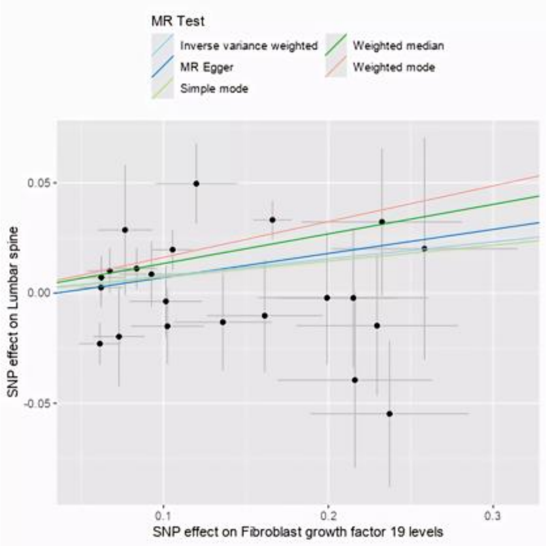
MR Test
Inverse variance weighted
MR Egger
Simple mode
Weighted median
Weighted mode
SNP effect on Lumbar spine
SNP effect on Fibroblast growth factor 19 levels

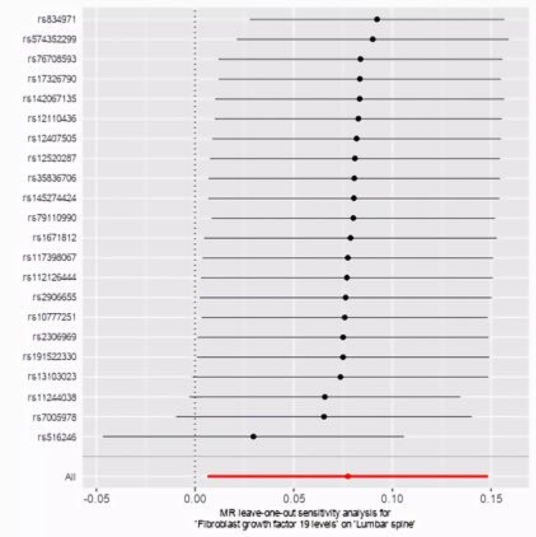
All
MR leave-one-out sensitivity analysis for
'Fibroblast growth factor 19 levels' on 'Lumbar spine'

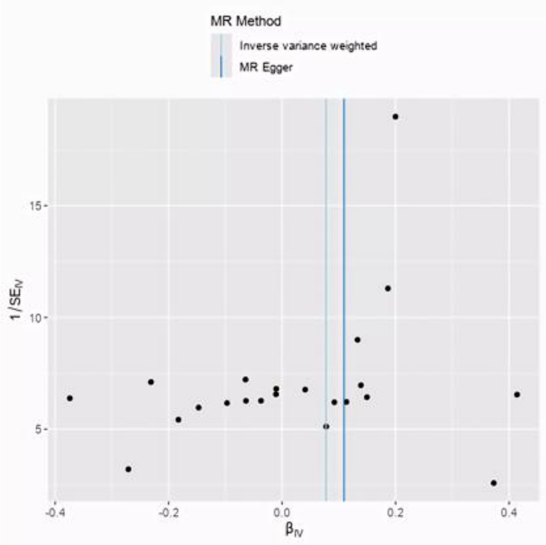
MR Method
Inverse variance weighted
MR Egger
1/SE_IV
β_IV

GDNF

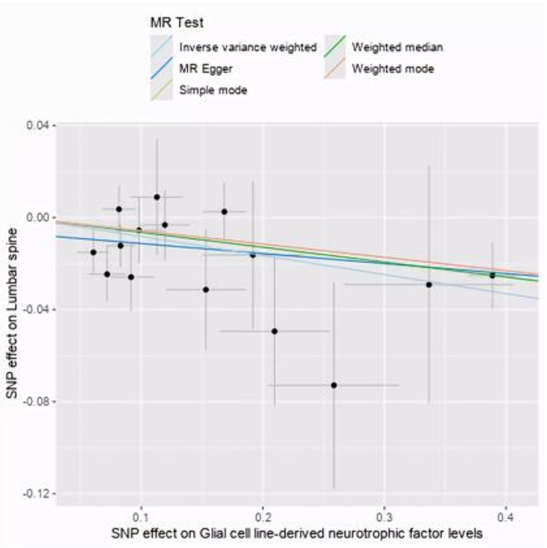
MR Test
Inverse variance weighted
MR Egger
Simple mode
Weighted median
Weighted mode
SNP effect on Lumbar spine
SNP effect on Glial cell line-derived neurotrophic factor levels

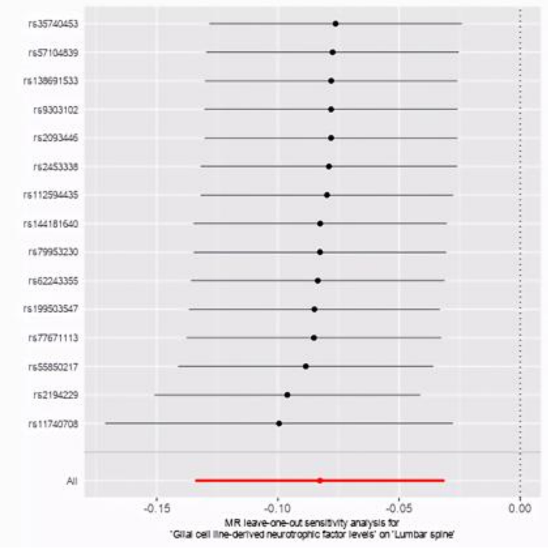
All
MR leave-one-out sensitivity analysis for
'Glial cell line-derived neurotrophic factor levels' on 'Lumbar spine'

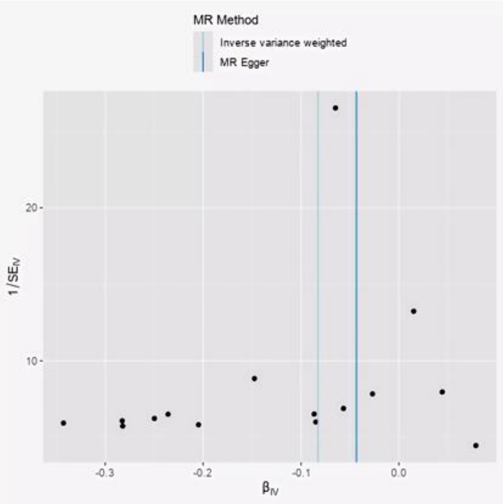
MR Method
Inverse variance weighted
MR Egger
1/SE_IV
β_IV

IL5

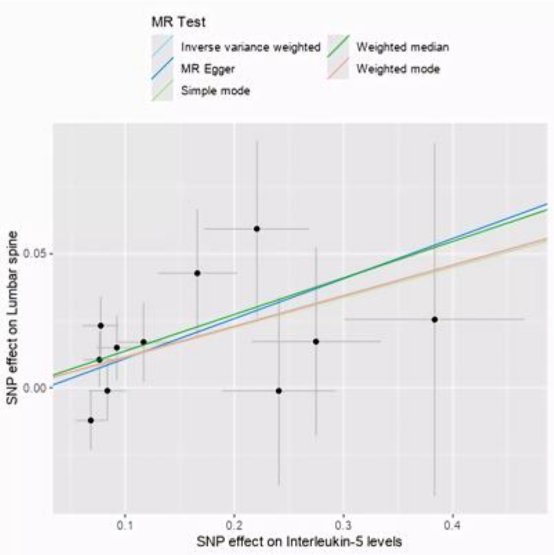
MR Test
Inverse variance weighted
MR Egger
Simple mode
Weighted median
Weighted mode
SNP effect on Lumbar spine
SNP effect on Interleukin-5 levels

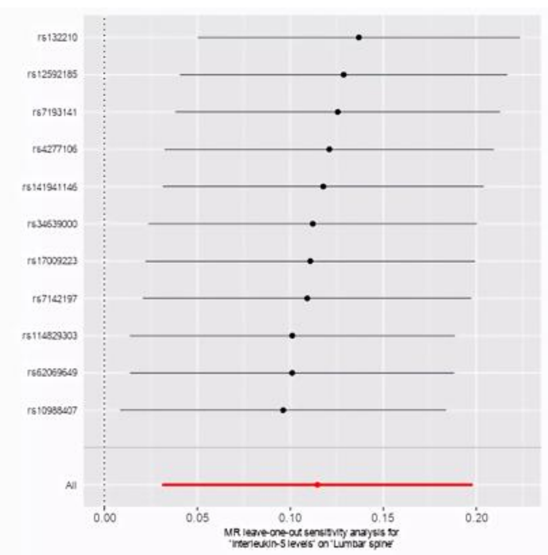
All
MR leave-one-out sensitivity analysis for
'Interleukin-5 levels' on 'Lumbar spine'

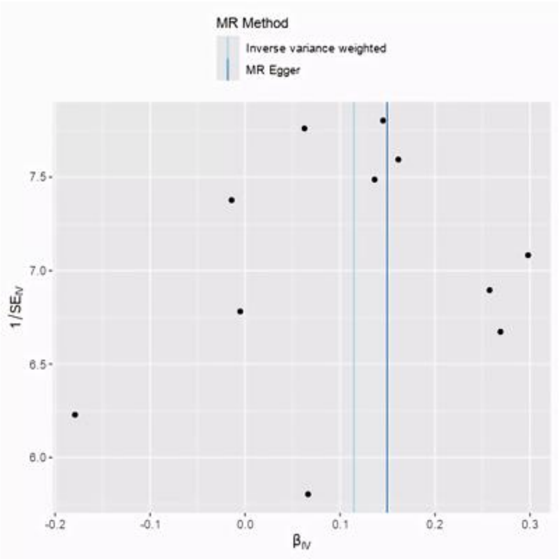

LIFR

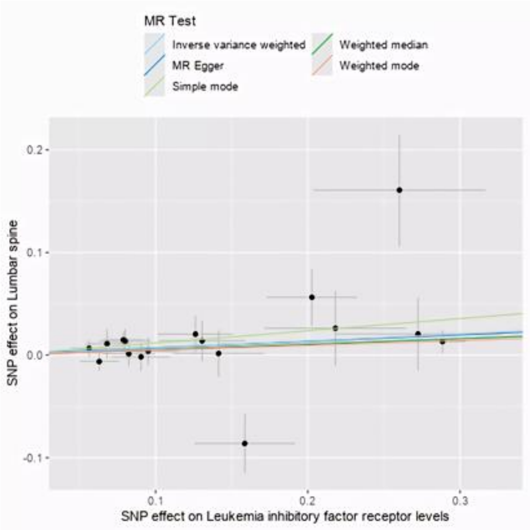

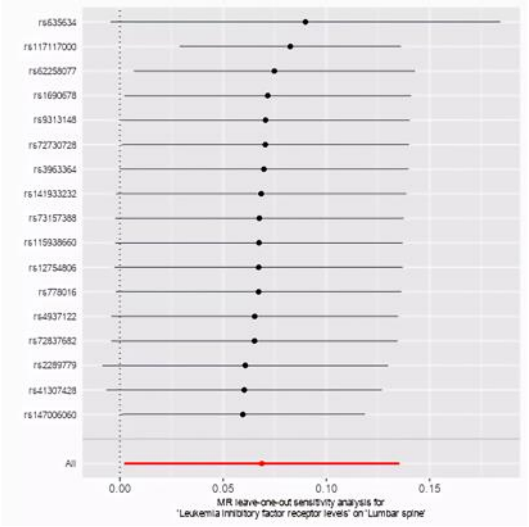

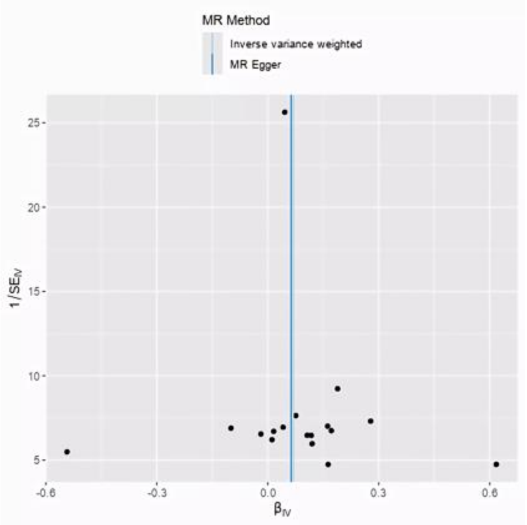

Oncostatin-M

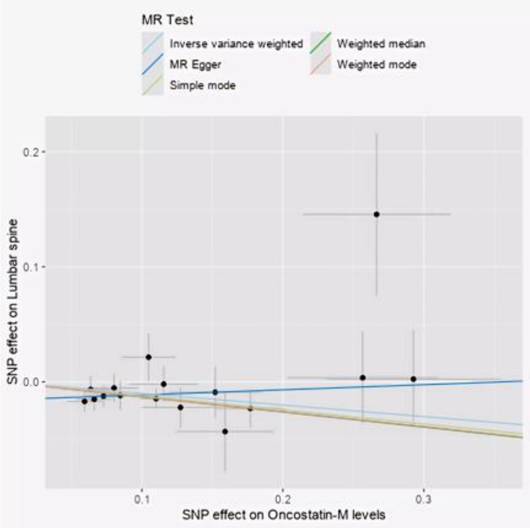
MR Test
Inverse variance weighted
MR Egger
Simple mode
Weighted median
Weighted mode
0.2
0.1
0.0
SNP effect on Lumbar spine
0.1
0.2
0.3
SNP effect on Oncostatin-M levels

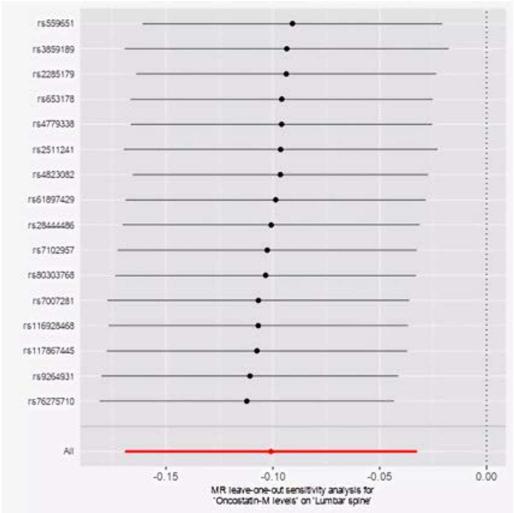
rs559651
rs3859189
rs2285179
rs653178
rs4779338
rs2511241
rs4823082
rs61897429
rs28444486
rs7102957
rs80303768
rs7007281
rs116928468
rs117867445
rs9264931
rs76275710
All
-0.15
-0.10
-0.05
0.00
MR leave-one-out sensitivity analysis for
'Oncostatin-M levels' on 'Lumbar spine'

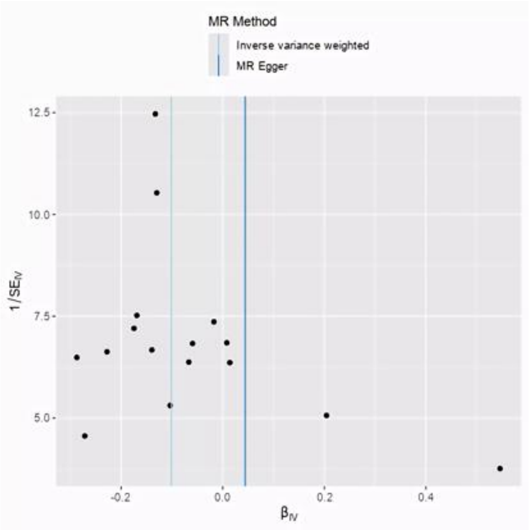
MR Method
Inverse variance weighted
MR Egger
12.5
10.0
7.5
5.0
1/SE$_{IV}$
-0.2
0.0
0.2
0.4
β$_{IV}$

TRAIL

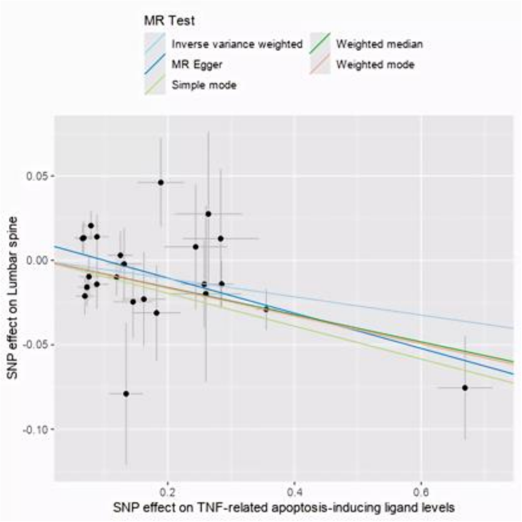
MR Test
Inverse variance weighted
MR Egger
Simple mode
Weighted median
Weighted mode
0.05
0.00
-0.05
-0.10
SNP effect on Lumbar spine
0.2
0.4
0.6
SNP effect on TNF-related apoptosis-inducing ligand levels

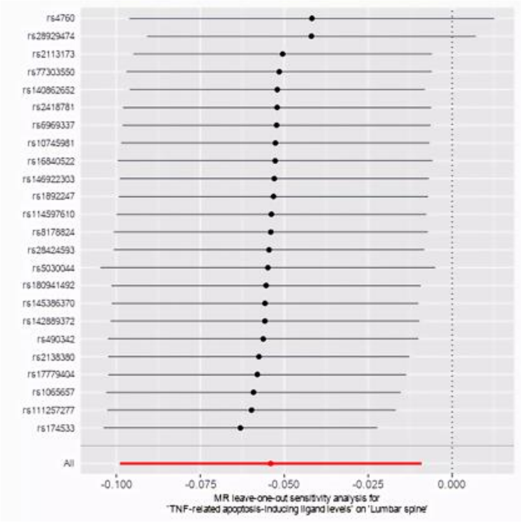
rs4760
rs28929474
rs2113173
rs77303550
rs140862652
rs2418781
rs6969337
rs10745981
rs16840522
rs146922303
rs1892247
rs114597610
rs8178824
rs28424593
rs5030044
rs180941492
rs145386370
rs142889372
rs490342
rs2138380
rs17779404
rs1065657
rs111257277
rs174533
All
-0.100
-0.075
-0.050
-0.025
0.000
MR leave-one-out sensitivity analysis for
'TNF-related apoptosis-inducing ligand levels' on 'Lumbar spine'

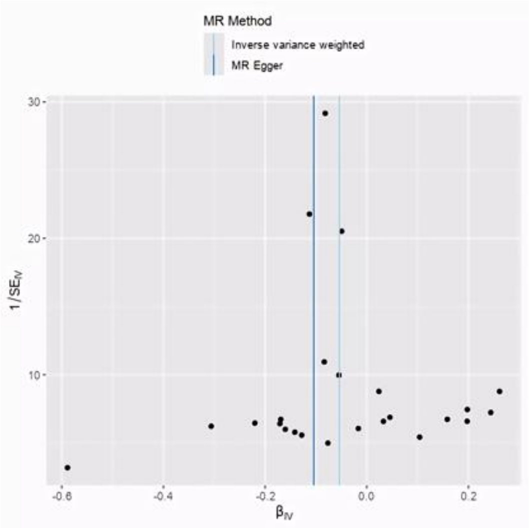

Results of Fracture

CXCL11

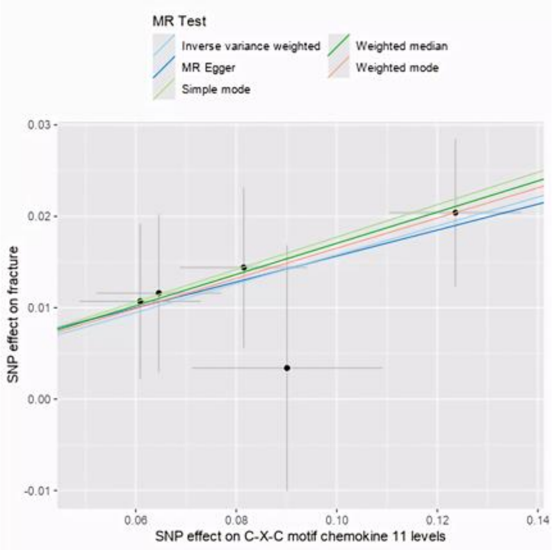

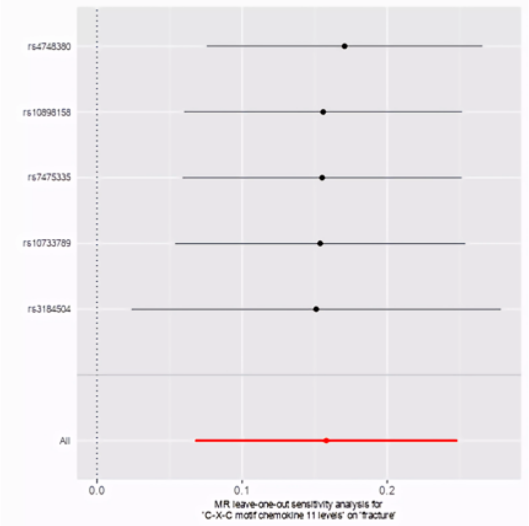

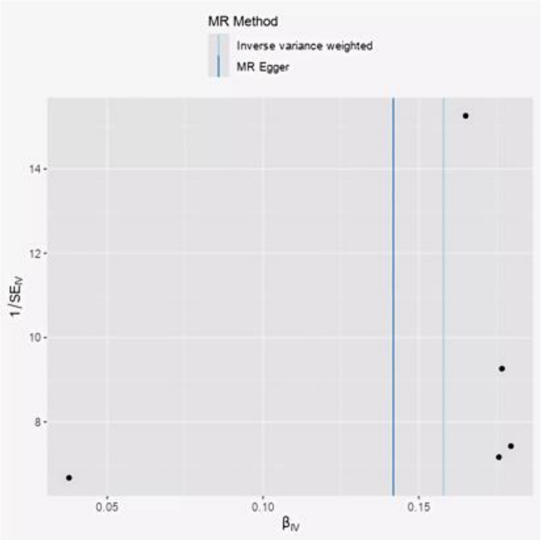

FGF19

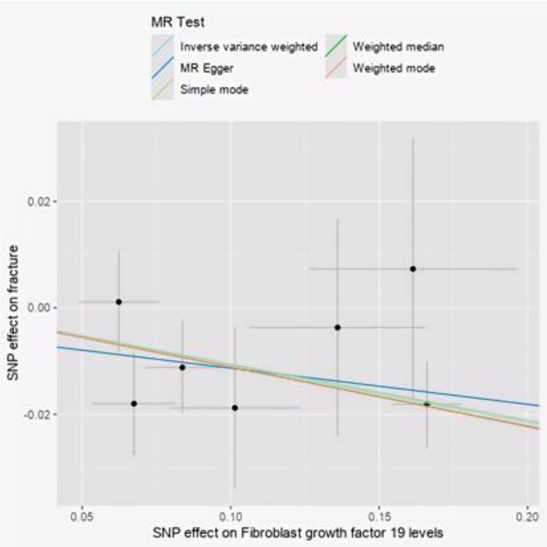
MR Test
Inverse variance weighted
MR Egger
Simple mode
Weighted median
Weighted mode
SNP effect on fracture
SNP effect on Fibroblast growth factor 19 levels

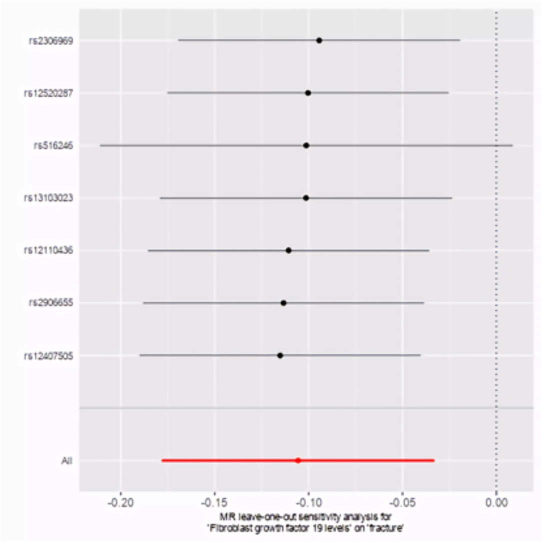
rs2306969
rs12520287
rs516246
rs13103023
rs12110436
rs2906655
rs12407505
All
MR leave-one-out sensitivity analysis for
'Fibroblast growth factor 19 levels' on 'fracture'

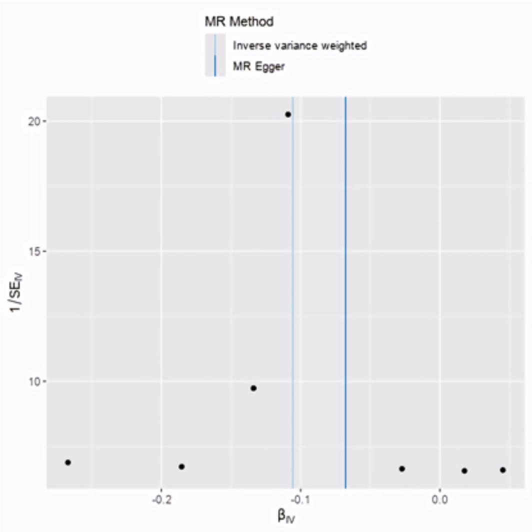
MR Method
Inverse variance weighted
MR Egger
1/SE_IV
β_IV

OPG

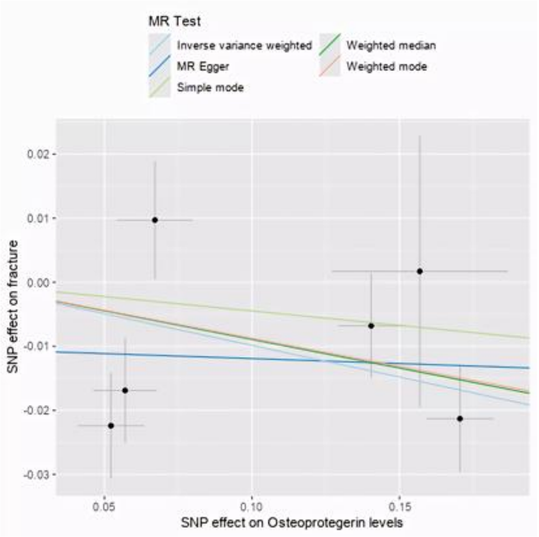
MR Test
Inverse variance weighted
MR Egger
Simple mode
Weighted median
Weighted mode
SNP effect on fracture
SNP effect on Osteoprotegerin levels

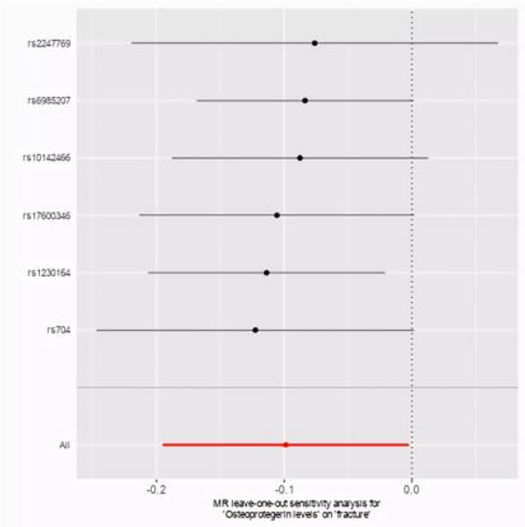
rs2247769
rs6985207
rs10142466
rs17600346
rs1230164
rs704
All
MR leave-one-out sensitivity analysis for
'Osteoprotegerin levels' on 'fracture'

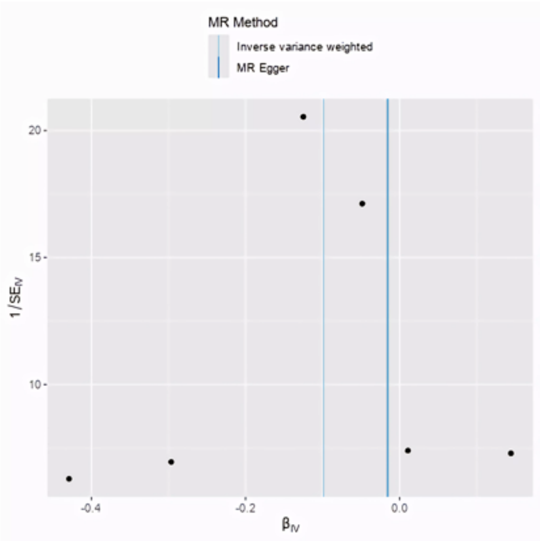
MR Method
Inverse variance weighted
MR Egger
1/SE$_{IV}$
β_{IV}

SLAM

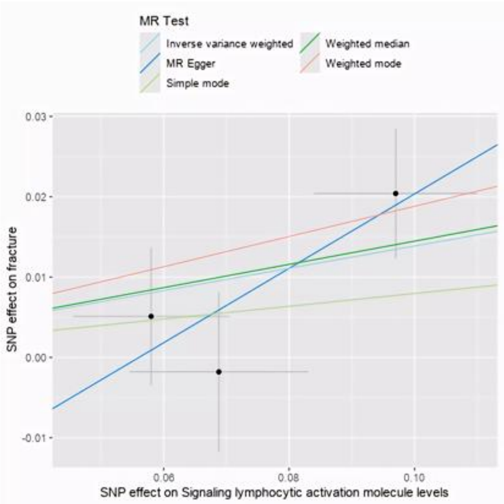
MR Test
Inverse variance weighted
MR Egger
Simple mode
Weighted median
Weighted mode
SNP effect on fracture
SNP effect on Signaling lymphocytic activation molecule levels

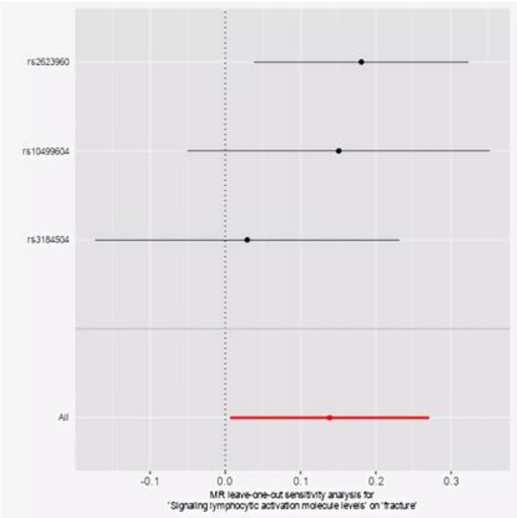
rs2623960
rs10499604
rs3184504
All
MR leave-one-out sensitivity analysis for
'Signaling lymphocytic activation molecule levels' on 'fracture'

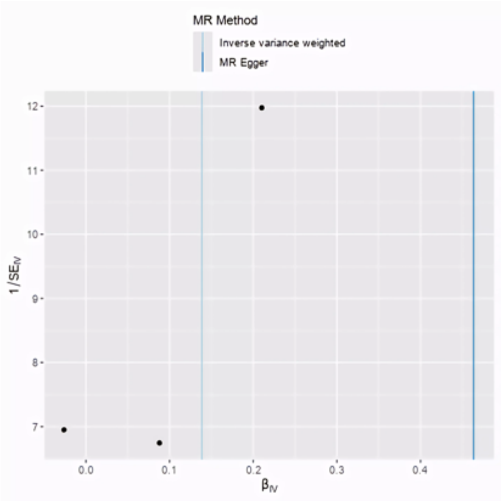
MR Method
Inverse variance weighted
MR Egger
1/SE$_{IV}$
β_{IV}

Results of ALM

GDNF

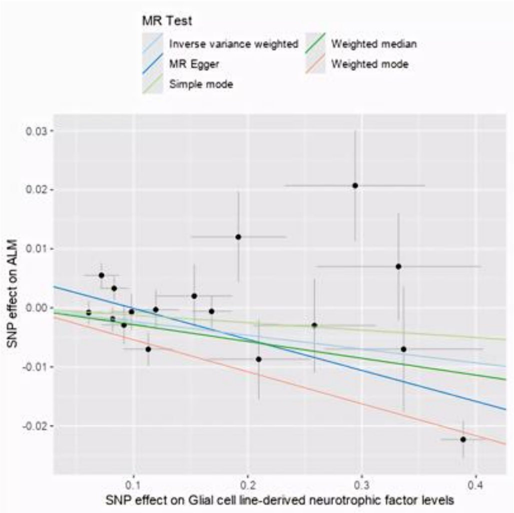

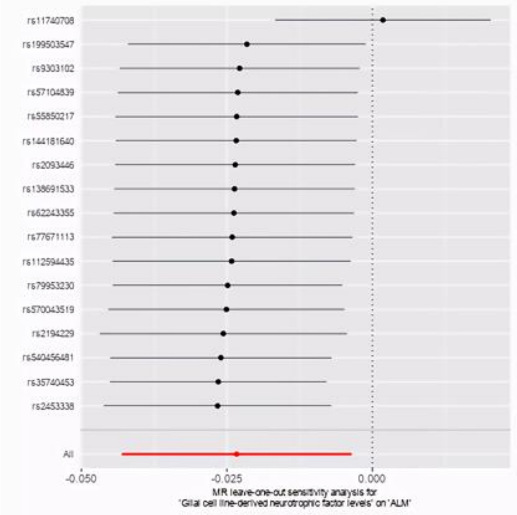

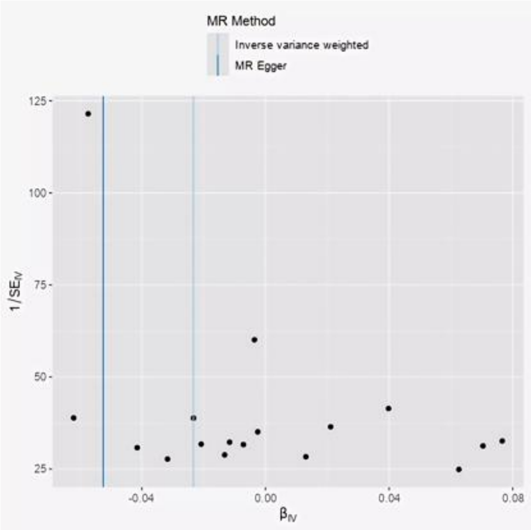

HGF

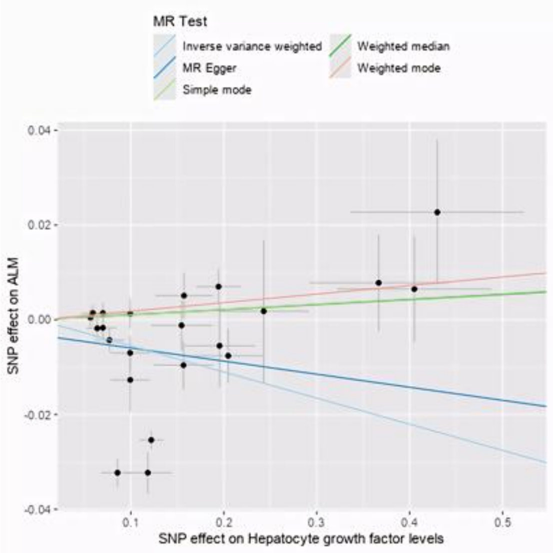

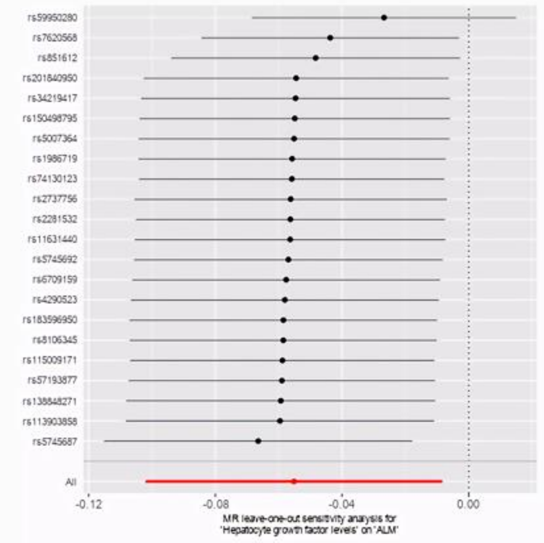

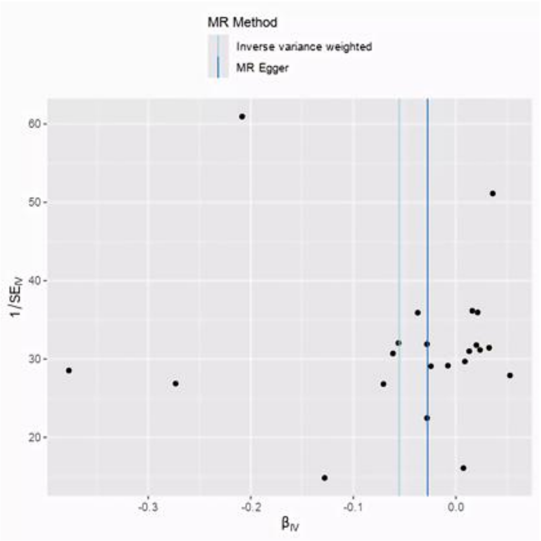
MR Method
Inverse variance weighted
MR Egger
1/SE_IV
β_IV
60
50
40
30
20
-0.3
-0.2
-0.1
0.0

IL1A

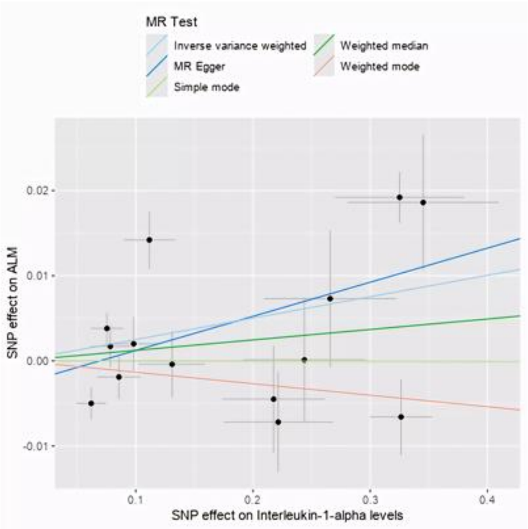
MR Test
Inverse variance weighted
MR Egger
Simple mode
Weighted median
Weighted mode
SNP effect on ALM
SNP effect on Interleukin-1-alpha levels
0.02
0.01
0.00
-0.01
0.1
0.2
0.3
0.4

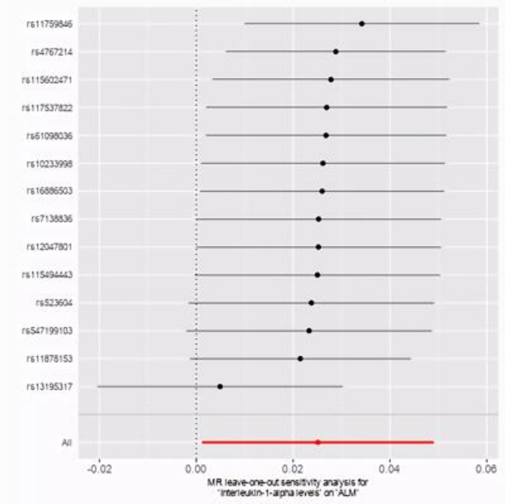
rs11759846
rs4767214
rs115602471
rs117537822
rs61098036
rs10233998
rs16886503
rs7138836
rs12047801
rs115494443
rs523604
rs547199103
rs11878153
rs13195317
All
-0.02
0.00
0.02
0.04
0.06
MR leave-one-out sensitivity analysis for
"Interleukin-1-alpha levels" on "ALM"

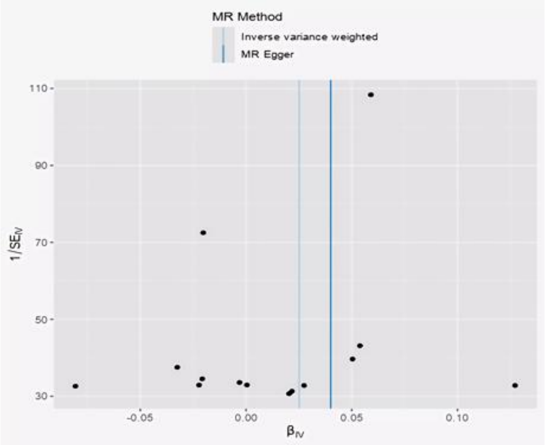
MR Method
Inverse variance weighted
MR Egger
1/SE_IV
β_IV
110
90
70
50
30
-0.05
0.00
0.05
0.10

IL2

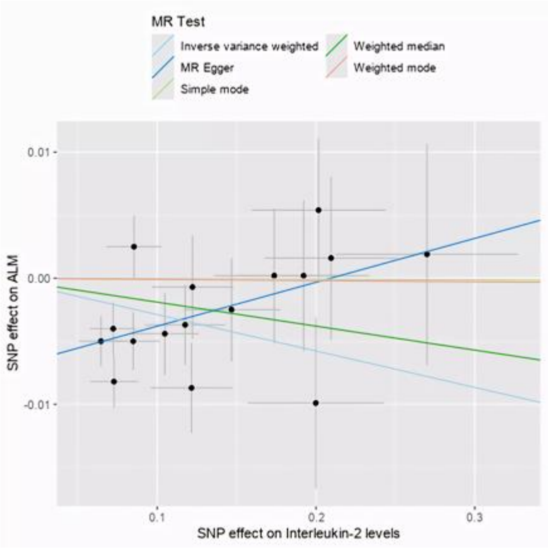
MR Test
Inverse variance weighted
MR Egger
Simple mode
Weighted median
Weighted mode
SNP effect on ALM
SNP effect on Interleukin-2 levels

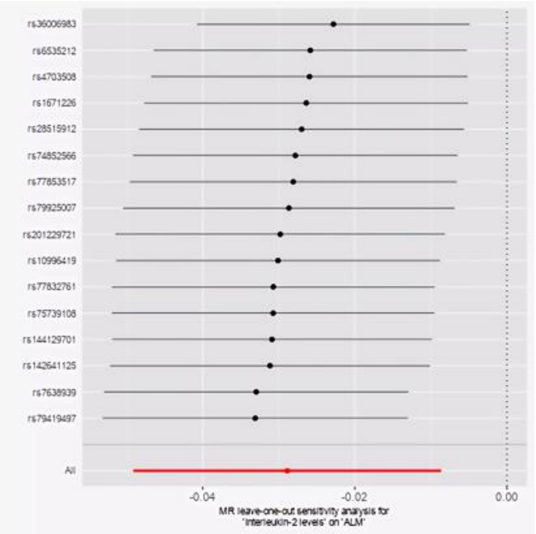
rs36006983
rs6535212
rs4703508
rs1671226
rs28515912
rs74852566
rs77853517
rs79925007
rs201229721
rs10996419
rs77832761
rs75739108
rs144129701
rs142641125
rs7638939
rs79419497
All
MR leave-one-out sensitivity analysis for
'Interleukin-2 levels' on 'ALM'

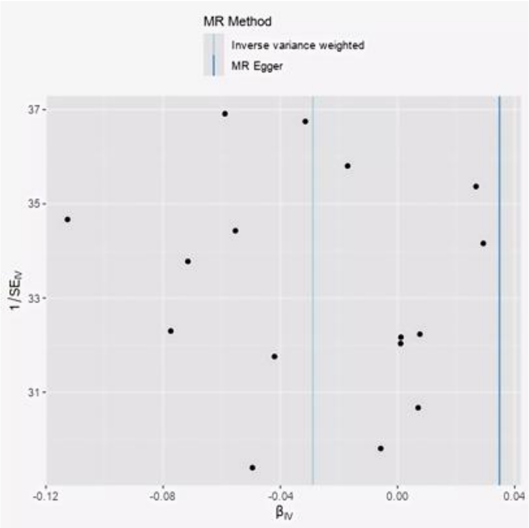
MR Method
Inverse variance weighted
MR Egger
1/SE$_{IV}$
β$_{IV}$

IL24

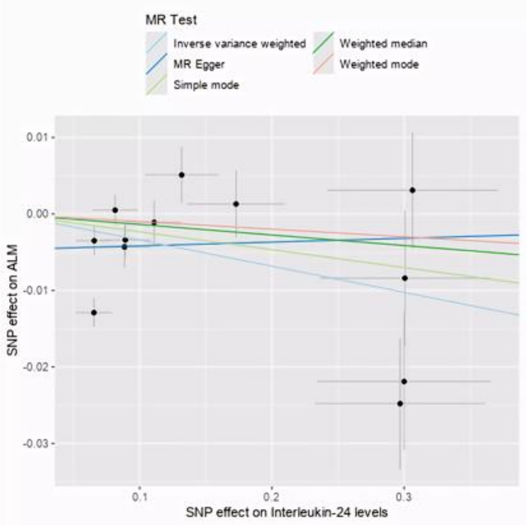
MR Test
Inverse variance weighted
MR Egger
Simple mode
Weighted median
Weighted mode
SNP effect on ALM
SNP effect on Interleukin-24 levels

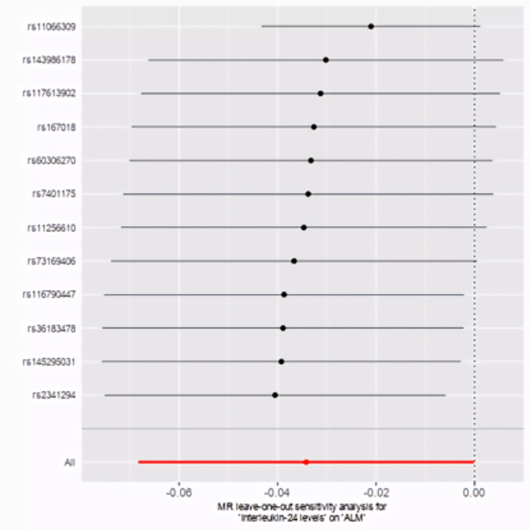
rs11066309
rs143986178
rs117613902
rs167018
rs60306270
rs7401175
rs11256610
rs73169406
rs116790447
rs36183478
rs145295031
rs2341294
All
MR leave-one-out sensitivity analysis for
'Interleukin-24 levels' on 'ALM'

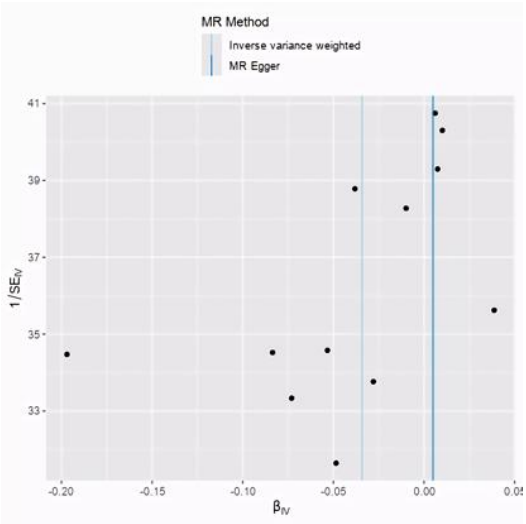
MR Method
Inverse variance weighted
MR Egger
41
39
37
35
33
1/SE$_{IV}$
-0.20
-0.15
-0.10
-0.05
0.00
0.05
β$_{IV}$

LTA

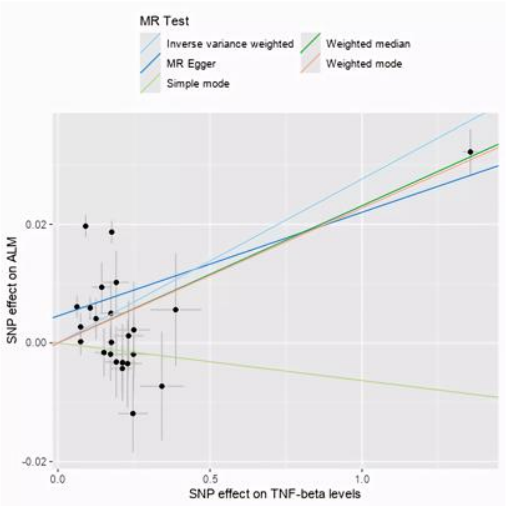
MR Test
Inverse variance weighted
MR Egger
Simple mode
Weighted median
Weighted mode
0.02
0.00
-0.02
SNP effect on ALM
0.0
0.5
1.0
SNP effect on TNF-beta levels

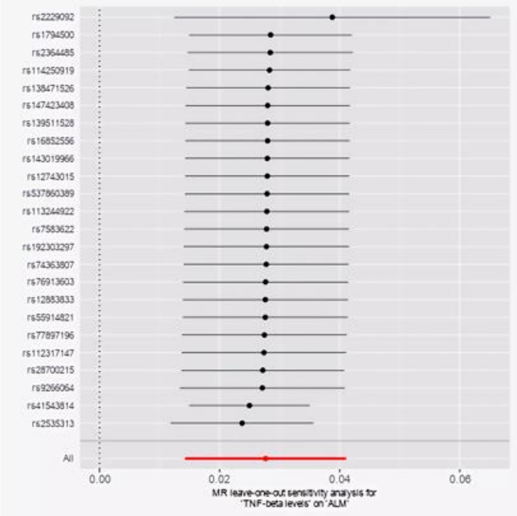
rs2229092
rs1794500
rs2364485
rs114250919
rs138471526
rs147423408
rs139511528
rs16852556
rs143019966
rs12743015
rs537860389
rs113244922
rs7583622
rs192303297
rs74363807
rs76913603
rs12883833
rs55914821
rs77897196
rs112317147
rs28700215
rs9266064
rs41543814
rs2535313
All
0.00
0.02
0.04
0.06
MR leave-one-out sensitivity analysis for
'TNF-beta levels' on 'ALM'

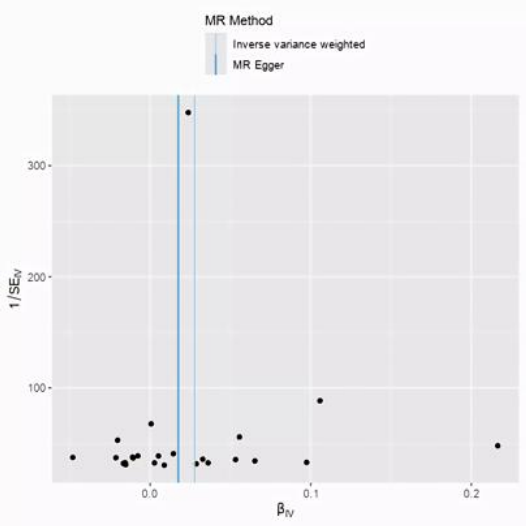
MR Method
Inverse variance weighted
MR Egger
300
200
100
1/SE$_{IV}$
0.0
0.1
0.2
β$_{IV}$

TNFSF12

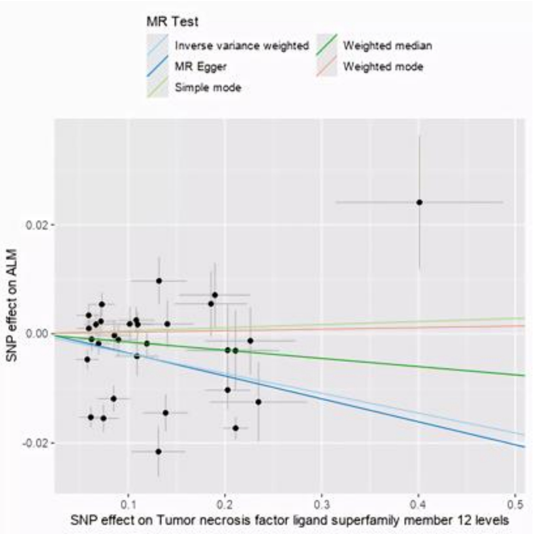

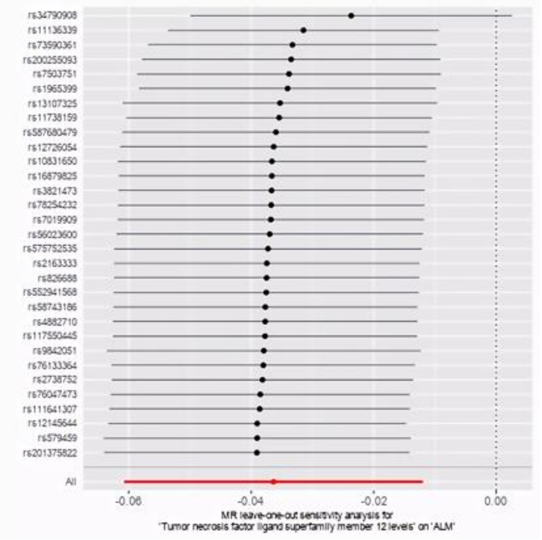

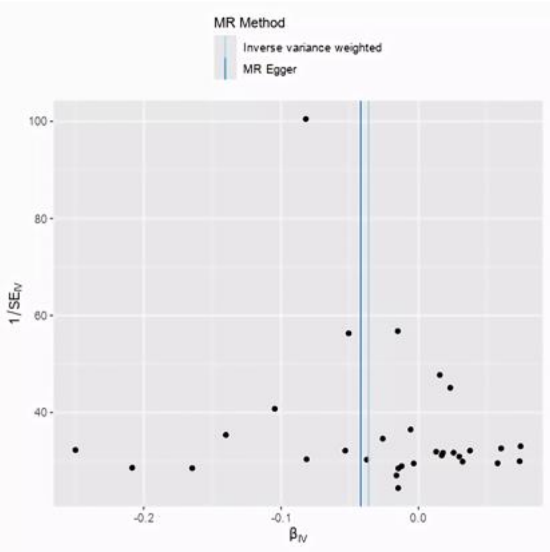

Results of LGS

SIRT2

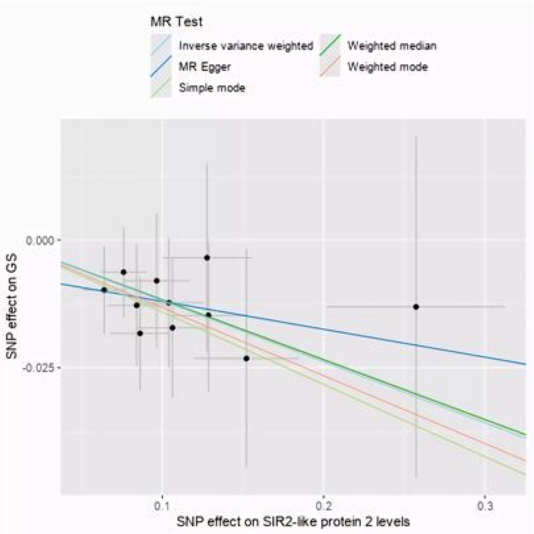

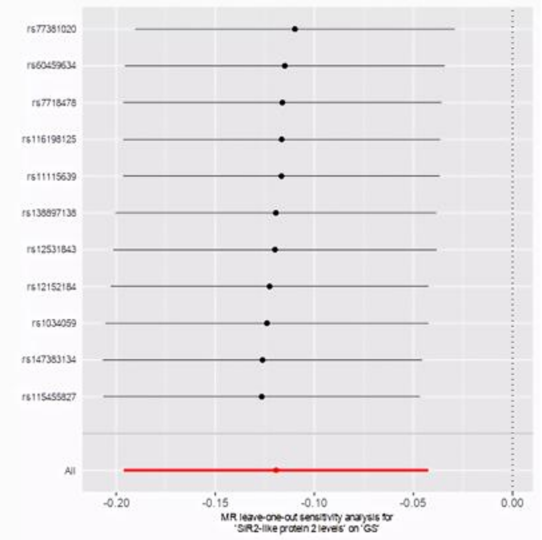

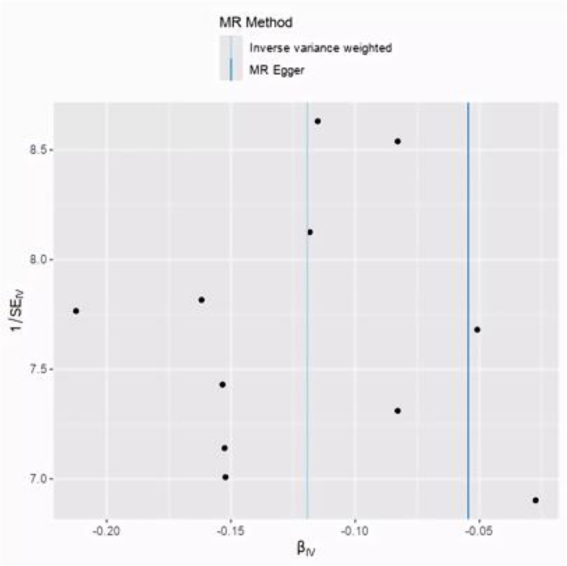
MR Method
Inverse variance weighted
MR Egger
8.5
8.0
7.5
7.0
1/SE$_{IV}$
-0.20
-0.15
-0.10
-0.05
β$_{IV}$

LTA

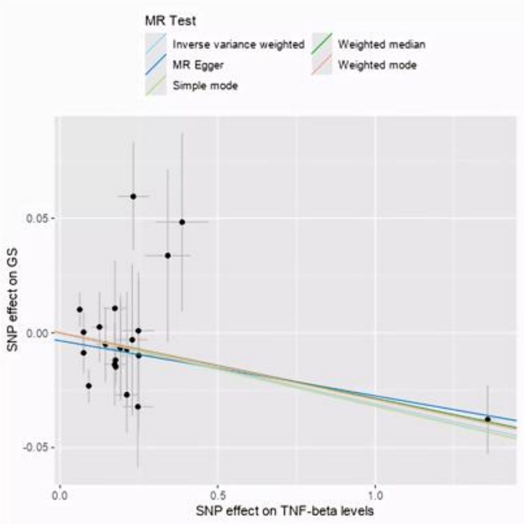
MR Test
Inverse variance weighted
MR Egger
Simple mode
Weighted median
Weighted mode
0.05
0.00
-0.05
SNP effect on GS
0.0
0.5
1.0
SNP effect on TNF-beta levels

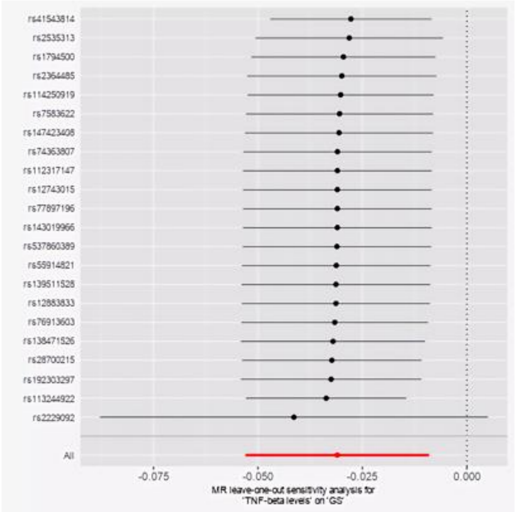
rs41543814
rs2535313
rs1794500
rs2364485
rs114250919
rs7583622
rs147423408
rs74363807
rs112317147
rs12743015
rs77897196
rs143019966
rs537860389
rs55914821
rs139511528
rs12883833
rs76913603
rs138471526
rs28700215
rs192303297
rs113244922
rs2229092
All
-0.075
-0.050
-0.025
0.000
MR leave-one-out sensitivity analysis for
'TNF-beta levels' on 'GS'

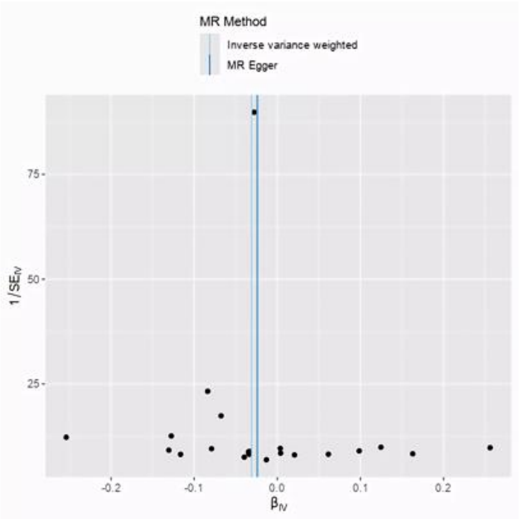
MR Method
Inverse variance weighted
MR Egger
75
50
25
1/SE$_{IV}$
-0.2
-0.1
0.0
0.1
0.2
β$_{IV}$

VEGFA

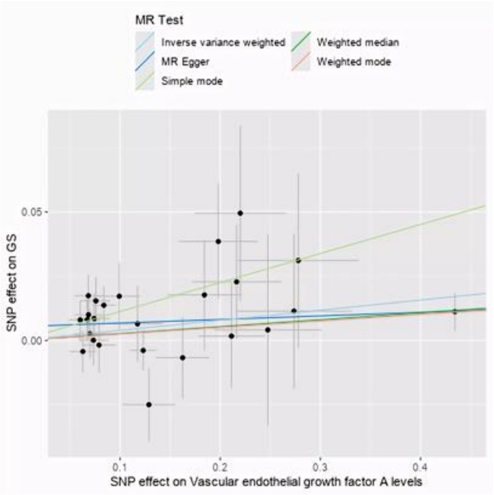

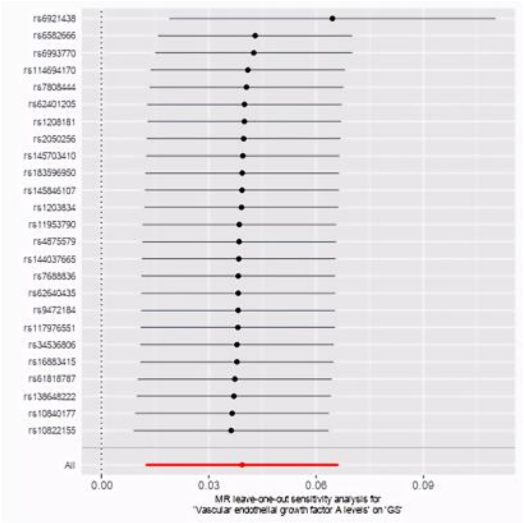

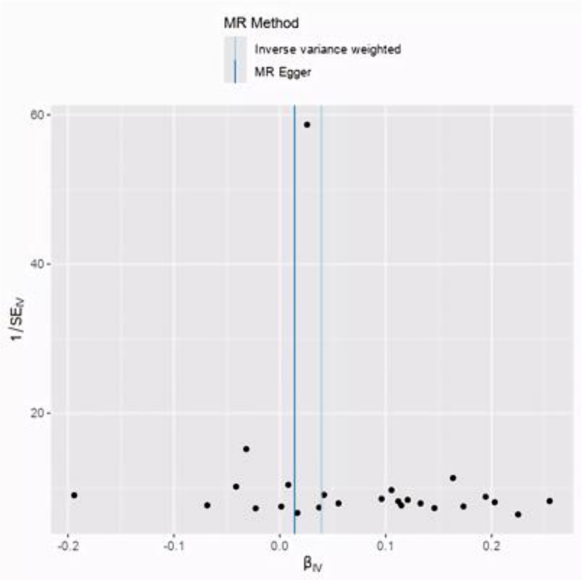

Results of WP

CD40

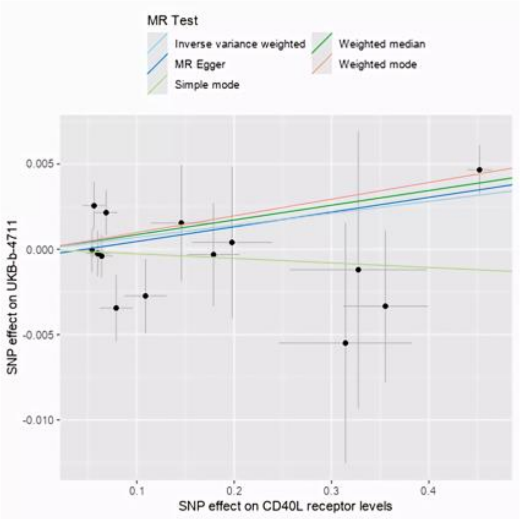

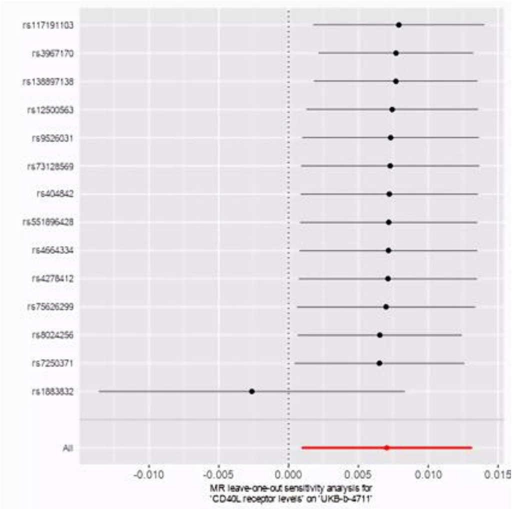

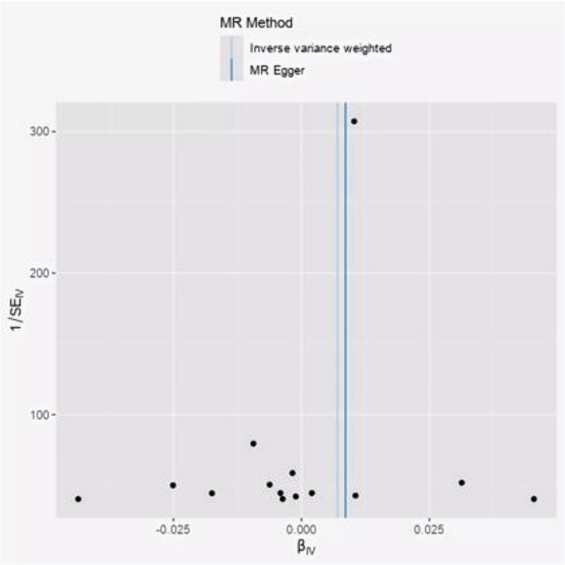
MR Method
Inverse variance weighted
MR Egger
300
200
100
1/SE$_{IV}$
-0.025
0.000
0.025
β$_{IV}$

IL24

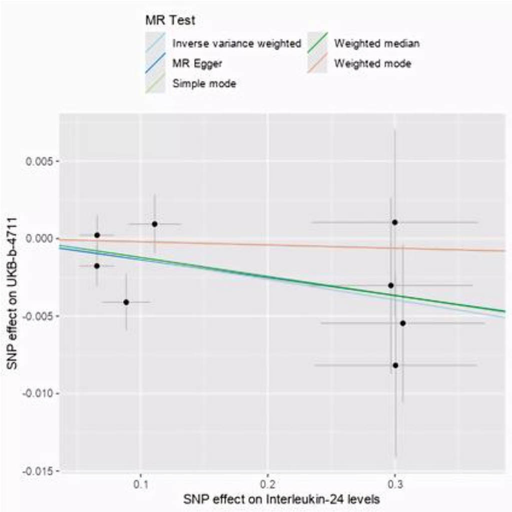
MR Test
Inverse variance weighted
MR Egger
Simple mode
Weighted median
Weighted mode
0.005
0.000
-0.005
-0.010
-0.015
SNP effect on UKB-b-4711
0.1
0.2
0.3
SNP effect on Interleukin-24 levels

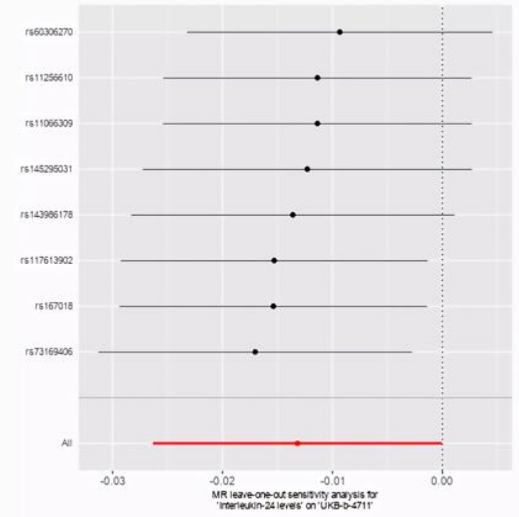
rs60306270
rs11256610
rs11066309
rs145295031
rs143986178
rs117613902
rs167018
rs73169406
All
-0.03
-0.02
-0.01
0.00
MR leave-one-out sensitivity analysis for
'Interleukin-24 levels' on 'UKB-b-4711'

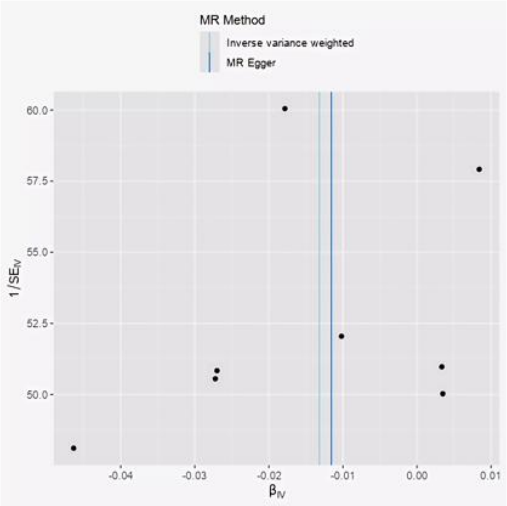
MR Method
Inverse variance weighted
MR Egger
60.0
57.5
55.0
52.5
50.0
1/SE$_{IV}$
-0.04
-0.03
-0.02
-0.01
0.00
0.01
β$_{IV}$

IL7

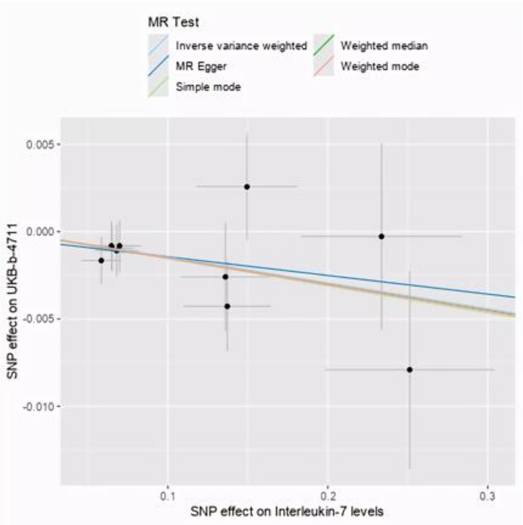
MR Test
Inverse variance weighted
MR Egger
Simple mode
Weighted median
Weighted mode
SNP effect on UKB-b-4711
SNP effect on Interleukin-7 levels

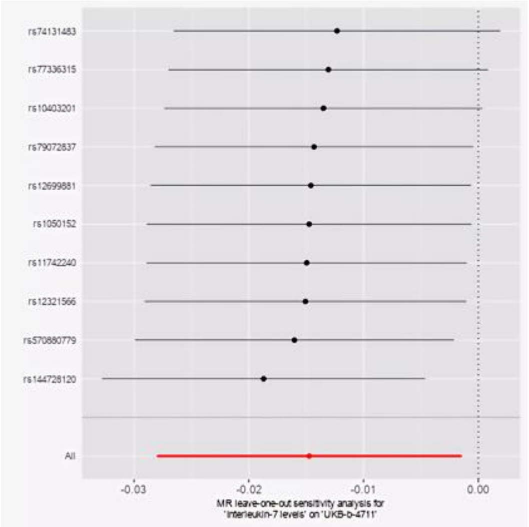
rs74131483
rs77336315
rs10403201
rs79072837
rs12699881
rs1050152
rs11742240
rs12321566
rs570880779
rs144728120
All
MR leave-one-out sensitivity analysis for 'Interleukin-7 levels' on 'UKB-b-4711'

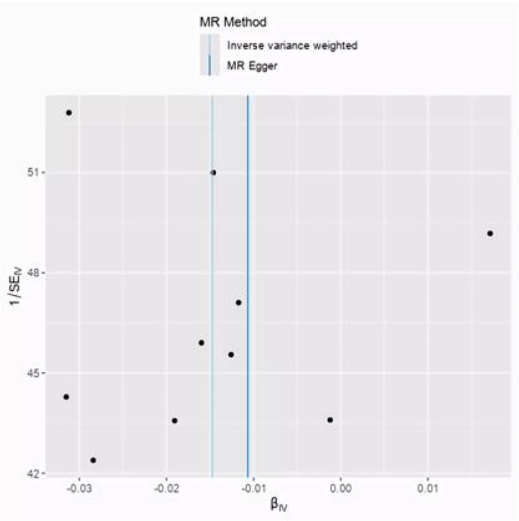
MR Method
Inverse variance weighted
MR Egger
1/SE_IV
β_IV

SULT1A1

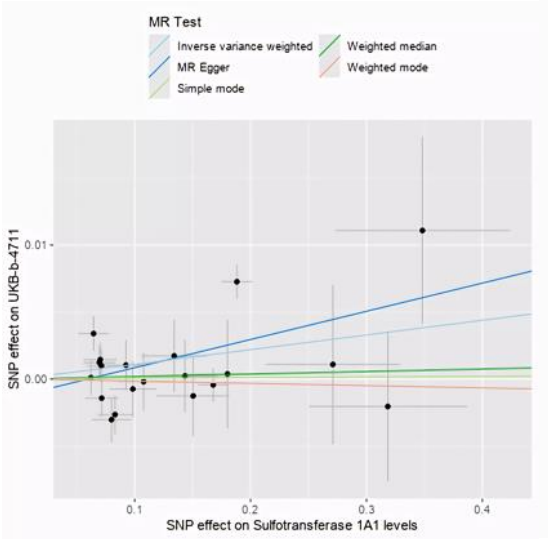
MR Test
Inverse variance weighted
MR Egger
Simple mode
Weighted median
Weighted mode
SNP effect on UKB-b-4711
SNP effect on Sulfotransferase 1A1 levels

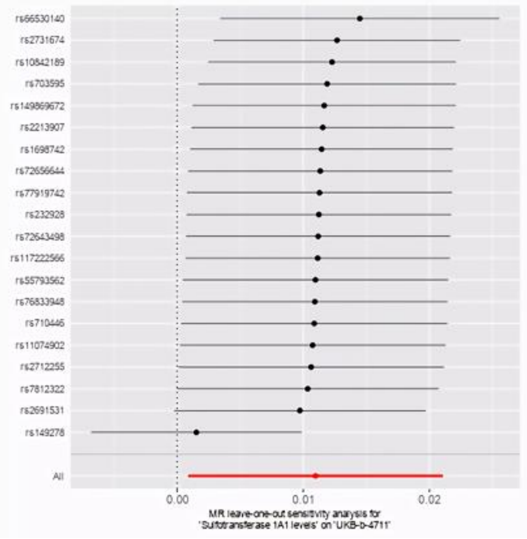
All
MR leave-one-out sensitivity analysis for 'Sulfotransferase 1A1 levels' on 'UKB-b-4711'

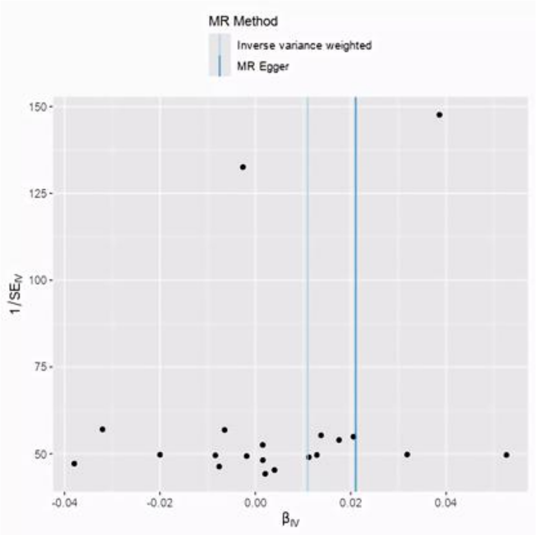
MR Method
Inverse variance weighted
MR Egger
1/SE$_{IV}$
β$_{IV}$

LTA

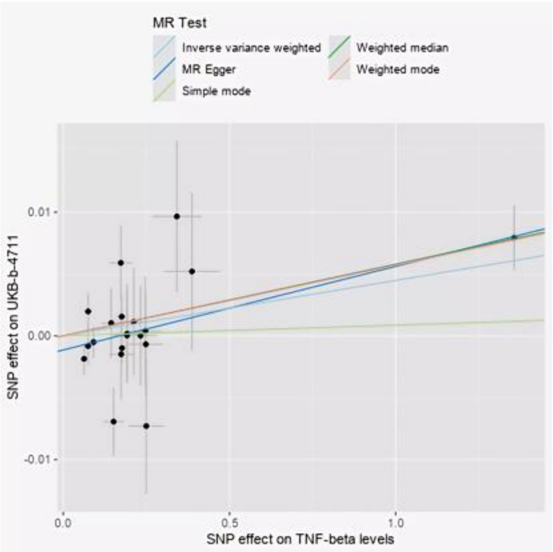
MR Test
Inverse variance weighted
MR Egger
Simple mode
Weighted median
Weighted mode
SNP effect on UKB-b-4711
SNP effect on TNF-beta levels

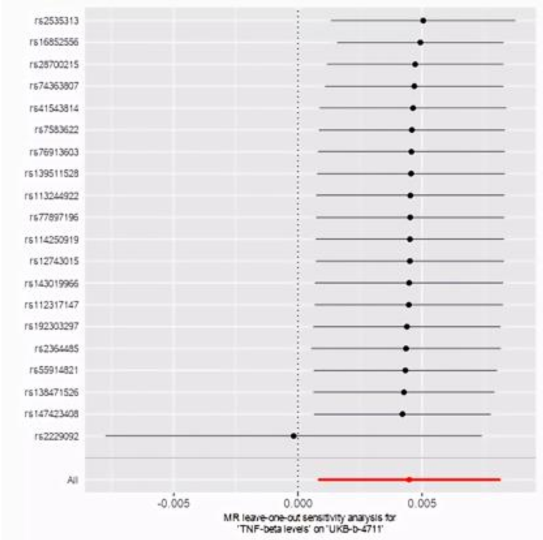
rs2535313
rs16852556
rs28700215
rs74363807
rs41543814
rs7583622
rs76913603
rs139511528
rs113244922
rs77897196
rs114250919
rs12743015
rs143019966
rs112317147
rs192303297
rs2364485
rs55914821
rs138471526
rs147423408
rs2229092
All
MR leave-one-out sensitivity analysis for
'TNF-beta levels' on 'UKB-b-4711'

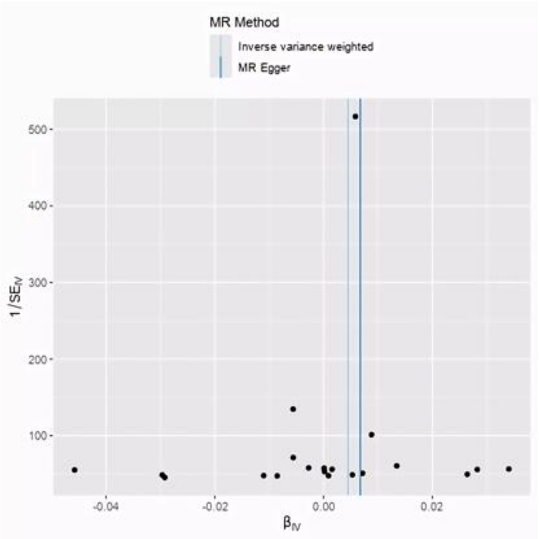
MR Method
Inverse variance weighted
MR Egger
1/SE$_{IV}$
β$_{IV}$
