# Supplementary figures and images for: Causal Associations of Inflammatory Cytokines With Osteosarcopenia: Insights From Mendelian Randomization and Single Cell Analysis
Source: Mediators Inflamm. 2025 Apr 3;2025:6005225. doi: 10.1155/mi/6005225 (PMC11986192; doi:10.1155/mi/6005225)

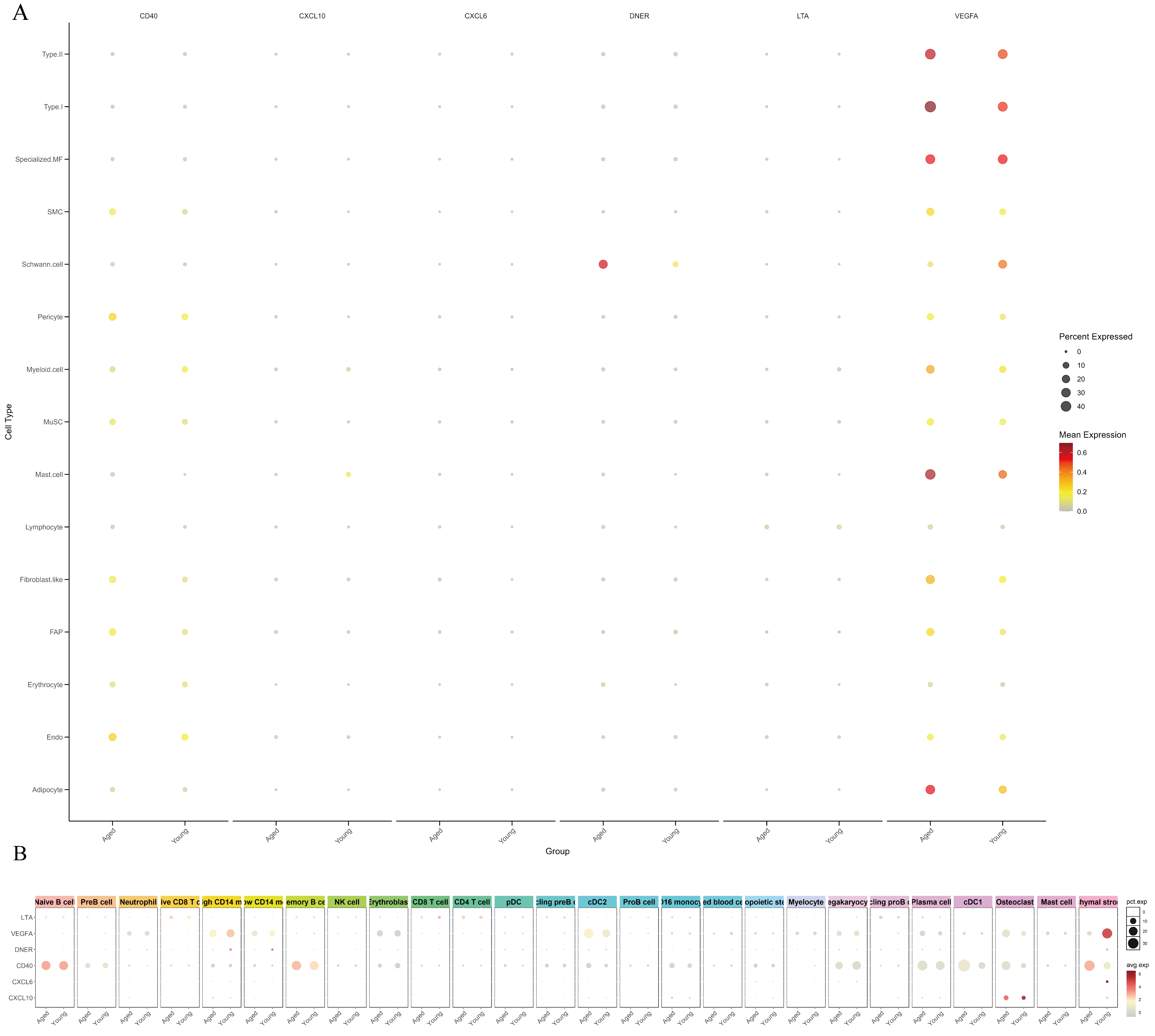

Supplement: Supporting Information 4 — Figure S3: Expression of candidate cytokines in different cell types between subgroups. (A) Results of human skeletal muscle. (B) Results of human bone marrow. [file 6005225.f4.tif]
